# Phenotypic Resistance to Beta-Lactam Antimicrobials in Bacterial Isolates from Different Animal Populations in Texas and Oklahoma, 2018–2024: A Retrospective Study

**DOI:** 10.3390/antibiotics15090836

**Published:** 2026-08-28

**Authors:** Ibrahim Idris, Babafela Awosile

**Affiliations:** School of Veterinary Medicine, Texas Tech University, Amarillo, TX 79106, USA; ibidris@ttu.edu

**Keywords:** antimicrobial resistance, surveillance, beta-lactams, veterinary diagnostic laboratory, animal species, bacterial isolates

## Abstract

**Background/Objectives**: Beta-lactam antimicrobials are among the most prescribed drugs in veterinary medicine, yet comprehensive epidemiological assessments of resistance across animal species, bacterial taxa, and clinical specimen types remain limited. This study aimed to characterize the epidemiology of phenotypic beta-lactam resistance among bacterial isolates submitted to veterinary diagnostic laboratories in Texas and Oklahoma from 2018 to 2024 and to identify factors and organism–specimen–antimicrobial combinations associated with the overall resistance burden. **Methods**: A retrospective analysis was conducted using antimicrobial susceptibility testing data from 166,686 bacterial isolates collected between 2018 and 2024. Following data cleaning and quality assessment, 789,871 susceptibility observations across seven beta-lactam antimicrobials were analyzed. Descriptive analyses, univariable and multivariable logistic regression, bacterial diversity analysis, and resistance burden ranking were performed to characterize resistance patterns and identify factors associated with resistance. **Results**: Resistance varied markedly among the seven beta-lactam antimicrobials. Amoxicillin showed the highest resistance prevalence (56.3%), whereas ceftazidime (11.2%) and imipenem (17.2%) had the lowest. Resistance differed substantially across animal categories, bacterial taxa, and clinical specimen groups. Multivariable analyses identified state, animal category, bacterial group, and clinical specimen group as factors independently associated with resistance, while no consistent temporal differences were detected across 2018–2024 after multivariable adjustment. After sample-size standardization, bacterial richness was highest among zoo/wildlife/aquatic animals, while diversity and evenness varied across animal categories and antimicrobials. *Staphylococcus pseudintermedius* isolated from skin specimens and tested against amoxicillin contributed the greatest overall resistance burden. **Conclusions**: Beta-lactam resistance exhibited substantial epidemiological heterogeneity across host species, bacterial taxa, clinical specimens, and states. These findings provide an evidence base for antimicrobial stewardship, resistance surveillance, and empirical antimicrobial therapy in veterinary medicine.

## 1. Introduction

Antimicrobial resistance (AMR) is one of the greatest risks to public and animal health. In 2019, bacterial AMR was estimated to be associated with 4.95 million deaths worldwide, including 1.27 million deaths directly attributed to resistant infections. This burden is predicted to increase substantially by 2050 without effective interventions [1,2]. AMR is broadly acknowledged as a One Health concern that needs integrated surveillance and control across all sectors as resistant bacteria and antimicrobial resistance determinants travel among humans, animals and the environment [3]. Antimicrobial use remains a necessity in veterinary practice to preserve animal health and wellbeing, but antimicrobial exposure contributes to the selection and spread of resistant bacteria of veterinary and public health relevance [4]. The beta-lactam class encompasses penicillins, cephalosporins, and carbapenems and remains the most frequently prescribed antimicrobial class in companion-animal veterinary medicine, accounting for roughly two-thirds of antimicrobial prescriptions in dogs and cats in primary-care practice [5,6], largely because aminopenicillins and first-generation cephalosporins are inexpensive, well tolerated, and available in convenient formulations [6]. This heavy, often empirical use is reflected in resistance data: in US clinical *Escherichia coli* from dogs and cats, resistance to ampicillin (≈52.7%), amoxicillin–clavulanate (≈45%), and cephalothin (>95%) is consistently the highest among beta-lactams [7]. Resistance to these agents is also rising over time in both clinical companion-animal isolates [8] and commensal food-animal *E. coli* recovered at slaughter, where NARMS surveillance has documented significant increasing temporal trends in ampicillin, amoxicillin–clavulanate, and ceftriaxone resistance [9]. Because antimicrobial consumption in animals remains substantial and continues to select for resistance [4], and because later-generation cephalosporins and carbapenems are classified as critically important for human medicine [10], understanding how beta-lactam resistance is distributed across hosts, organisms, and specimens is a priority for stewardship. The emergence and dissemination of extended-spectrum β-lactamase (ESBL)-producing Enterobacteriaceae across livestock, food products, humans, and the environment further underscore the One Health significance of beta-lactam resistance and the need for integrated surveillance across these interconnected sectors [11]. Recent literature further emphasizes that AMR and ESBL-producing Enterobacteriaceae in animal and food systems require coordinated One Health surveillance and antimicrobial stewardship across interconnected human, animal, food, and environmental sectors [11,12].

Surveillance underpins stewardship, yet its coverage is uneven across animal populations. Food-producing animals are monitored through integrated national programs, whereas companion animals remain largely absent from mandatory frameworks despite their close human contact [13,14]. Where companion-animal surveillance has been attempted, it has increasingly drawn on data generated routinely by veterinary diagnostic laboratories, which provide broad state and temporal coverage: recent US analyses have used such data to quantify resistance and identify host, specimen, and organism predictors in canine *E. coli* [15] and *staphylococci* [16] and to demonstrate the feasibility of commercial-laboratory data for national AMR estimation across millions of susceptibility results [14]. These studies, however, have generally examined one organism, one host, or one specimen type at a time. Few have exploited diagnostic-laboratory data across multiple host species, bacterial taxa, and specimen sources simultaneously, which is precisely the gap the present study addresses. Thus, veterinary diagnostic laboratory data represent an unexplored but valuable source for defining regional resistance trends in a variety of animal species. Previous epidemiological studies have reported a significant variation in beta-lactam resistance depending on bacterial species, host animal, specimen source and geographic location [16,17,18,19,20]. However, most studies have been confined to one bacterial species, antimicrobial, host population or healthcare setting and as such have limited the potential for direct comparison of findings across the wider veterinary population encountered in routine diagnostic practice.

Thus, a deeper epidemiological study involving multiple species of animals, bacterial taxa, types of clinical samples, antimicrobial and state locations is required to better understand the distribution and determinants of beta-lactam resistance. These analyses will not only improve the understanding of resistance epidemiology but also help to identify the organism–specimen–antimicrobial combinations that contribute most substantially to the total burden of resistance, thereby aiding in surveillance prioritization and antimicrobial stewardship. The aim of this study is to characterize the epidemiology of beta-lactam antimicrobial resistance in bacterial isolates submitted to veterinary diagnostic laboratories in Texas and Oklahoma during 2018–2024 by assessing resistance patterns by animal hosts, bacterial taxa, clinical specimen types, and states, and determining the major factors and organism–specimen–antimicrobial combinations contributing to the overall resistance burden.

## 2. Results

### 2.1. Study Population and Analytical Dataset

The total study population after data cleaning and transformation was 166,686 bacterial isolates submitted for antimicrobial susceptibility testing (Table 1). These isolates consisted of 80 animal species, which were classified into seven analytical animal groups (Supplementary Table S1), 135 bacterial taxa, which were classified into 26 analytical bacterial groups (Supplementary Table S3), and 108 clinical specimen types, which were classified into 11 analytical specimen categories (Supplementary Table S4). The most frequently represented animal category was companion animals (95.9%). The most isolated bacterial taxa were *Escherichia coli* and *Staphylococcus pseudintermedius*, and the most common clinical specimen types were urine, skin and ear (Supplementary Tables S3 and S4). The final analytical dataset used in all subsequent analyses consisted of 789,871 antimicrobial susceptibility observations for the seven retained beta-lactam antimicrobials after the removal of non-binary susceptibility outcomes and the application of the predefined antimicrobial selection criteria (Table 1; Supplementary Table S2).

### 2.2. Antimicrobial Selection and Testing Completeness

The extent to which the 19 beta-lactam antimicrobials included in the raw dataset were tested varied greatly, from 0% (Ticarcillin, Ticarcillin/Clavulanic Acid, Piperacillin/Tazobactam, Cefalothin, Cefoxitin, and Penicillin G, none of which had interpretable results) to 79.4% for Amoxicillin/Clavulanate (Supplementary Table S2). The seven antimicrobials with the highest interpretable testing coverage were retained for the primary analysis; the lowest coverage among these retained agents was 48.8%, which therefore represents a data-derived cutoff rather than an externally established clinical threshold: Amoxicillin/Clavulanate (79.4%), Amoxicillin (72.5%), Cefpodoxime (70.8%), Cefovecin (69.1%), Imipenem (68.1%), Cefalexin (67.3%), and Ceftazidime (48.8%). The remaining 12 antimicrobials, including ceftiofur (34.9%), penicillin (32.6%), and cefotaxime (30.6%), were excluded from the primary cross-population analysis because their lower and likely panel-specific testing coverage would increase sparsity and limit comparability across animal groups (Supplementary Table S2). These excluded agents remain clinically relevant, particularly in food-animal practice, and their exclusion may bias cross-host comparisons toward antimicrobials more commonly represented on companion-animal testing panels.

### 2.3. Resistance Prevalence of Beta-Lactam Antimicrobials

**Figure 1:**
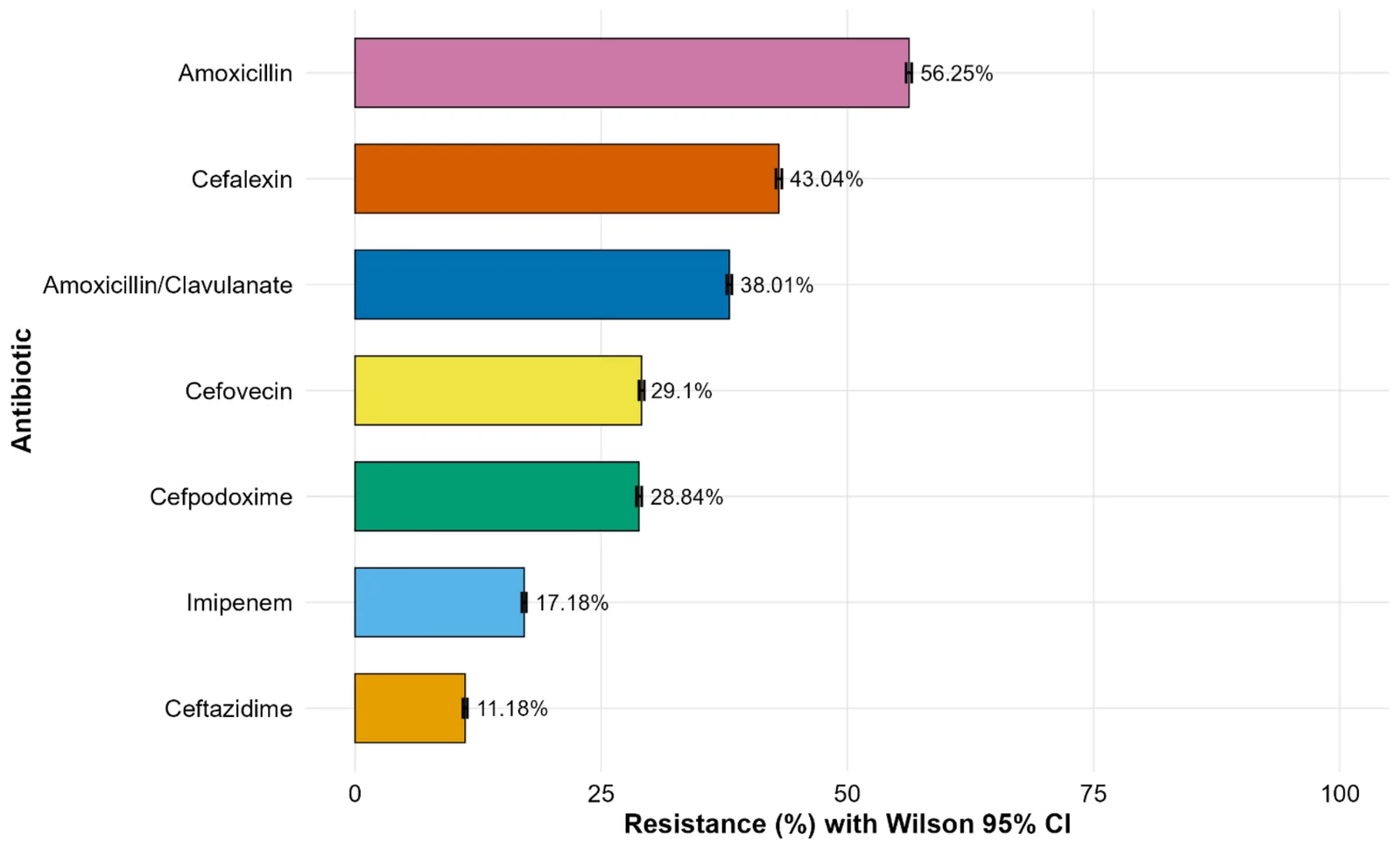
Overall beta-lactam resistance prevalence among bacterial isolates from Texas and Oklahoma (2018–2024). Bars represent resistance prevalence; error bars indicate Wilson 95% confidence intervals.

Overall resistance prevalence varied widely across the seven beta-lactam antimicrobials that were kept (Table 2, Figure 1). Within the retained beta-lactam drugs there was a distinct gradient in resistance prevalence with >5-fold difference between the least and most resistant antimicrobials. The highest resistance was observed for amoxicillin (56.3%), followed by cefalexin (43.0%) and amoxicillin/clavulanate (38.0%). Resistance prevalence was intermediate for cefovecin (29.1%) and cefpodoxime (28.8%). Imipenem (17.2%) and ceftazidime (11.2%) had the lowest resistance frequency. Two antimicrobials had resistance prevalence over 40%, three antimicrobials had resistance between 20% and 40%, while imipenem and ceftazidime had resistance under 20%. Overall resistance decreased from amoxicillin to ceftazidime, with large variation in resistance for the remaining beta-lactam antimicrobials. The comparatively low observed resistance to imipenem and ceftazidime should be interpreted within the tested diagnostic population and does not imply uniformly high susceptibility across veterinary populations, because testing frequency, organism composition, clinical indication, and panel selection may influence these estimates.

### 2.4. Resistance Patterns by Animal Category

**Figure 2:**
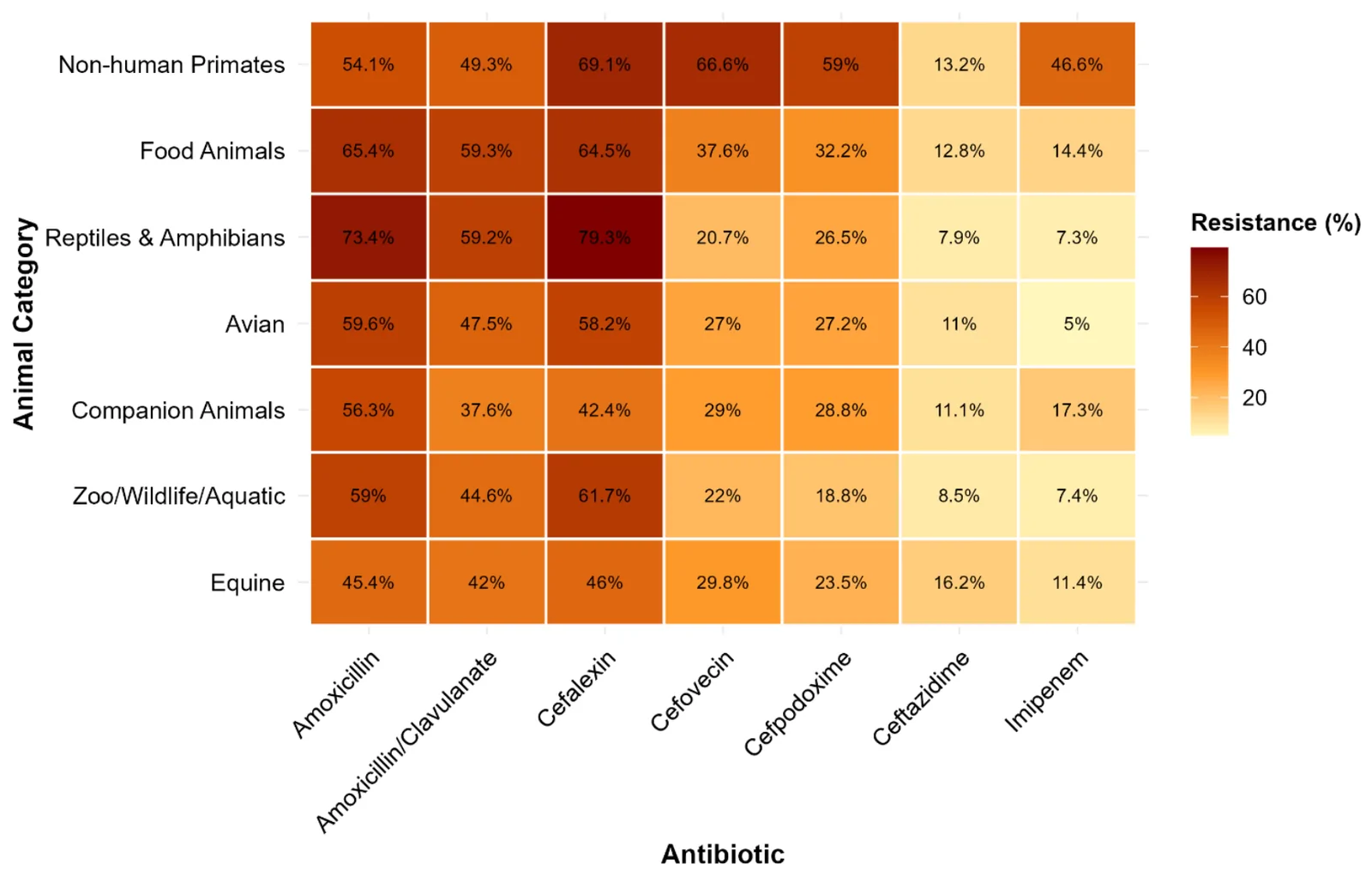
Heatmap of beta-lactam resistance prevalence (%) by animal category and antimicrobial. Cell values indicate resistance prevalence.

Figure 2 demonstrates the large variation in prevalence of resistance among animal categories and beta-lactam antimicrobials. The heatmap revealed a great degree of variation in resistance between animal species, with no single animal category consistently showing the highest or lowest resistance for all antimicrobials. However, there was a clear clustering of resistance patterns, suggesting that host category influenced the resistance profiles of the retained beta-lactam drugs. Non-human primates consistently have some of the greatest levels of resistance across most beta-lactam antimicrobials, particularly for cefalexin (69.1%), cefovecin (66.6%), and cefpodoxime (59.0%). Companion animals and equine and avian species showed moderate resistance to the retained antimicrobials, whereas food animals, reptiles and amphibians also showed comparatively high resistance to amoxicillin, amoxicillin/clavulanate and cefalexin. On the contrary, zoo/wildlife/aquatic animals had relatively reduced resistance to most of the antimicrobials, especially imipenem and ceftazidime. Amoxicillin had the highest rate of resistance for almost all animal types, while ceftazidime and imipenem were the least affected antimicrobials. Amoxicillin/clavulanate, cefalexin, cefovecin and cefpodoxime had intermediate resistance but differed in different animal groups. The heatmap also showed that resistance patterns were not consistent between animal species, with different patterns for each antimicrobial, suggesting that beta-lactam resistance varies throughout animal populations.

### 2.5. Resistance Patterns by Bacterial Taxon

**Figure 3:**
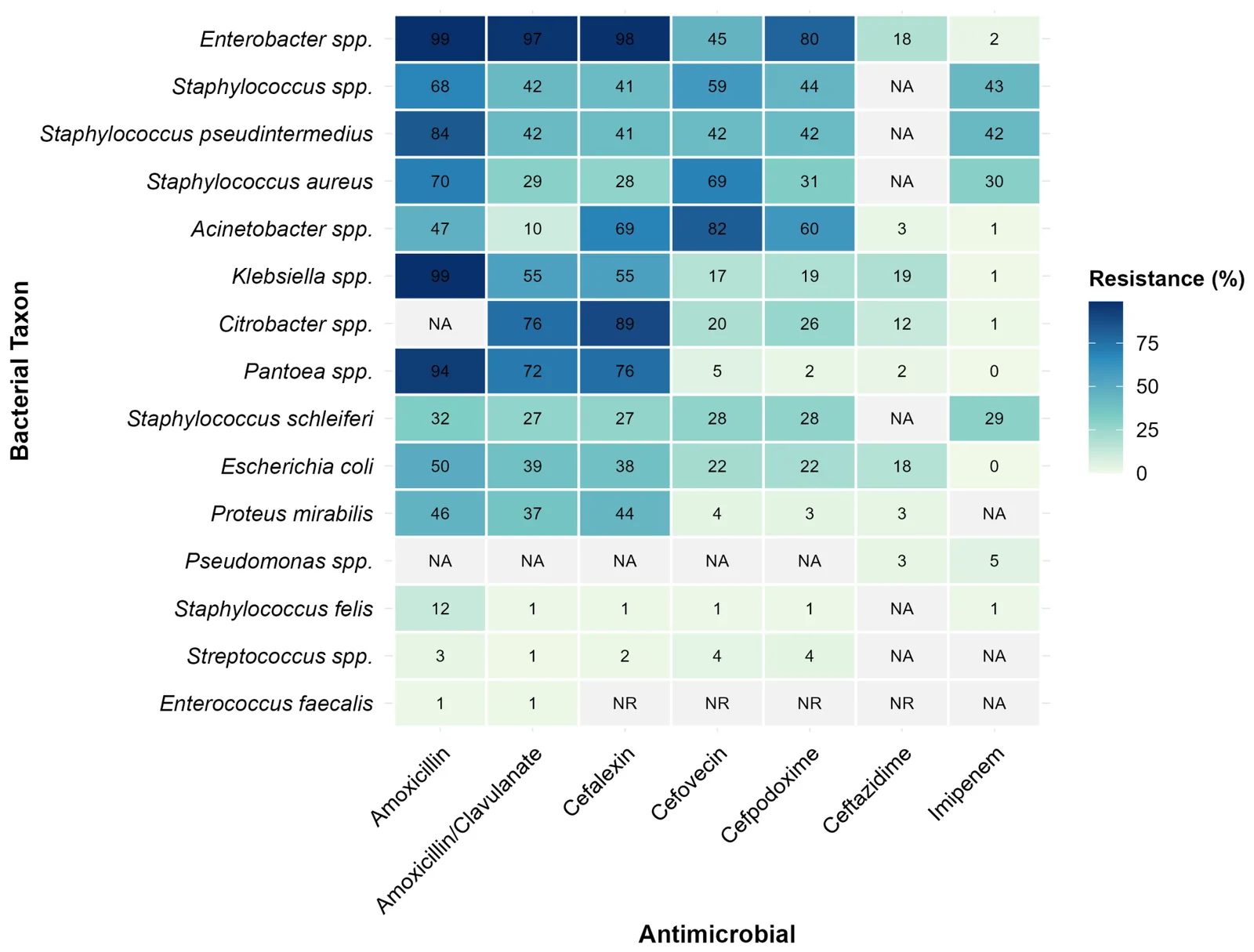
Heatmap of beta-lactam resistance prevalence (%) by bacterial taxon and antimicrobial. NA indicates <30 interpretable observations, and NR indicates *Enterococcus*–cephalosporin combinations with an expected resistant phenotype that were not interpreted as acquired resistance.

Resistance profiles varied substantially among bacterial taxa, with antimicrobial-specific patterns across Gram-negative and Gram-positive organisms (Figure 3). To avoid unstable interpretation of sparsely tested organism–antimicrobial combinations, heatmap cells based on fewer than 30 interpretable binary susceptibility observations were not estimated and are displayed as NA. *Escherichia coli* showed approximately 50% resistance to amoxicillin and lower resistance to the other retained beta-lactam antimicrobials. *Staphylococcus pseudintermedius*, the most frequently represented staphylococcal species, showed moderate-to-high resistance across the retained agents, with the highest resistance to amoxicillin. *Enterococcus*–cephalosporin combinations were not interpreted as acquired susceptibility or resistance because enterococci have an expected resistant phenotype to cephalosporins. Likewise, the *Proteus mirabilis*–imipenem cell was not estimated because only 16 interpretable binary observations were available, in addition to the breakpoint-sensitive interpretation of imipenem for Proteus spp. These restrictions reduce the risk of overinterpreting sparse or biologically inappropriate organism–drug combinations. Detailed taxon-specific resistance summaries are provided in Supplementary Tables S5–S11. Interpretation of organism-specific AST results followed the principle that susceptibility categories must be considered in the context of the organism, host, infection site, and applicable breakpoint guidance [21,22].

### 2.6. Resistance Patterns by Clinical Specimen Type

**Figure 4:**
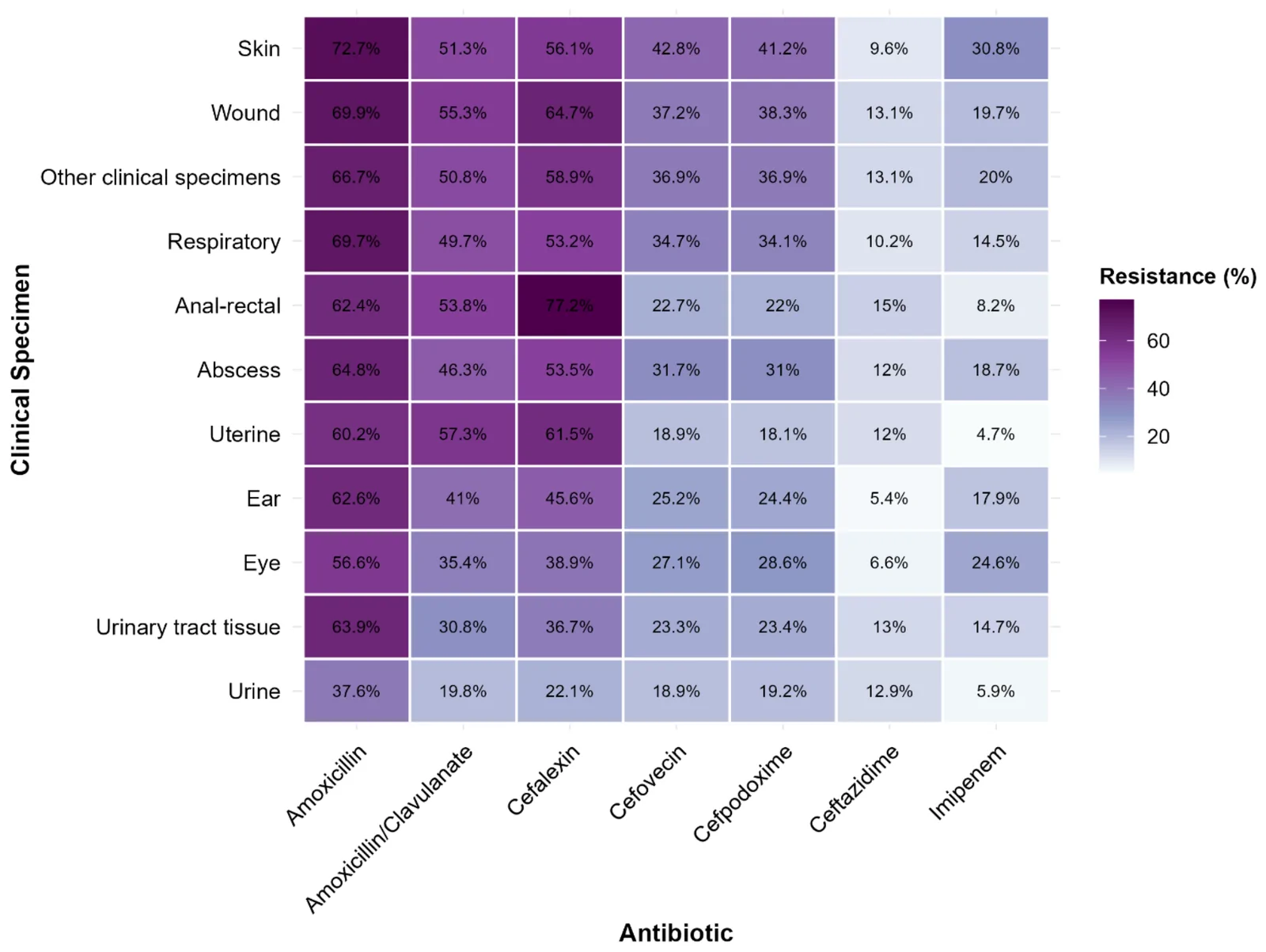
Heatmap of beta-lactam resistance prevalence (%) by clinical specimen group and antimicrobial. Cell values indicate resistance prevalence within each clinical specimen group–antimicrobial combination.

Clinical specimen categories represented specimen sources rather than taxonomic groups and included bacterial isolates from multiple taxa; taxon-specific resistance patterns are presented separately in Section 2.5 and Supplementary Tables S5–S11. For each of the seven retained beta-lactam antimicrobials, resistance prevalence varied substantially among clinical specimen groups (Figure 4). Heatmap analysis demonstrated distinct resistance patterns across clinical specimen sources, indicating substantial variation in resistance profiles among isolates originating from different anatomical sites. Prevalence of resistance differed significantly among clinical specimen groups and the seven retained beta-lactam antimicrobials (Figure 4; Supplementary Tables S5–S11). For most retained beta-lactam antimicrobials, the highest resistance prevalence was observed in skin, wound, and other clinical specimens, whereas the lowest prevalence was frequently observed in urine specimens. Amoxicillin had the highest resistance rate in almost all specimen groups with 72.7% resistance in skin specimens and 69.9% in wound specimens. Cefalexin also had strong resistance, especially in anal–rectal (77.2%) and wound (64.7%) specimens. Resistance to cefovecin and cefpodoxime was generally intermediate across specimen categories, with stronger resistance in skin and wound specimens than in urine. Conversely, ceftazidime and imipenem revealed the lowest consistent resistance prevalence across all classes of clinical specimens, with resistance remaining <20% for most specimen types. Overall, the heatmap revealed distinctive resistance patterns for each specimen that indicated that antimicrobial resistance differed considerably by the clinical source of bacterial isolation. Amoxicillin generally showed the highest resistance prevalence across specimen groups, whereas ceftazidime and imipenem showed the lowest prevalence irrespective of specimen source.

### 2.7. Univariable Logistic Regression

Univariable logistic regression analyses were performed for each of the seven retained beta-lactam antimicrobials separately to assess the unadjusted associations between antimicrobial resistance and year, state, animal category, bacterial group and clinical specimen group (Supplementary Table S12). Year of isolate submission was not significantly linked with resistance for any antimicrobial. Odds ratios were continuously close to unity across the 2018–2024 research period, demonstrating no observable temporal trend in resistance.

In contrast, most antimicrobials had substantial unadjusted associations with beta-lactam resistance for states, animal category, bacterial group and clinical specimen group. Oklahoma isolates were usually less likely to be resistant than Texas isolates, however the strength of the link varied per antimicrobial and was minor in magnitude (amoxicillin OR = 0.96). Animal category also showed considerable variance, with several groups being significantly different from companion animals based on the antimicrobial under evaluation. The strongest crude relationships were for bacterial groups, with several taxa having significantly higher probabilities of resistance compared to *E. coli*. For example, odds of amoxicillin resistance were 85-fold greater for *Klebsiella* spp. (OR = 85.45) and 91-fold higher for *Enterobacter* spp. (OR = 90.55), although *Enterococcus faecalis* had much lower odds of resistance (OR = 0.014). The clinical specimen group also showed significant variance in resistance to most of the antimicrobials. Full univariable odds ratios, 95% confidence intervals and *p*-values are shown in Supplementary Table S12. Given the large sample size, statistical significance was interpreted together with effect magnitude and confidence intervals; estimates close to the null (OR = 0.96) were considered small associations despite statistical significance.

### 2.8. Multivariable Logistic Regression

In multivariable logistic regression models that simultaneously adjusted for year, state, animal category, bacterial group, and clinical specimen group, 230 of 324 covariate-level comparisons (71.0%) were identified as statistically significant after Benjamini–Hochberg false discovery rate (FDR) correction (Supplementary Table S13). After adjustment for the prespecified covariates, the percentages of significant adjusted relationships ranged from 61.0% for ceftazidime to 81.3% for amoxicillin/clavulanate, showing that beta-lactam resistance was highly linked with host, bacterial, specimen, and state-level variables. Year of isolate submission was not independently associated with resistance for any retained antimicrobial after adjustment; adjusted odds ratios were close to 1.0 across study years. State, however, remained a factor independently associated with resistance. Oklahoma isolates had significantly lower adjusted odds of resistance for all 7 antimicrobials compared with Texas, with adjusted odds ratios of 0.79 for cefpodoxime to 0.88 for ceftazidime, suggesting a state-level difference in resistance persists after adjustment for other explanatory variables. Animal category was also independently associated with resistance; however, the magnitude and direction of these associations differed among antimicrobials. For amoxicillin/clavulanate, cefalexin, cefovecin, cefpodoxime and imipenem, non-human primates had significantly greater adjusted odds of resistance compared to companion animals, with the strongest association observed for imipenem (aOR = 5.14). Conversely, the adjusted odds of resistance among non-human primates did not differ significantly from those among companion animals for amoxicillin or ceftazidime. Avian and zoo/wildlife/aquatic animals tended to have lower adjusted odds of resistance to multiple antimicrobials, while equine and reptiles and amphibians had antimicrobial-specific associations, including significantly higher odds of ceftazidime resistance among equine isolates (aOR = 1.55). Bacterial group was the factor showing the largest antimicrobial-specific associations for beta-lactam resistance in all antimicrobial-specific models. Many bacterial taxa had significantly greater or lower adjusted probabilities of resistance compared with *Escherichia coli*, indicating that resistance varied dramatically by bacterial identity even after controlling host species, state, specimen type and year. The clinical specimen group also remained independently associated with resistance, with several specimen categories having considerably higher adjusted odds than urine, consistent with the descriptive resistance patterns identified in the heatmaps.

Overall, multivariable analyses showed that beta-lactam resistance was not randomly distributed in the study population but was independently associated with states, animal category, bacterial group and clinical specimen group, with little evidence of temporal variation across the study period. Full adjusted odds ratios, 95% confidence intervals, raw *p*-values, FDR-adjusted *p*-values and model coefficients are provided in Supplementary Table S13.

### 2.9. Bacterial Diversity Across Animal Categories

**Figure 5:**
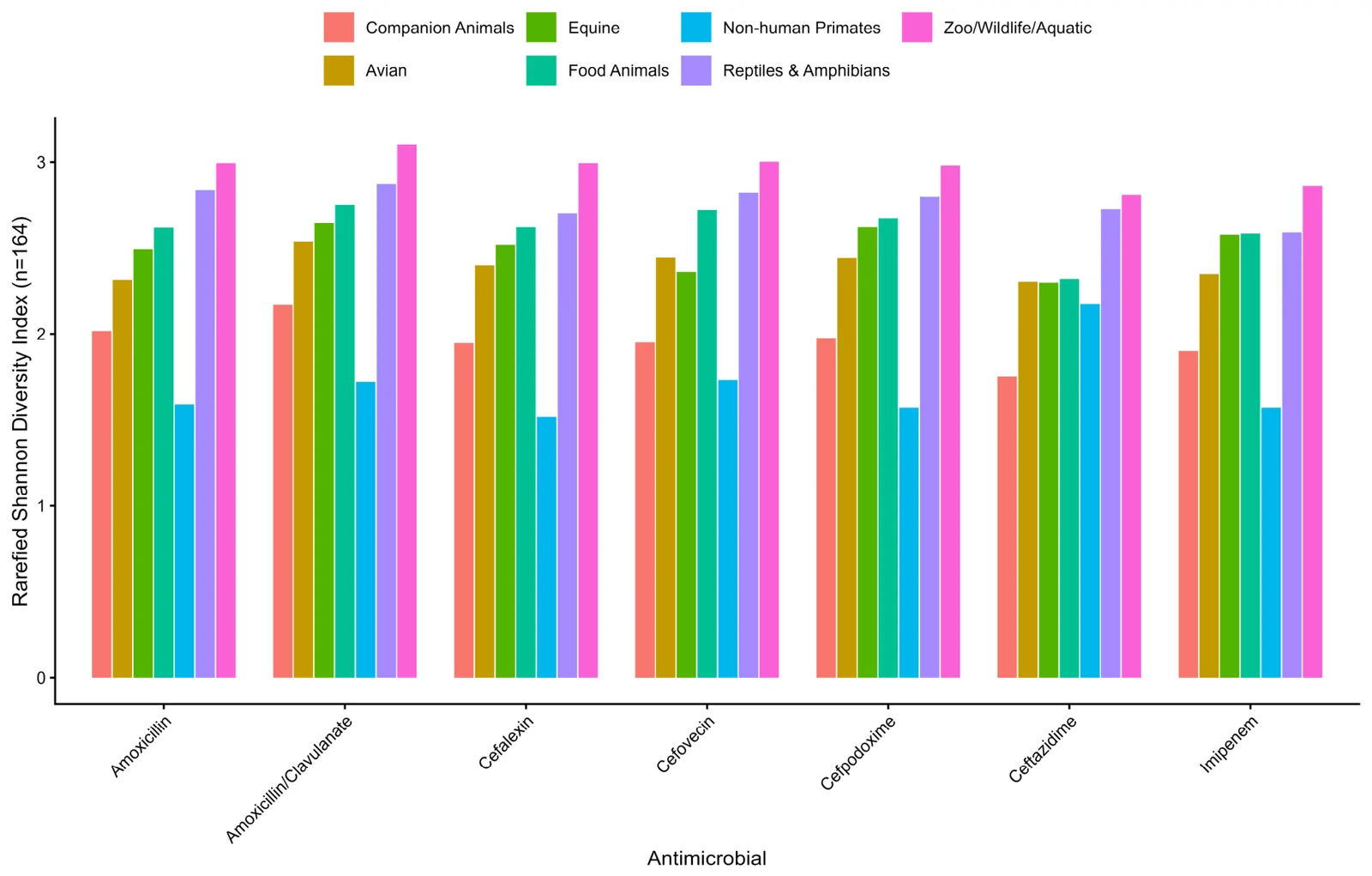
Sample-size-standardized Shannon diversity of bacterial taxa across animal categories and retained beta-lactam antimicrobials. Each animal category–antimicrobial group was rarefied to 164 interpretable binary susceptibility observations using 1000 random subsamples without replacement.

**Figure 6:**
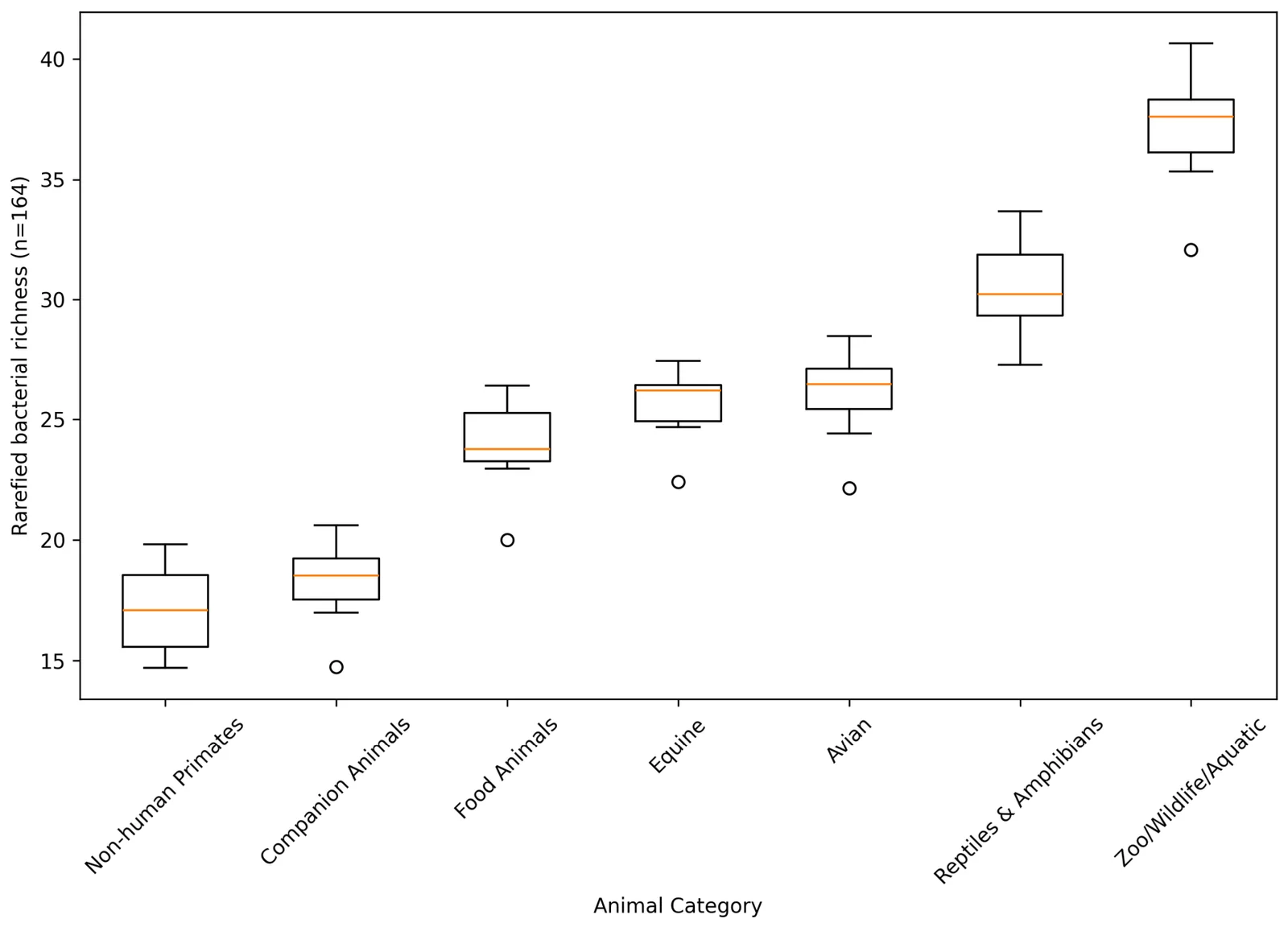
Sample-size-standardized bacterial richness across animal categories. Boxplots summarize mean rarefied richness across the seven retained beta-lactam antimicrobials after standardization to 164 interpretable binary susceptibility observations per animal category–antimicrobial group. Orange lines indicate medians, whiskers extend to values within 1.5 times the interquartile range, and hollow circles indicate observations beyond the whiskers.

After sample-size standardization, bacterial diversity differed across animal categories and retained beta-lactam antimicrobials (Supplementary Table S14; Figure 5 and Figure 6). Individual-based rarefaction standardized every animal category–antimicrobial combination to 164 interpretable binary susceptibility observations, the smallest group size, using 1000 random subsamples without replacement. The highest mean rarefied richness across the seven antimicrobials occurred in zoo/wildlife/aquatic animals (37.03 taxa), followed by reptiles and amphibians (30.51), avian species (26.03), equine (25.53), food animals (23.88), companion animals (18.20), and non-human primates (17.11). Thus, the greater unstandardized richness previously observed among companion animals was attributable in substantial part to sampling intensity and did not persist after rarefaction. Rarefied Shannon diversity was also highest overall in zoo/wildlife/aquatic animals (2.82–3.10 across antimicrobials), whereas companion animals showed lower rarefied Shannon values (1.75–2.17). Food animals retained relatively high evenness (rarefied Pielou 0.77–0.85), while non-human primates showed the lowest overall evenness (0.55–0.73). These standardized analyses indicate that host-category differences in taxonomic diversity should be interpreted independently of the marked imbalance in submission volume.

### 2.10. Resistance Burden Ranking

**Figure 7:**
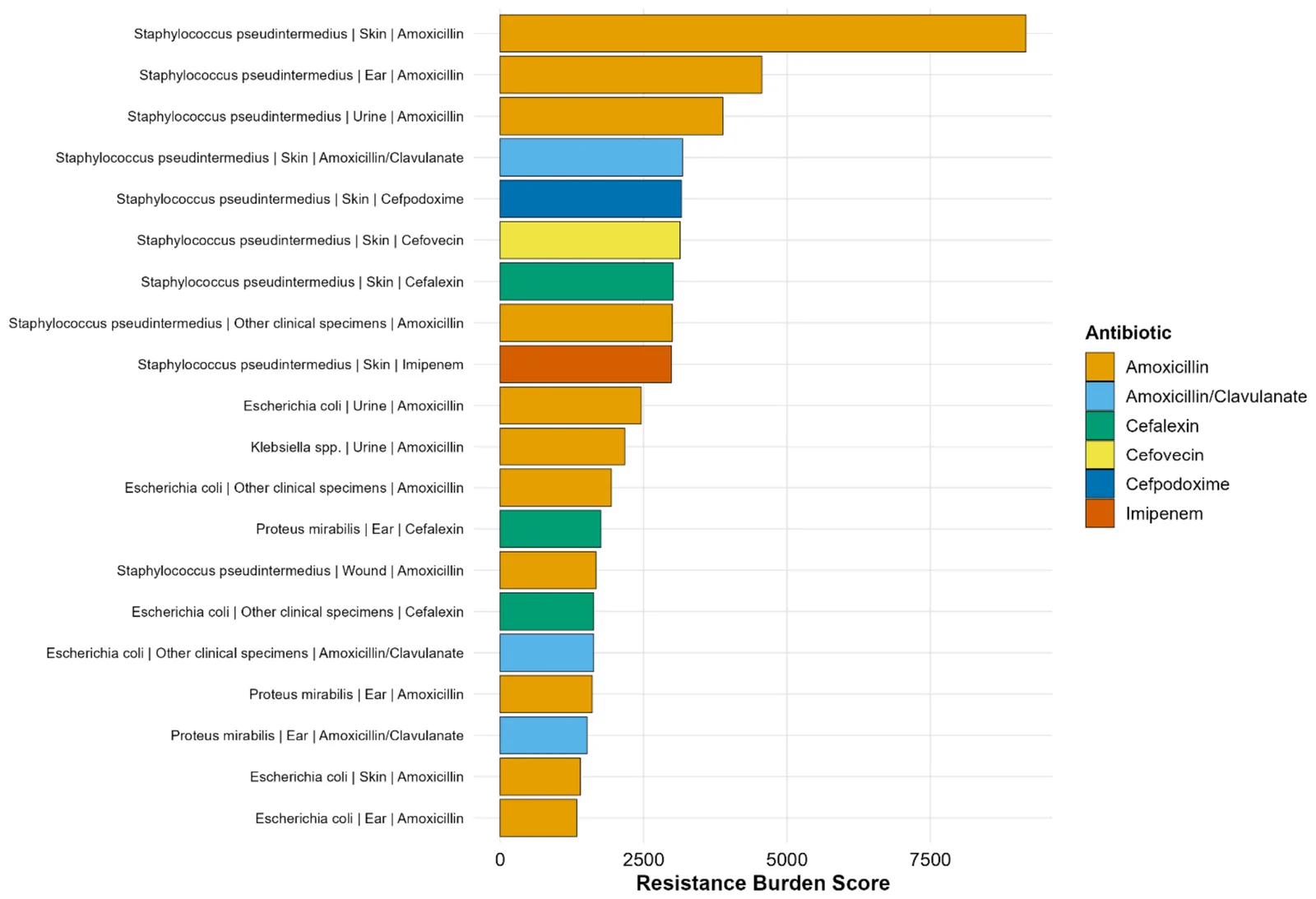
Top 20 bacteria × specimen × antimicrobial combinations ranked by Resistance Burden Score (resistance prevalence multiplied by number of resistant isolates).

The resistance burden ranking demonstrated substantial heterogeneity in the bacterial group–clinical specimen group–antimicrobial combinations contributing to the overall beta-lactam resistance burden (Figure 7; Supplementary Table S15). The 20 highest-ranked combinations were dominated by common bacterial pathogens isolated from frequently submitted clinical specimens. Notably, nine of the twenty highest-ranking combinations involved *Staphylococcus pseudintermedius*, highlighting its major contribution to resistance burden within the study population. The greatest resistance burden (Resistance Burden Score = 9164.6) was observed for *Staphylococcus pseudintermedius* isolated from skin specimens and tested against amoxicillin, followed by isolates from ear (4560.9) and urine (3880.6) specimens. *Staphylococcus pseudintermedius* isolated from skin specimens also ranked among the leading contributors for amoxicillin/clavulanate, cefpodoxime, cefovecin, cefalexin, and imipenem, demonstrating that this organism–specimen combination consistently contributed to resistance across multiple beta-lactam agents. *Escherichia coli* was the second largest contributor to the overall resistance burden, particularly among urine and other clinical specimens tested against amoxicillin and amoxicillin/clavulanate, reflecting the high frequency of resistant isolates within these combinations. *Proteus mirabilis* isolated from ear specimens also ranked highly for both amoxicillin and cefalexin, whereas urinary *Klebsiella* spp. remained among the leading contributors despite fewer observations because of their exceptionally high resistance prevalence.

To identify high-burden combinations within the diagnostic dataset beyond overall resistance prevalence, bacteria–specimen–antimicrobial combinations were ranked within each animal category using the Resistance Burden Score, which integrates both resistance prevalence and the absolute number of resistant isolates (Supplementary Figures S1–S7). Across all animal categories, resistance burden was concentrated within a relatively small number of combinations rather than being evenly distributed, indicating that a limited subset of pathogens and specimen types accounted for a disproportionate share of resistant isolates. However, the composition of these high-burden combinations differed markedly among animal categories, demonstrating substantial host-specific heterogeneity in the epidemiology of beta-lactam resistance. In companion animals and equine, the highest resistance burden was dominated by urinary isolates of *Escherichia coli*, reflecting both the frequency of isolation and consistently high resistance levels. In contrast, avian, food animal, reptile and amphibian, non-human primate, and zoo/wildlife/aquatic populations exhibited more diverse bacterial profiles, with multiple bacterial taxa contributing to the highest-ranked combinations. Reptiles and amphibians demonstrated the greatest heterogeneity, whereas non-human primates showed a distinct predominance of *Staphylococcus aureus* among the highest-burden combinations. Despite these differences in bacterial composition, amoxicillin, cefalexin, and amoxicillin/clavulanate consistently accounted for many of the highest resistance burden scores across animal categories, whereas imipenem contributed relatively few high-burden combinations. These findings demonstrate that the observed beta-lactam resistance burden is concentrated in a limited number of recurrent pathogens–specimen–antimicrobial combinations that vary substantially between animal populations, providing a framework for prioritizing surveillance, antimicrobial stewardship, and empirical treatment strategies according to host species rather than relying solely on overall resistance prevalence.

## 3. Discussion

The gradient in resistance observed across the seven beta-lactam agents appears to reflect patterns of veterinary antimicrobial use. The high prevalence of amoxicillin resistance (56.3%) was broadly consistent with previous reports of ampicillin and penicillin resistance in companion animals, including estimates of 40–52.7% in clinical *Escherichia coli* isolates and 56.9% in canine *Staphylococcus pseudintermedius* [7,20]. However, our estimate was higher than the 34.3% ampicillin resistance reported in canine urinary *E. coli* isolates from the northeastern United States [23], the approximately 20% resistance observed in the European ComPath urinary surveillance program [24], and the 21.1% resistance reported among commensal *E. coli* from US food animals [9]. These differences likely reflect the broader case mix in our study, which included multiple specimen types and bacterial taxa and was based on diagnostic laboratory submissions that are inherently enriched for recurrent and previously treated infections. In contrast, resistance to ceftazidime (11.2%) and imipenem (17.2%) remained comparatively low, consistent with their limited use in veterinary medicine and previous reports of high carbapenem susceptibility among companion-animal isolates [7,20]. Collectively, these findings suggest an inverse relationship between antimicrobial use intensity and resistance, supporting the importance of antimicrobial stewardship in preserving the efficacy of higher-tier beta-lactam agents. Beyond differences among antimicrobials, resistance patterns also varied substantially among animal categories, indicating that host-related factors contribute importantly to the epidemiology of beta-lactam resistance. No single animal category consistently exhibited the highest resistance across all agents; however, non-human primates demonstrated particularly high resistance to several beta-lactams, including cefalexin (69.1%) and cefovecin (66.6%), and had the highest adjusted odds of imipenem resistance. This finding is consistent with reports of beta-lactam-resistant and ESBL-producing Enterobacteriaceae in captive and anthropogenically exposed primate populations [25]. Food animals and reptiles also exhibited relatively high resistance to aminopenicillins and cefalexin, aligning with reports of increasing ampicillin resistance among commensal *Escherichia coli* from US food animals [9] and the high prevalence of multidrug-resistant *Salmonella* among pet reptiles [26]. In contrast, the comparatively low resistance observed among zoo, wildlife, and aquatic animals agrees with studies reporting generally low resistance among wildlife-associated *E. coli*, except in populations with substantial anthropogenic exposure [17]. Although companion animals exhibited intermediate resistance levels, they accounted for approximately 96% of all observations and therefore largely determined the overall resistance estimates, whereas findings for the less frequently represented animal categories should be interpreted more cautiously.

Stratification of the resistance burden by animal category further demonstrated that the combinations contributing most substantially to beta-lactam resistance differed across host populations. Although the overall burden was dominated by common pathogens such as *Staphylococcus pseudintermedius* and *Escherichia coli*, the highest-ranking bacteria–specimen–antimicrobial combinations varied among companion animals, equine, avian, food animals, reptiles and amphibians, non-human primates, and zoo, wildlife, and aquatic species. These findings indicate that resistance burden is influenced not only by bacterial prevalence but also by host-specific disease ecology, specimen submission patterns, and antimicrobial exposure. Consequently, stewardship interventions and surveillance priorities may be more effective when tailored to individual animal categories rather than relying solely on aggregate resistance estimates across the veterinary population. Although resistance differed among animal categories, the most pronounced heterogeneity was observed at the bacterial taxon level, which emerged as the factor showing the strongest association with beta-lactam resistance in the multivariable analyses. The very high aminopenicillin resistance observed in *Klebsiella* spp. (98.1% among urinary isolates) and *Enterobacter* spp. is consistent with previous reports from companion-animal Enterobacteriaceae in the United States [27]. For *Escherichia coli*, the observed amoxicillin resistance (56.3%) was higher than the 31.4% ampicillin resistance reported among canine urinary isolates [20] but broadly comparable with the 40–52.7% resistance reported in mixed clinical isolates from dogs and cats [7], likely reflecting the inclusion of multiple specimen types and bacterial populations in our study. Similarly, the high resistance observed in *Staphylococcus pseudintermedius* (approximately 84% to amoxicillin) greatly exceeded the 18.4–25.2% penicillin resistance and <11% ampicillin/amoxicillin–clavulanate resistance reported in the European ComPath surveillance program [28]. This discrepancy likely reflects differences in study populations, as our dataset was derived from diagnostic laboratory submissions that are enriched for chronic and previously treated infections, particularly skin and ear infections, whereas the ComPath program sampled animals with limited recent antimicrobial exposure. Recent studies have similarly documented substantial antimicrobial resistance among methicillin-resistant *S. pseudintermedius* recovered from companion animals, reinforcing the importance of resistant staphylococci in veterinary medicine and their potential One Health relevance [29]. In contrast, *Streptococcus* spp. remained comparatively susceptible across most beta-lactams, while *Enterococcus faecalis*, *Proteus mirabilis*, *Pseudomonas* spp., and *Acinetobacter* spp. exhibited distinct, agent-specific resistance profiles. Collectively, these findings demonstrate that bacterial identity is a major determinant of beta-lactam resistance and underscore the importance of species-level information when guiding empirical antimicrobial therapy and resistance surveillance.

Beyond host and bacterial differences, resistance is also clustered strongly by clinical specimen sources. Skin, wound, and ear specimens consistently exhibited the highest resistance prevalence (amoxicillin resistance of 72.7% in skin and 69.9% in wound specimens), whereas urine specimens generally showed the lowest resistance. Similar patterns have been reported in previous veterinary surveillance studies, where dermatological and soft-tissue specimens demonstrated substantially higher multidrug and methicillin resistance than urinary specimens [16]. The predominance of *Staphylococcus pseudintermedius* and other staphylococci in skin, wound, and ear infections, as documented in the ComPath surveillance program [28,30], likely contributes to these elevated resistance estimates. In contrast, the lower resistance observed in urine specimens is consistent with previous reports in which *Escherichia coli* predominated and ampicillin resistance remained comparatively low (approximately 20%) [24]. The high cefalexin resistance observed in anal–rectal specimens (77.2%) further reflects differences in the bacterial populations recovered from specific clinical sites. Collectively, these findings suggest that specimen source functions epidemiologically as a proxy for the underlying pathogen composition and clinical history of infection rather than acting as an independent determinant of resistance.

Overall, several findings were consistent with expected epidemiological patterns, whereas others were less anticipated. The comparatively high resistance to amoxicillin and cefalexin and the concentration of resistance among skin, wound, and ear isolates were broadly consistent with previous veterinary surveillance reports and with the predominance of staphylococci, particularly *Staphylococcus pseudintermedius*, in dermatological and soft-tissue infections. In contrast, the magnitude of amoxicillin resistance observed in *S. pseudintermedius* was higher than that reported in some previous surveillance studies, potentially reflecting differences in case mix, prior antimicrobial exposure, specimen composition, and the referral nature of diagnostic laboratory submissions. The absence of a significant temporal trend from 2018 to 2024 was also noteworthy given reports of increasing beta-lactam resistance in other animal populations. However, this finding should be interpreted cautiously because the present analysis used binary susceptibility classifications and therefore may not detect gradual shifts in MIC distributions that remain below clinical resistance breakpoints. Recent characterization of methicillin-resistant *S. pseudintermedius* from canine and feline clinical cases also demonstrates the substantial multidrug-resistance potential of this organism and supports continued companion-animal AMR surveillance [29].

After simultaneous adjustment and FDR correction, state, animal category, bacterial group, and clinical specimen group remained factors independently associated with beta-lactam resistance, indicating that the observed associations were not simply the result of confounding among correlated factors. The dominant influence of bacterial taxon is consistent with previous veterinary diagnostic-laboratory studies, in which bacterial species emerged as one of the strongest predictors of multidrug and methicillin resistance [7,16]. Likewise, the adjusted association with specimen source agrees with findings that clinical specimen type and healthcare setting significantly influence resistance patterns [16], while the persistent host effect is consistent with studies reporting animal-related factors, including breed, age, and isolation source, as factors associated with resistance [15]. Notably, isolates from Oklahoma exhibited consistently lower adjusted odds of resistance than those from Texas across all seven beta-lactam agents, suggesting that regional differences in resistance persist even after accounting for host, bacterial, and specimen characteristics. Similar state variation has been reported in previous US studies [7] and may reflect differences in antimicrobial use practices, case mix, referral patterns, or local bacterial populations between regions. Collectively, these findings demonstrate that beta-lactam resistance is shaped by multiple independent epidemiological factors rather than by any single host, bacterial, or state determinant. Because the dataset is very large, statistically significant associations with odds ratios close to 1.0 may have limited clinical importance; interpretation therefore emphasizes effect size and confidence intervals rather than *p*-values alone.

The absence of a consistent temporal pattern in beta-lactam resistance between 2018 and 2024 warrants cautious interpretation. Several studies have reported increasing beta-lactam resistance over time, including rising ampicillin, amoxicillin–clavulanate, and ceftriaxone resistance among commensal *Escherichia coli* from US food animals [9] and broader increases in resistance among food-animal pathogens globally [4]. However, our findings are consistent with reports of relative short-term stability in resistance patterns among companion-animal diagnostic laboratory datasets [14]. Importantly, Osman et al. [8] demonstrated that increasing beta-lactam minimum inhibitory concentrations (MICs) may occur without corresponding increases in resistance prevalence defined by clinical breakpoints. Because our analysis was based on binary susceptibility outcomes rather than MIC distributions, gradual shifts in susceptibility below established resistance thresholds may not have been detected. The rarefaction analysis also materially changed the interpretation of bacterial richness. Although companion animals had the greatest unstandardized richness because they dominated the diagnostic submissions, this pattern did not persist after all animal category–antimicrobial groups were standardized to the same number of observations. Zoo/wildlife/aquatic and reptile/amphibian groups showed the greatest rarefied richness, demonstrating the importance of controlling for sampling effort before comparing taxonomic richness across host populations. Rarefied Shannon, Simpson, and Pielou indices further showed that diversity and evenness varied across host categories independently of submission volume. These findings support the use of ecological metrics as complementary descriptive tools in veterinary AMR surveillance when accompanied by appropriate sample-size standardization. The resistance burden score, defined as resistance prevalence weighted by the number of resistant observations, extends conventional prevalence estimates by incorporating both the frequency and magnitude of resistance within the study population. Conceptually, this approach aligns with broader prioritization frameworks that weigh resistance according to overall impact [1,2,31], while operating at the granular bacteria–specimen–antimicrobial level using routinely collected veterinary diagnostic laboratory data.

The burden ranking demonstrated that resistance was concentrated within a relatively small number of combinations, particularly *Staphylococcus pseudintermedius* isolated from skin specimens and tested against amoxicillin, which contributed to the single largest resistance burden and accounted for nine of the top twenty ranked combinations. In contrast, combinations such as urinary *Klebsiella* spp. exhibited extremely high resistance prevalence but contributed less to the overall burden because of their lower frequency. These findings illustrate the value of integrating prevalence with resistant isolate counts, as reliance on prevalence alone may overemphasize rare but highly resistant combinations while underestimating common, moderately resistant ones. Consequently, the resistance burden score provides a simple, transparent, and locally adaptable framework for prioritizing surveillance targets and antimicrobial stewardship interventions in veterinary medicine. These findings have important One Health implications. Several of the organisms contributing most to the resistance burden, including beta-lactam-resistant *Escherichia coli* and staphylococci, are recognized human and animal health priorities, and resistant bacteria and resistance determinants can be shared between pets and their owners [13,19]. Moreover, companion animals remain largely absent from formal national AMR surveillance programs despite their close and frequent contact with humans, a recognized gap that veterinary diagnostic laboratory data can help address [14]. The high resistance observed for aminopenicillins and the concentration of resistance burden within a limited number of organism–specimen combinations support stewardship strategies that discourage empirical aminopenicillin use for recurrent skin and soft-tissue infections and reinforce culture-guided therapy. The comparatively low laboratory-reported resistance to imipenem and ceftazidime is encouraging but should be interpreted cautiously because these agents may be tested selectively, organism composition differs across panels, and diagnostic submissions are not population-based. Their importance to human medicine nevertheless supports continued One Health surveillance [10]. Several factors may contribute to the absence of a detectable temporal trend, including relatively stable resistance patterns within the dominant diagnostic populations and the limited sensitivity of binary susceptibility classifications to incremental changes in MICs. However, because antimicrobial-use, treatment-history, and quantitative MIC data were unavailable, the underlying reasons for the observed temporal pattern could not be determined directly.

This study makes several contributions to veterinary antimicrobial resistance surveillance. First, by integrating a large diagnostic laboratory dataset across multiple animal populations, bacterial taxa, clinical specimen sources, and beta-lactam antimicrobials, it provides a broader assessment of phenotypic beta-lactam resistance than studies restricted to individual hosts, bacterial species, or specimen types. Second, the simultaneous evaluation of host-, bacterial-, specimen-, and state-level factors demonstrates the substantial heterogeneity underlying resistance patterns and emphasizes the importance of considering these factors when interpreting diagnostic laboratory surveillance data. Third, the resistance burden analysis complements prevalence estimates by identifying organism–specimen–antimicrobial combinations that contribute disproportionately to the observed resistance burden, thereby providing a potential framework for prioritizing surveillance and stewardship efforts. From a clinical perspective, the marked variation in resistance across bacterial taxa and specimen sources reinforces the importance of culture and antimicrobial susceptibility testing to support antimicrobial selection rather than relying solely on empirical treatment. From a One Health perspective, continued surveillance of beta-lactam resistance in animal populations is particularly important for agents that are also critically important in human medicine and may help identify emerging resistance patterns requiring targeted investigation.

This study has several limitations. First, the retrospective design relied on routinely generated veterinary diagnostic laboratory data and was therefore subject to potential differences in specimen submission, antimicrobial testing, and laboratory practices. Because isolates originated from diagnostic submissions rather than population-based sampling, resistance estimates should not be interpreted as population-level prevalence estimates for all animals in Texas and Oklahoma. Second, companion animals constituted most of the dataset, resulting in unequal representation across animal categories. We addressed the specific effect of unequal sampling on bacterial richness by performing individual-based rarefaction, but rarefaction does not remove other forms of selection inherent to diagnostic submissions. Third, the geographic scope was restricted to Texas and Oklahoma and may not be generalizable to other regions. Fourth, analyses were based on categorical susceptibility interpretations rather than quantitative MIC distributions, so subtle shifts below established resistance breakpoints could not be assessed. Fifth, antimicrobial analyses were restricted to agents with sufficient testing coverage, which may underrepresent species- or panel-specific agents, particularly in food animals. Accession Number identified a diagnostic submission/case rather than a unique animal. Consequently, repeated submissions from the same animal could not be identified reliably, and patient- or clinic-level clustering could not be incorporated; residual dependence from repeated submissions by the same animal or submitting clinic therefore cannot be excluded. Each antimicrobial-specific model nevertheless contained at most one binary susceptibility result per isolate for that antimicrobial. Finally, susceptibility categories were analyzed as reported by the source laboratories, and raw MIC values, testing-platform metadata, and the exact breakpoint rule applied to every organism–drug result were unavailable for retrospective adjudication. To reduce overinterpretation, organism–antimicrobial heatmap cells with fewer than 30 interpretable observations were suppressed, and expected resistant or breakpoint-sensitive organism–drug combinations were interpreted cautiously. Future studies should incorporate prospectively collected and geographically broader data, unique patient and clinic identifiers, more balanced animal representation, antimicrobial-use and treatment-history information, and quantitative MIC or molecular resistance data.

Collectively, these findings demonstrate the value of integrating routine veterinary diagnostic laboratory data and burden-based prioritization approaches into regional AMR surveillance frameworks to support evidence-based stewardship and empirical treatment guidelines across the human–animal interface.

## 4. Materials and Methods

### 4.1. Data Source and Study Population

This retrospective study assessed antimicrobial susceptibility testing (AST) data from bacterial isolates submitted to commercial veterinary diagnostic laboratories in Texas and Oklahoma, USA, from 2018 to 2024. The study period was defined by the temporal coverage of the dataset provided by the diagnostic laboratory, which included records available from 2018 through 2024. The initial dataset included 166,686 isolates of bacteria from 80 animal species, 135 bacterial taxa, 108 clinical specimen types, and susceptibility results for 19 beta-lactam antimicrobials. The study was meant to evaluate epidemiological patterns of beta-lactam resistance in animal populations, bacterial taxa, clinical specimen sources, and states. The dataset included isolates originating from 80 animal species, which were subsequently consolidated into seven analytical animal categories to facilitate comparison: companion animals, avian species, equine, food animals, non-human primates, reptiles and amphibians, and zoo/wildlife/aquatic animals (Supplementary Table S1). Individual-level clinical information indicating whether animals were healthy or diseased at the time of specimen collection was not available in the dataset. Therefore, health status could not be directly evaluated. Because the data originated from specimens submitted to veterinary diagnostic laboratories for bacterial isolation and antimicrobial susceptibility testing, the study population represents diagnostic submissions rather than a randomly sampled population of healthy and diseased animals.

### 4.2. Data Cleaning, Variable Grouping, and Antimicrobial Selection

Data cleaning was performed prior to analysis to standardize animal species, bacterial taxonomy, clinical specimen descriptions, state identifiers and antimicrobial susceptibility results. Records removed from the analysis were those with missing or uninterpretable antimicrobial susceptibility values, unclassified animal species, and records originating outside Texas and Oklahoma. For the primary analysis, susceptibility outcomes were restricted to interpretable binary categories (susceptible or resistant); intermediate, not-tested, missing, and unknown results were excluded. To improve interpretability and reduce sparsity, animal species were grouped into seven analytical animal categories (Supplementary Table S1). Similarly, bacterial taxa were consolidated into 26 analytical bacterial groups, consisting of the 25 most frequently recognized taxa and one “Other bacterial taxa” category (Supplementary Table S3). Clinical specimen types were similarly categorized into 11 analytical specimen categories, including the 10 most often submitted specimen types and one “Other clinical specimens” category (Supplementary Table S4). Nineteen beta-lactam antimicrobials were first found in the raw dataset. The proportion of isolates with interpretable susceptibility values after data cleaning was calculated to determine the completeness of testing for each antimicrobial. Antimicrobials were ranked by the proportion of isolates with interpretable binary susceptibility results. The seven agents with the highest coverage were retained; the seventh-ranked agent, ceftazidime, had 48.8% coverage, making 48.8% a data-derived cutoff rather than a prespecified biological or clinical threshold. Agents below this cutoff were excluded from the primary cross-population analysis because of greater sparsity and likely panel-specific testing, which could compromise comparability across animal categories. This approach led to the retention of 7 beta-lactam antimicrobials: amoxicillin, amoxicillin/clavulanate, cefalexin, cefovecin, cefpodoxime, ceftazidime and imipenem. The summary of the antimicrobial selection method and completeness of testing is shown in Supplementary Table S2. For descriptive statistics, overall resistance prevalence was calculated separately for each retained beta-lactam antimicrobial as susceptibility categories were analyzed as supplied by the diagnostic laboratories. Because raw MICs and complete organism-specific breakpoint metadata were not available in the study dataset, the analysis could not retrospectively reclassify all organism–antimicrobial combinations according to a single CLSI or EUCAST standard; expected resistant phenotypes were therefore treated cautiously in interpretation.

Resistance prevalence (%) = (Number of resistant observations/Total susceptibility observations) × 100,(1)

Wilson 95% confidence intervals were calculated for each prevalence estimate.

### 4.3. Statistical Analysis

Descriptive resistance summaries were prepared by animal category, bacterial group, and clinical specimen group. Heatmaps were used to visualize resistance prevalence across strata. For Figure 3, bacterial taxon–antimicrobial cells with fewer than 30 interpretable binary susceptibility observations were not estimated and were displayed as NA to reduce instability from sparse testing; *Enterococcus*–cephalosporin combinations were displayed as NR because the expected cephalosporin-resistant phenotype was not interpreted as acquired resistance. Detailed bacterial taxa summaries by antimicrobial are provided in Supplementary Tables S5–S11. Univariable logistic regression models were fitted separately for each retained beta-lactam antimicrobial to assess unadjusted associations between resistance and year, state, animal category, bacterial group, and clinical specimen group. Multivariable logistic regression models were then fitted separately for each antimicrobial with the same prespecified covariates entered simultaneously. Year was modeled categorically with 2018 as the reference year, and *p*-values were adjusted using the Benjamini–Hochberg FDR approach. Each antimicrobial-specific model contained at most one binary susceptibility result per isolate for that antimicrobial. Accession Number represented a diagnostic submission/case rather than a unique animal; because consistently usable patient- and clinic-level identifiers were unavailable, repeated submissions from the same animal or clinic could not be identified and clustering at those levels could not be incorporated.

Bacterial diversity was evaluated separately for each animal category and retained beta-lactam antimicrobial using the original bacterial taxa represented in the binary susceptibility dataset. Because submission volume differed markedly among animal categories, individual-based rarefaction was performed before between-category interpretation. Each animal category–antimicrobial group was standardized to 164 interpretable binary susceptibility observations, corresponding to the smallest available group size, and 1000 random subsamples without replacement were generated using a fixed random seed for reproducibility. For each subsample, bacterial richness, Shannon diversity, Simpson diversity, and Pielou’s evenness were calculated; Supplementary Table S14 reports the mean standardized estimates and 95% empirical intervals for richness and Shannon diversity. These metrics were used descriptively to characterize the taxonomic composition and evenness of organisms represented in diagnostic submissions and were not intended to infer microbiome diversity or population-level bacterial communities. To identify bacteria–specimen–antimicrobial combinations contributing most to the observed beta-lactam resistance burden, the Resistance Burden Score was calculated as (Resistance prevalence [%]/100) × Number of resistant observations. Only combinations with at least five susceptibility observations were included. The score is a descriptive, frequency-weighted prioritization metric for this dataset and is not a validated estimate of population-level clinical burden. All analyses were conducted using R version 4.5.2 (R Foundation for Statistical Computing, Vienna, Austria) through a reproducible R Markdown workflow. 

## 5. Conclusions

This study provides one of the most comprehensive epidemiological assessments of beta-lactam resistance among veterinary bacterial isolates in the southern United States. Beta-lactam resistance varied substantially according to bacterial taxon, animal category, clinical specimen, and state, with no consistent adjusted year-specific differences detected across 2018–2024. Resistance was concentrated within a limited number of organism-specimen-antimicrobial combinations. Within the study dataset, *Staphylococcus pseudintermedius*, particularly isolates from skin specimens, contributed disproportionately to the observed beta-lactam resistance burden. Because the Resistance Burden Score incorporates both resistance prevalence and the frequency of resistant observations, these rankings reflect resistance burden within the diagnostic dataset and should not be interpreted as population-level estimates of clinical disease burden. These findings demonstrate the value of large veterinary diagnostic laboratory datasets for strengthening One Health surveillance and provide an evidence base for targeted stewardship interventions, surveillance prioritization, and evidence-based empirical therapy in veterinary medicine.

## Figures and Tables

**Table 1:** Study population and final analytical dataset characteristics.

Characteristic	Value
Study period	2018–2024
Total bacterial isolates	166,686
Susceptibility observations	789,871
Animal species represented	80
Analytical animal categories	7
Bacterial taxa identified	135
Analytical bacterial groups	26
Clinical specimen types	108
Analytical clinical specimen groups	11
States represented	2 (Texas and Oklahoma)
Beta-lactam antimicrobials evaluated	19
Retained beta-lactam antimicrobials	7

**Table 2:** Overall beta-lactam resistance prevalence by antimicrobial.

Antimicrobial	Resistant (n)	Susceptible (n)	Total Isolates (n)	Resistance % (95% CI)
Amoxicillin	67,709	52,662	120,371	56.25 (55.97–56.53)
Cefalexin	48,083	63,631	111,714	43.04 (42.75–43.33)
Amoxicillin/Clavulanate	50,082	81,668	131,750	38.01 (37.75–38.28)
Cefovecin	33,344	81,240	114,584	29.10 (28.84–29.36)
Cefpodoxime	33,862	83,549	117,411	28.84 (28.58–29.10)
Imipenem	19,419	93,635	113,054	17.18 (16.96–17.40)
Ceftazidime	9052	71,935	80,987	11.18 (10.96–11.40)

CI, confidence interval. Resistance prevalence was calculated as the number of resistant observations divided by the total number of susceptibility observations; 95% CIs are Wilson intervals.

## Data Availability

The data presented in this study are available on request from the corresponding author. The data are not publicly available owing to confidentiality agreements with the contributing commercial veterinary diagnostic laboratories.

## Supplementary Materials

The following supporting information can be downloaded at: https://www.mdpi.com/article/10.3390/antibiotics15090836/s1, Table S1: Classification of original animal species into analytical animal categories. Table S2: Completeness and selection of beta-lactam antibiotics. Binary coverage represents the percentage of study-population records with an interpretable susceptible/resistant result, after excluding intermediate, not-tested, and missing/unknown codes. The seven antimicrobials with the highest binary coverage were retained for the primary analysis; 48.8% was the coverage of the seventh-ranked retained agent (ceftazidime) and therefore represents a data-derived cutoff rather than a prespecified clinical threshold. Agents below this cutoff may reflect panel-specific testing and remain clinically relevant in particular animal populations. Table S3: Bacterial taxon grouping. Non-taxonomic descriptors (‘Normal flora’, ‘Gram-positive cocci’) were removed prior to grouping. The 25 most frequently isolated bacterial taxa were retained individually; all remaining taxa were collapsed into the category ‘Other bacterial taxa’. Table S4: Clinical specimen grouping. The 10 most common clinical specimen types (excluding the generic ‘Other’ code) were retained individually; all remaining specimen types were collapsed into the category ‘Other clinical specimens’. Table S5: Amoxicillin resistance summarised by bacterial taxon group and clinical specimen group (combinations with fewer than 5 Total Isolates (n) are omitted for stability). Table S6: Amoxicillin/Clavulanate resistance summarised by bacterial taxon group and clinical specimen group (combinations with fewer than 5 Total Isolates (n) are omitted for stability). Table S7: Cefalexin resistance summarised by bacterial taxon group and clinical specimen group (combinations with fewer than 5 Total Isolates (n) are omitted for stability). Table S8: Cefovecin resistance summarised by bacterial taxon group and clinical specimen group (combinations with fewer than 5 Total Isolates (n) are omitted for stability). Table S9: Cefpodoxime resistance summarised by bacterial taxon group and clinical specimen group (combinations with fewer than 5 Total Isolates (n) are omitted for stability). Table S10: Ceftazidime resistance summarised by bacterial taxon group and clinical specimen group (combinations with fewer than 5 Total Isolates (n) are omitted for stability). Table S11: Imipenem resistance summarised by bacterial taxon group and clinical specimen group (combinations with fewer than 5 Total Isolates (n) are omitted for stability). Table S12: Univariable logistic regression of beta-lactam resistance on Year, Region, Animal Category, Bacteria Group, and Sample Group, fitted separately for each retained antibiotic. Odds ratios and 95% confidence intervals were derived using the Wald (asymptotic normal) approximation. Table S13: Multivariable logistic regression of beta-lactam resistance, fitted separately for each retained antibiotic, adjusting simultaneously for Year, Region, Animal Category, Bacteria Group, and Sample Group. Reference categories: Year = 2018, Region = TX, Animal Category = Companion Animals, Bacteria Group = *Escherichia coli*, Sample Group = Urine. Benjamini–Hochberg FDR correction was applied within each antibiotic-specific model. Table S14: Sample-size-standardized bacterial diversity by Animal Category × Antimicrobial. Each group was rarefied to n = 164 interpretable binary susceptibility observations using 1000 random subsamples without replacement. Diversity was calculated from the original bacterial taxa represented in the binary susceptibility dataset. Table S15: Top 20 Bacteria × Specimen × Antibiotic combinations ranked by Resistance Burden Score ([resistance prevalence %/100] × number of resistant observations), among combinations with at least 5 Total Isolates (n). Figure S1: Top 20 bacterial group–clinical specimen group–antimicrobial combinations ranked by Resistance Burden Score among companion animals. Figure S2: Top 20 bacterial group–clinical specimen group–antimicrobial combinations ranked by Resistance Burden Score among equine isolates. Figure S3: Top 20 bacterial group–clinical specimen group–antimicrobial combinations ranked by Resistance Burden Score among avian isolates. Figure S4: Top 20 bacterial group–clinical specimen group–antimicrobial combinations ranked by Resistance Burden Score among food-animal isolates. Figure S5: Top 20 bacterial group–clinical specimen group–antimicrobial combinations ranked by Resistance Burden Score among reptiles and amphibians. Figure S6: Top 20 bacterial group–clinical specimen group–antimicrobial combinations ranked by Resistance Burden Score among non-human primates. Figure S7: Top 20 bacterial group–clinical specimen group–antimicrobial combinations ranked by Resistance Burden Score among zoo, wildlife, and aquatic species.

## Abbreviations

The following abbreviations are used in this manuscript:

AMR	Antimicrobial resistance
aOR	Adjusted odds ratio
APHIS	Animal and Plant Health Inspection Service
AST	Antimicrobial susceptibility testing
CI	Confidence interval
ESBL	Extended-spectrum beta-lactamase
FDR	False discovery rate
MIC	Minimum inhibitory concentration
NARMS	National Antimicrobial Resistance Monitoring System
OR	Odds ratio
USDA	United States Department of Agriculture

## References

[1] Murray C.J.L., Ikuta K.S., Sharara F., Swetschinski L., Robles Aguilar G., Gray A., Han C., Bisignano C., Rao P., Wool E. (2022). Global burden of bacterial antimicrobial resistance in 2019: A systematic analysis. Lancet.

[2] Naghavi M., Vollset S.E., Ikuta K.S., Swetschinski L.R., Gray A.P., Wool E.E., Aguilar G.R., Mestrovic T., Smith G., Han C. (2024). Global burden of bacterial antimicrobial resistance 1990–2021: A systematic analysis with forecasts to 2050. Lancet.

[3] McEwen S.A., Collignon P.J. (2018). Antimicrobial resistance: A One Health perspective. Microbiol. Spectr..

[4] Van Boeckel T.P., Pires J., Silvester R., Zhao C., Song J., Criscuolo N.G., Gilbert M., Bonhoeffer S., Laxminarayan R. (2019). Global trends in antimicrobial resistance in animals in low- and middle-income countries. Science.

[5] Goggs R., Menard J.M., Altier C., Cummings K.J., Jacob M.E., Lalonde-Paul D.F., Papich M.G., Norman K.N., Fajt V.R., Scott H.M. (2021). Patterns of antimicrobial drug use in veterinary primary care and specialty practice: A 6-year multi-institution study. J. Vet. Intern. Med..

[6] Murphy C.P., Reid-Smith R.J., Boerlin P., Weese J.S., Prescott J.F., Janecko N., McEwen S.A. (2012). Out-patient antimicrobial drug use in dogs and cats for new disease events from community companion animal practices in Ontario. Can. Vet. J..

[7] Thungrat K., Price S.B., Carpenter D.M., Boothe D.M. (2015). Antimicrobial susceptibility patterns of clinical Escherichia coli isolates from dogs and cats in the United States: January 2008 through January 2013. Vet. Microbiol..

[8] Osman M., Albarracin B., Altier C., Gröhn Y.T., Cazer C. (2022). Antimicrobial resistance trends among canine Escherichia coli isolated at a New York veterinary diagnostic laboratory between 2007 and 2020. Prev. Vet. Med..

[9] Sodagari H.R., Varga C. (2023). Evaluating antimicrobial resistance trends in commensal Escherichia coli isolated from cecal samples of swine at slaughter in the United States, 2013–2019. Microorganisms.

[10] World Health Organization (2024). WHO Medically Important Antimicrobials List for Human Medicine.

[11] Hossain H., Chowdhury M.S.R., Islam S., Ahmad T., Sayeed S.S.B., Rahman M.M., Rahman M.M. (2026). Extended spectrum beta lactamase producing multidrug resistant Enterobacteriaceae in livestock-derived foods: Global trends, threats, impact and mitigation. Food Saf. Risk.

[12] Hossain H., Chowdhury M.S.R., Rahman M.N., Khan S.S., Ahmad T., Akter S., Nitu A.S., Brishty K.A., Pory F.S., Dipsana K.C. (2025). Antimicrobial resistance apocalypse and pan-drug-resistant superbug challenges: Current status, impact and mitigation strategies. Pak. Vet. J..

[13] Salgado-Caxito M., Benavides J.A., Adell A.D., Paes A.C., Moreno-Switt A.I. (2021). Global prevalence and molecular characterization of extended-spectrum β-lactamase producing-Escherichia coli in dogs and cats—A scoping review and meta-analysis. One Health.

[14] Sobkowich K.E., Weese J.S., Poljak Z., Plum A., Szlosek D., Bernardo T.M. (2023). Epidemiology of companion animal AMR in the United States of America: Filling a gap in the One Health approach. Front. Public Health.

[15] Ekakoro J.E., Hendrix G.K., Guptill L.F., Ruple A. (2022). Antimicrobial susceptibility and risk factors for resistance among Escherichia coli isolated from canine specimens submitted to a diagnostic laboratory in Indiana, 2010–2019. PLoS ONE.

[16] Lord J., Millis N., Jones R.D., Johnson B., Kania S.A., Odoi A. (2023). An epidemiological study of the predictors of multidrug resistance and methicillin resistance among Staphylococcus spp. isolated from canine specimens submitted to a diagnostic laboratory in Tennessee, USA. PeerJ.

[17] Awosile B., Fritzler J., Levent G., Rahman M.K., Ajulo S., Daniel I., Tasnim Y., Sarkar S. (2023). Genomic characterization of fecal Escherichia coli isolates with reduced susceptibility to beta-lactam antimicrobials from wild hogs and coyotes. Pathogens.

[18] Lord J., Carter C., Smith J., Locke S., Phillips E., Odoi A. (2022). Antimicrobial resistance among Streptococcus equi subspecies zooepidemicus and Rhodococcus equi isolated from equine specimens submitted to a diagnostic laboratory in Kentucky, USA. PeerJ.

[19] Weese J.S., Van Duijkeren E. (2010). Methicillin-resistant Staphylococcus aureus and Staphylococcus pseudintermedius in veterinary medicine. Vet. Microbiol..

[20] Yudhanto S., Hung C.C., Maddox C.W., Varga C. (2022). Antimicrobial resistance in bacteria isolated from canine urine samples submitted to a veterinary diagnostic laboratory, Illinois, United States. Front. Vet. Sci..

[21] Clinical and Laboratory Standards Institute (2024). Performance Standards for Antimicrobial Disk and Dilution Susceptibility Tests for Bacteria Isolated from Animals.

[22] Clinical and Laboratory Standards Institute (2024). Understanding Susceptibility Test Data as a Component of Antimicrobial Stewardship in Veterinary Settings.

[23] Cummings K.J., Aprea V.A., Altier C. (2015). Antimicrobial resistance trends among canine Escherichia coli isolates obtained from clinical samples in the northeastern USA, 2004–2011. Can. Vet. J..

[24] Moyaert H., Morrissey I., De Jong A., El Garch F., Klein U., Ludwig C., Thiry J., Youala M. (2017). Antimicrobial susceptibility monitoring of bacterial pathogens isolated from urinary tract infections in dogs and cats across Europe: ComPath results. Microb. Drug Resist..

[25] Tanga C.T.F., Makouloutou-Nzassi P., Mbehang Nguema P.P., Düx A., Lendzele Sevidzem S., Mavoungou J.F., Leendertz F.H., Mintsa-Nguema R. (2024). Antimicrobial resistance in African great apes. Antibiotics.

[26] Marin C., Lorenzo-Rebenaque L., Laso O., Villora-Gonzalez J., Vega S. (2021). Pet reptiles: A potential source of transmission of multidrug-resistant Salmonella. Front. Vet. Sci..

[27] Rahman M.K., Williams R.B., Ajulo S., Levent G., Loneragan G.H., Awosile B. (2024). Predictive modeling of phenotypic antimicrobial susceptibility of selected beta-lactam antimicrobials from beta-lactamase resistance genes. Antibiotics.

[28] Ludwig C., De Jong A., Moyaert H., El Garch F., Janes R., Klein U., Morrissey I., Thiry J., Youala M. (2016). Antimicrobial susceptibility monitoring of dermatological bacterial pathogens isolated from diseased dogs and cats across Europe (ComPath results). J. Appl. Microbiol..

[29] Dewulf S., Boyen F., Paepe D., Clercx C., Tilman N., Dewulf J., Boland C. (2025). Antimicrobial resistance characterization of methicillin-resistant Staphylococcus aureus and Staphylococcus pseudintermedius isolates from clinical cases in dogs and cats in Belgium. Antibiotics.

[30] De Jong A., Youala M., El Garch F., Simjee S., Rose M., Morrissey I., Moyaert H. (2020). Antimicrobial susceptibility monitoring of canine and feline skin and ear pathogens isolated from European veterinary clinics: Results of the ComPath Surveillance programme. Vet. Dermatol..

[31] Sati H., Carrara E., Savoldi A., Hansen P., Garlasco J., Campagnaro E., Boccia S., Castillo-Polo J.A., Magrini E., Garcia-Vello P. (2025). The WHO Bacterial Priority Pathogens List 2024: A prioritisation study to guide research, development, and public health strategies against antimicrobial resistance. Lancet Infect. Dis..

