# Supplementary material for: Phenotypic Resistance to Beta-Lactam Antimicrobials in Bacterial Isolates from Different Animal Populations in Texas and Oklahoma, 2018–2024: A Retrospective Study
Source: Antibiotics (Basel). 2026 Aug 28;15(9):836. doi: 10.3390/antibiotics15090836 (PMC13603162; doi:10.3390/antibiotics15090836)
Supplement: Supplementary file 1 [file antibiotics-15-00836-s001.zip › antibiotics-4531773-supplementary.pdf]

Phenotypic Resistance to Beta-lactam Antimicrobials in Bacterial Isolates from Different Animal Populations

Supplementary Tables

Table S1. Classification of original animal species into analytical animal categories.

Original animal species	Analytical animal category
Cat	Companion Animals
Chinchilla	Companion Animals
Dog	Companion Animals
Ferret	Companion Animals
Gerbil	Companion Animals
Guinea pig	Companion Animals
Hamster	Companion Animals
Hedgehog	Companion Animals
Mouse	Companion Animals
Rabbit	Companion Animals
Rat	Companion Animals
Rodent	Companion Animals
African grey parrot	Avian
Amazon parrot	Avian
Bald eagle	Avian
Bird	Avian
Caique (parrot)	Avian
Canary	Avian
Canary finch	Avian
Cockatiel	Avian
Cockatoo	Avian
Conure	Avian
Dove	Avian
Duck	Avian
Eclectus (parrot)	Avian
Emu	Avian
Falcon	Avian
Fowl	Avian
Hawk	Avian
Lory (parrot)	Avian

Macaw	Avian
Meyer's parrot	Avian
Ostrich	Avian
Owl	Avian
Parakeet	Avian
Pheasant	Avian
Pigeon	Avian
Quaker parakeet	Avian
Ringneck parakeet	Avian
Toucan	Avian
Turkey	Avian
Horse	Equine
Alpaca	Food Animals
Cow	Food Animals
Deer	Food Animals
Goat	Food Animals
Hoofstock	Food Animals
Pig	Food Animals
Sheep	Food Animals
Cynomolgus monkey	Non-human Primates
Monkey	Non-human Primates
Rhesus monkey	Non-human Primates
Alligator	Reptiles & Amphibians
Amphibian	Reptiles & Amphibians
Bearded dragon	Reptiles & Amphibians
Boa (snake)	Reptiles & Amphibians
Box turtle	Reptiles & Amphibians
Corn snake	Reptiles & Amphibians
Desert tortoise	Reptiles & Amphibians
Iguana	Reptiles & Amphibians
Land tortoise	Reptiles & Amphibians
Leopard tortoise	Reptiles & Amphibians
Monitor lizard	Reptiles & Amphibians
Python (snake)	Reptiles & Amphibians
Red-eared slider (turtle)	Reptiles & Amphibians
Reptile	Reptiles & Amphibians
Texas tortoise	Reptiles & Amphibians
Water dragon	Reptiles & Amphibians

Asian elephant	Zoo/Wildlife/Aquatic
Bear	Zoo/Wildlife/Aquatic
Dolphin	Zoo/Wildlife/Aquatic
Elephant	Zoo/Wildlife/Aquatic
Fish	Zoo/Wildlife/Aquatic
Ray	Zoo/Wildlife/Aquatic
Sea lion	Zoo/Wildlife/Aquatic
Shark	Zoo/Wildlife/Aquatic
Zoo mammal	Zoo/Wildlife/Aquatic

Original animal species recorded in the source laboratory dataset were grouped into seven analytical animal categories to permit adequately powered stratified and multivariable analyses. Companion Animals comprises domestic pet species (dogs, cats, small mammals); Avian comprises domestic, companion, and wild bird species; Food Animals comprises production and agricultural livestock species; Reptiles & Amphibians comprises captive and wild herpetological species; Zoo/Wildlife/Aquatic comprises zoological collection, wildlife rehabilitation, and aquatic/marine species. Equine and Non-human Primates were retained as discrete categories given their distinct clinical and epidemiological contexts. This classification scheme was applied consistently across all analyses presented in the main manuscript and supplementary material.

Table S2. Completeness and selection of beta-lactam antibiotics. Binary coverage represents the percentage of study-population records with an interpretable susceptible/resistant result, after excluding intermediate, not-tested, and missing/unknown codes. The seven antimicrobials with the highest binary coverage were retained for the primary analysis; 48.8% was the coverage of the seventh-ranked retained agent (ceftazidime) and therefore represents a data-derived cutoff rather than a prespecified clinical threshold. Agents below this cutoff may reflect panel-specific testing and remain clinically relevant in particular animal populations.

Antibiotic	Total Isolates (n)	Isolates (%)	Included in primary analysis
Amoxicillin/Clavulanate	132340	79.39	Yes
Amoxicillin	120908	72.54	Yes
Cefpodoxime	117934	70.75	Yes
Cefovecin	115132	69.07	Yes
Imipenem	113550	68.12	Yes
Cefalexin	112231	67.33	Yes
Ceftazidime	81429	48.85	Yes
Ceftiofur	58202	34.92	No
Penicillin	54278	32.56	No
Cefotaxime	50991	30.59	No
Piperacillin	1591	0.95	No
Cefazolin	245	0.15	No
Ampicillin	3	0	No
Penicillin G	0	0	No
Ticarcillin	0	0	No
Cefalothin	0	0	No
Cefoxitin	0	0	No
Ticarcillin/Clavulanic Acid	0	0	No
Piperacillin/Tazobactam	0	0	No

Table S3. Bacterial taxon grouping. Non-taxonomic descriptors ('Normal flora', 'Gram-positive cocci') were removed prior to grouping. The 25 most frequently isolated bacterial taxa were retained individually; all remaining taxa were collapsed into the category 'Other bacterial taxa'.

**Bacteria**	**Analytical group**
Escherichia coli	*Escherichia coli*
Staphylococcus pseudintermedius	*Staphylococcus pseudintermedius*
Proteus mirabilis	*Proteus mirabilis*
Staphylococcus schleiferi	*Staphylococcus schleiferi*
Pseudomonas spp.	*Pseudomonas spp.*
Klebsiella spp.	*Klebsiella spp.*
Enterococcus faecalis	*Enterococcus faecalis*
Enterobacter spp.	*Enterobacter spp.*
Staphylococcus aureus	*Staphylococcus aureus*
Acinetobacter spp.	*Acinetobacter spp.*
Pantoea spp.	*Pantoea spp.*
Streptococcus spp.	*Streptococcus spp.*
Staphylococcus spp.	*Staphylococcus spp.*
Staphylococcus felis	*Staphylococcus felis*
Citrobacter spp.	*Citrobacter spp.*
Enterococcus faecium	*Enterococcus faecium*
Staphylococcus epidermidis	*Staphylococcus epidermidis*
Enterococcus spp.	*Enterococcus spp.*
Serratia spp.	*Serratia spp.*
Leclercia adecarboxylata	*Leclercia adecarboxylata*
Staphylococcus intermedius	*Staphylococcus intermedius*
Providencia spp.	*Providencia spp.*
Staphylococcus sciuri	*Staphylococcus sciuri*
Moraxella spp.	*Moraxella spp.*
Staphylococcus xylosus	*Staphylococcus xylosus*
Streptococcus zooepidemicus	Other bacterial taxa
Pasteurella spp.	Other bacterial taxa
Staphylococcus simulans	Other bacterial taxa
Staphylococcus chromogenes	Other bacterial taxa
Morganella morganii	Other bacterial taxa
Escherichia vulneris	Other bacterial taxa
Aeromonas spp.	Other bacterial taxa
Achromobacter spp.	Other bacterial taxa
Salmonella spp.	Other bacterial taxa
Psychrobacter	Other bacterial taxa
Escherichia hermannii	Other bacterial taxa
Bordetella spp.	Other bacterial taxa
Burkholderia spp.	Other bacterial taxa
Alcaligenes spp.	Other bacterial taxa
Corynebacterium spp.	Other bacterial taxa
Pluralibacter gergoviae	Other bacterial taxa
Raoultella ornithinolytica	Other bacterial taxa
Streptococcus equi	Other bacterial taxa
Brevundimonas spp.	Other bacterial taxa
Actinobacillus	Other bacterial taxa
Kluyvera spp.	Other bacterial taxa
Ochrobactrum spp.	Other bacterial taxa
Macrococcus canis	Other bacterial taxa

Plesiomonas shigelloides	Other bacterial taxa
Hafnia alvei	Other bacterial taxa
Lelliottia amnigena	Other bacterial taxa
Cronobacter spp.	Other bacterial taxa
Delftia acidovorans	Other bacterial taxa
Chryseobacterium	Other bacterial taxa
Myroides spp.	Other bacterial taxa
Raoultella planticola	Other bacterial taxa
Chryseobacterium gleum	Other bacterial taxa
Micrococcus	Other bacterial taxa
Rhizobium radiobacter	Other bacterial taxa
Ewingella americana	Other bacterial taxa
Vibrio	Other bacterial taxa
Buttiauxella spp.	Other bacterial taxa
Elizabethkingia	Other bacterial taxa
Chryseobacterium indologenes	Other bacterial taxa
Vibrio alginolyticus	Other bacterial taxa
Comamonas testosteroni	Other bacterial taxa
Shewanella putrefaciens	Other bacterial taxa
Elizabethkingia meningoseptica	Other bacterial taxa
Sphingomonas paucimobilis	Other bacterial taxa
Psychrobacter moraxella	Other bacterial taxa
Neisseria	Other bacterial taxa
Psychrobacter spp.	Other bacterial taxa
Photobacterium damselae	Other bacterial taxa
Edwardsiella tarda	Other bacterial taxa
Erwinia	Other bacterial taxa
Myroides flavobacterium odoratum	Other bacterial taxa
Empedobacter	Other bacterial taxa
Ralstonia	Other bacterial taxa
Shewanella	Other bacterial taxa
Sphingobacterium	Other bacterial taxa
Comamonas	Other bacterial taxa
Vibrio harveyi	Other bacterial taxa
Ralstonia insidiosa	Other bacterial taxa
Delftia comamonas acidovorans	Other bacterial taxa
Shewanella algae	Other bacterial taxa
Yersinia enterocolitica	Other bacterial taxa
Escherichia fergusonii	Other bacterial taxa
Vibrio fluvialis	Other bacterial taxa
Mannheimia	Other bacterial taxa
Rhizobium	Other bacterial taxa
Vibrio parahaemolyticus	Other bacterial taxa
Gemella cuniculi	Other bacterial taxa
Ralstonia pickettii	Other bacterial taxa
Bergeyella zoohelcum	Other bacterial taxa
Chromobacterium violaceum	Other bacterial taxa
Riemerella anatipestifer	Other bacterial taxa
Sphingobacterium multivorum	Other bacterial taxa
Yokenella regensburgei	Other bacterial taxa
Jeotgalicoccus halotolerans	Other bacterial taxa
Rhizobium spp.	Other bacterial taxa
Massilia spp.	Other bacterial taxa
Agrobacterium	Other bacterial taxa
Cedecea neteri	Other bacterial taxa
Chromobacterium	Other bacterial taxa

Delftia	Other bacterial taxa
Edwardsiella hoshinae	Other bacterial taxa
Ewingella	Other bacterial taxa
Haemophilus	Other bacterial taxa
Lactobacillus	Other bacterial taxa
Psychrobacter sanguinis	Other bacterial taxa
Sphingomonas	Other bacterial taxa
Yersinia pseudotuberculosis	Other bacterial taxa
Cedecea	Other bacterial taxa
Cedecea davisae	Other bacterial taxa
Moellerella wisconsensis	Other bacterial taxa
Rahnella enterobacter aquitilis	Other bacterial taxa
Raoultella terrigena	Other bacterial taxa
Shigella sonnei	Other bacterial taxa
Siccibacter turicensis	Other bacterial taxa
Unknown	Other bacterial taxa
Empedobacter brevis	Other bacterial taxa
Enteric gram neg rod	Other bacterial taxa
Flavobacterium	Other bacterial taxa
Aerococcus spp.	Other bacterial taxa
Alloiococcus	Other bacterial taxa
Mixta calida	Other bacterial taxa
Pandoraea	Other bacterial taxa
Bacillus spp.	Other bacterial taxa
Rouxiella chamberiensis	Other bacterial taxa
Mannheimia granulomatis	Other bacterial taxa
Pseudomonas oryzihabitans	Other bacterial taxa
Rhodococcus equi	Other bacterial taxa
Haemophilus haemoglobinophilus	Other bacterial taxa
Rhodococcus	Other bacterial taxa
Stenotrophomonas spp.	Other bacterial taxa

Table S4. Clinical specimen grouping. The 10 most common clinical specimen types (excluding the generic 'Other' code) were retained individually; all remaining specimen types were collapsed into the category 'Other clinical specimens'.

Sample	Total Isolates (n)	Analytical group	Retained as individual specimen type
Urine	283749	Urine	Yes
Skin	153571	Skin	Yes
Ear	123169	Ear	Yes
Wound	48446	Wound	Yes
Abscess	35731	Abscess	Yes
Other	28122	Other clinical specimens	No
Respiratory	14193	Respiratory	Yes
Anal-rectal	11523	Anal-rectal	Yes
Eye	10032	Eye	Yes
Urinary tract tissue	7177	Urinary tract tissue	Yes
Uterine	5917	Uterine	Yes
Vulvovaginal	5628	Other clinical specimens	No
Incision site	5342	Other clinical specimens	No
Abdominal	3709	Other clinical specimens	No
Joint	3703	Other clinical specimens	No
Tracheobronchial	3371	Other clinical specimens	No
Oropharyngeal	3363	Other clinical specimens	No
Draining tract	2862	Other clinical specimens	No
Nails	2639	Other clinical specimens	No
Feces	2419	Other clinical specimens	No
Brain	2261	Other clinical specimens	No
Bal	2252	Other clinical specimens	No
Table top	2196	Other clinical specimens	No
Cloaca	2189	Other clinical specimens	No
Biopsy	1949	Other clinical specimens	No
Mass	1484	Other clinical specimens	No
Bone	1418	Other clinical specimens	No
Source not indicated	1265	Other clinical specimens	No
Implant	1224	Other clinical specimens	No
Tissue	1057	Other clinical specimens	No
Liver	1047	Other clinical specimens	No
Genital	1017	Other clinical specimens	No
Cyst	1012	Other clinical specimens	No
Gallbladder	1000	Other clinical specimens	No
Prepuce	837	Other clinical specimens	No
Bite	749	Other clinical specimens	No
Fluid	729	Other clinical specimens	No
Foot	725	Other clinical specimens	No
Nailbed	604	Other clinical specimens	No
Hair	596	Other clinical specimens	No
Prostate	592	Other clinical specimens	No
Pending	533	Other clinical specimens	No
Blood	523	Other clinical specimens	No
Tail	441	Other clinical specimens	No
Unknown	404	Other clinical specimens	No
Bile	398	Other clinical specimens	No
Stifle	367	Other clinical specimens	No
Aspirate	354	Other clinical specimens	No
Semen	339	Other clinical specimens	No
Pyometra	337	Other clinical specimens	No

Pleural	306	Other clinical specimens	No
Gastric	303	Other clinical specimens	No
Testes	292	Other clinical specimens	No
Lymph node	281	Other clinical specimens	No
Penis	232	Other clinical specimens	No
Milk	212	Other clinical specimens	No
Fistula	209	Other clinical specimens	No
Leg	202	Other clinical specimens	No
Mastitis	189	Other clinical specimens	No
Fracture	179	Other clinical specimens	No
Bulla	152	Other clinical specimens	No
Stones	152	Other clinical specimens	No
Toenail	149	Other clinical specimens	No
Neck	138	Other clinical specimens	No
Catheter	135	Other clinical specimens	No
Nostril	127	Other clinical specimens	No
Elbow	126	Other clinical specimens	No
Swabs	113	Other clinical specimens	No
Teca	112	Other clinical specimens	No
Ulcer	103	Other clinical specimens	No
Spleen	100	Other clinical specimens	No
Colon	99	Other clinical specimens	No
Intestine	88	Other clinical specimens	No
Carpus carpal	86	Other clinical specimens	No
Hock	85	Other clinical specimens	No
Seroma	83	Other clinical specimens	No
Head	81	Other clinical specimens	No
Thoracic	75	Other clinical specimens	No
Peritoneal	65	Other clinical specimens	No
Environmental swab	59	Other clinical specimens	No
Jaw	59	Other clinical specimens	No
Tumor	53	Other clinical specimens	No
Chest	52	Other clinical specimens	No
Necropsy	52	Other clinical specimens	No
Pancreas	43	Other clinical specimens	No
Feathers	42	Other clinical specimens	No
Knee	42	Other clinical specimens	No
Sheath	42	Other clinical specimens	No
Umbilicus	42	Other clinical specimens	No
Placenta	39	Other clinical specimens	No
Granuloma	38	Other clinical specimens	No
Gingiva	37	Other clinical specimens	No
Glottis	30	Other clinical specimens	No
Nail	30	Other clinical specimens	No
Yolk sac	29	Other clinical specimens	No
Stomach	27	Other clinical specimens	No
Burn	18	Other clinical specimens	No
Blister	13	Other clinical specimens	No
Retrobulbar	13	Other clinical specimens	No
Vent	13	Other clinical specimens	No
Penile sheath	12	Other clinical specimens	No
Pericardial	12	Other clinical specimens	No
Teats	10	Other clinical specimens	No
Duodenum	7	Other clinical specimens	No
Cecum	6	Other clinical specimens	No
Pyothorax	6	Other clinical specimens	No

Embryo	4	Other clinical specimens	No
Exudate	2	Other clinical specimens	No

Table S5. Amoxicillin resistance summarised by bacterial taxon group and clinical specimen group (combinations with fewer than 5 Total Isolates (n) are omitted for stability).

**Bacteria_Group**	**Sample_Group**	**Resistant (n)**	**Total Isolates (n)**	**Resistance (%)**
Citrobacter spp.	Ear	5	5	100
Citrobacter spp.	Other clinical specimens	11	11	100
Enterobacter spp.	Urine	16	16	100
Enterobacter spp.	Abscess	7	7	100
Enterobacter spp.	Ear	7	7	100
Enterobacter spp.	Skin	25	25	100
Enterobacter spp.	Wound	12	12	100
Klebsiella spp.	Anal-rectal	83	83	100
Klebsiella spp.	Eye	40	40	100
Klebsiella spp.	Urinary tract tissue	29	29	100
Klebsiella spp.	Uterine	52	52	100
Pantoea spp.	Ear	5	5	100
Providencia spp.	Urine	84	84	100
Providencia spp.	Abscess	15	15	100
Providencia spp.	Ear	114	114	100
Providencia spp.	Respiratory	12	12	100
Providencia spp.	Skin	25	25	100
Providencia spp.	Wound	35	35	100
Serratia spp.	Other clinical specimens	7	7	100
Serratia spp.	Wound	6	6	100
Staphylococcus intermedius	Wound	6	6	100
Staphylococcus sciuri	Urine	18	18	100
Staphylococcus sciuri	Abscess	66	66	100
Staphylococcus sciuri	Ear	11	11	100
Staphylococcus sciuri	Eye	14	14	100
Staphylococcus sciuri	Other clinical specimens	73	73	100
Staphylococcus sciuri	Respiratory	18	18	100
Staphylococcus sciuri	Skin	114	114	100
Staphylococcus sciuri	Wound	79	79	100
Staphylococcus xylosus	Urine	10	10	100
Klebsiella spp.	Skin	477	478	99.79
Klebsiella spp.	Wound	368	369	99.73
Klebsiella spp.	Abscess	216	217	99.54
Klebsiella spp.	Other clinical specimens	646	650	99.38
Klebsiella spp.	Respiratory	154	155	99.35
Klebsiella spp.	Ear	258	261	98.85
Providencia spp.	Other clinical specimens	80	81	98.77
Klebsiella spp.	Urine	2212	2255	98.09

Staphylococcus xylosus	Wound	39	40	97.5
Staphylococcus xylosus	Skin	90	94	95.74
Staphylococcus epidermidis	Urine	21	22	95.45
Pantoea spp.	Skin	57	60	95
Staphylococcus intermedius	Abscess	17	18	94.44
Staphylococcus epidermidis	Abscess	84	89	94.38
Enterobacter spp.	Other clinical specimens	16	17	94.12
Staphylococcus epidermidis	Wound	90	96	93.75
Staphylococcus xylosus	Other clinical specimens	74	79	93.67
Staphylococcus xylosus	Abscess	44	47	93.62
Staphylococcus epidermidis	Urinary tract tissue	28	30	93.33
Staphylococcus xylosus	Ear	14	15	93.33
Escherichia coli	Wound	940	1009	93.16
Staphylococcus epidermidis	Ear	66	71	92.96
Staphylococcus epidermidis	Skin	105	114	92.11
Pantoea spp.	Other clinical specimens	11	12	91.67
Staphylococcus xylosus	Respiratory	33	36	91.67
Escherichia coli	Eye	64	70	91.43
Proteus mirabilis	Respiratory	41	45	91.11
Staphylococcus epidermidis	Other clinical specimens	282	313	90.1
Escherichia coli	Skin	1553	1727	89.92
Staphylococcus intermedius	Other clinical specimens	35	39	89.74
Escherichia coli	Anal-rectal	649	725	89.52
Proteus mirabilis	Wound	322	360	89.44
Staphylococcus xylosus	Eye	16	18	88.89
Escherichia coli	Respiratory	286	322	88.82
Staphylococcus pseudintermedius	Eye	518	585	88.55
Staphylococcus epidermidis	Eye	23	26	88.46
Staphylococcus intermedius	Skin	117	133	87.97
Acinetobacter spp.	Respiratory	7	8	87.5
Escherichia coli	Uterine	398	457	87.09
Enterococcus faecium	Respiratory	13	15	86.67
Staphylococcus pseudintermedius	Other clinical specimens	3477	4027	86.34
Staphylococcus pseudintermedius	Respiratory	416	484	85.95
Staphylococcus pseudintermedius	Skin	10674	12432	85.86
Escherichia coli	Abscess	756	881	85.81
Other bacterial taxa	Anal-rectal	18	21	85.71
Serratia spp.	Skin	6	7	85.71
Proteus mirabilis	Skin	1004	1185	84.73
Escherichia coli	Ear	1580	1872	84.4
Leclercia adecarboxylata	Skin	183	219	83.56
Leclercia adecarboxylata	Ear	20	24	83.33
Leclercia adecarboxylata	Wound	75	90	83.33
Staphylococcus pseudintermedius	Wound	2005	2409	83.23
Staphylococcus pseudintermedius	Urine	4665	5608	83.18
Staphylococcus pseudintermedius	Anal-rectal	197	237	83.12
Staphylococcus intermedius	Urine	49	59	83.05
Proteus mirabilis	Abscess	226	274	82.48
Staphylococcus pseudintermedius	Abscess	1482	1813	81.74
Escherichia coli	Urinary tract tissue	136	170	80
Proteus mirabilis	Anal-rectal	316	395	80
Proteus mirabilis	Uterine	12	15	80
Staphylococcus pseudintermedius	Ear	5746	7239	79.38
Staphylococcus pseudintermedius	Urinary tract tissue	461	591	78
Escherichia coli	Other clinical specimens	2497	3216	77.64
Proteus mirabilis	Urinary tract tissue	38	49	77.55

Proteus mirabilis	Ear	2078	2692	77.19
Staphylococcus xylosus	Uterine	10	13	76.92
Leclercia adecarboxylata	Respiratory	12	16	75
Staphylococcus aureus	Urine	86	117	73.5
Staphylococcus aureus	Other clinical specimens	628	862	72.85
Proteus mirabilis	Other clinical specimens	554	763	72.61
Staphylococcus spp.	Urine	63	87	72.41
Enterococcus faecium	Anal-rectal	62	86	72.09
Proteus mirabilis	Eye	18	25	72
Staphylococcus spp.	Anal-rectal	5	7	71.43
Staphylococcus spp.	Other clinical specimens	216	303	71.29
Staphylococcus intermedius	Ear	67	94	71.28
Staphylococcus aureus	Wound	297	420	70.71
Staphylococcus spp.	Skin	286	405	70.62
Staphylococcus epidermidis	Respiratory	7	10	70
Other bacterial taxa	Skin	536	771	69.52
Staphylococcus aureus	Skin	478	693	68.98
Enterococcus faecium	Wound	134	195	68.72
Staphylococcus spp.	Abscess	103	150	68.67
Other bacterial taxa	Wound	215	317	67.82
Staphylococcus aureus	Respiratory	109	162	67.28
Enterococcus faecium	Urine	751	1122	66.93
Leclercia adecarboxylata	Abscess	14	21	66.67
Leclercia adecarboxylata	Eye	4	6	66.67
Staphylococcus aureus	Abscess	166	249	66.67
Staphylococcus aureus	Urinary tract tissue	10	15	66.67
Staphylococcus intermedius	Urinary tract tissue	8	12	66.67
Staphylococcus aureus	Ear	125	188	66.49
Staphylococcus spp.	Ear	161	244	65.98
Staphylococcus spp.	Wound	90	137	65.69
Staphylococcus aureus	Eye	21	32	65.62
Enterococcus faecium	Other clinical specimens	248	378	65.61
Leclercia adecarboxylata	Other clinical specimens	41	63	65.08
Enterococcus faecium	Urinary tract tissue	13	20	65
Other bacterial taxa	Eye	64	102	62.75
Enterococcus faecium	Skin	109	174	62.64
Staphylococcus spp.	Eye	35	56	62.5
Staphylococcus spp.	Uterine	23	37	62.16
Staphylococcus intermedius	Eye	3	5	60
Staphylococcus pseudintermedius	Uterine	13	22	59.09
Enterococcus faecium	Abscess	76	129	58.91
Other bacterial taxa	Urinary tract tissue	11	19	57.89
Staphylococcus spp.	Respiratory	26	45	57.78
Staphylococcus spp.	Urinary tract tissue	18	32	56.25
Acinetobacter spp.	Ear	10	19	52.63
Acinetobacter spp.	Abscess	14	27	51.85
Enterococcus faecium	Ear	30	61	49.18
Other bacterial taxa	Other clinical specimens	475	974	48.77
Acinetobacter spp.	Other clinical specimens	18	37	48.65
Other bacterial taxa	Urine	88	188	46.81
Staphylococcus schleiferi	Eye	14	30	46.67
Other bacterial taxa	Ear	202	437	46.22
Acinetobacter spp.	Wound	5	11	45.45
Acinetobacter spp.	Skin	40	92	43.48
Acinetobacter spp.	Eye	3	7	42.86
Staphylococcus schleiferi	Other clinical specimens	138	329	41.95

Staphylococcus schleiferi	Skin	895	2143	41.76
Other bacterial taxa	Abscess	162	393	41.22
Acinetobacter spp.	Urine	4	10	40
Staphylococcus schleiferi	Anal-rectal	5	13	38.46
Staphylococcus schleiferi	Wound	43	122	35.25
Staphylococcus aureus	Uterine	2	6	33.33
Escherichia coli	Urine	7417	22387	33.13
Staphylococcus felis	Eye	13	40	32.5
Enterococcus faecium	Eye	4	13	30.77
Moraxella spp.	Skin	23	77	29.87
Other bacterial taxa	Respiratory	64	227	28.19
Staphylococcus schleiferi	Urine	25	93	26.88
Staphylococcus schleiferi	Abscess	41	158	25.95
Moraxella spp.	Other clinical specimens	13	51	25.49
Staphylococcus schleiferi	Ear	672	2839	23.67
Staphylococcus schleiferi	Uterine	4	17	23.53
Moraxella spp.	Wound	6	29	20.69
Moraxella spp.	Respiratory	3	16	18.75
Staphylococcus felis	Respiratory	9	48	18.75
Moraxella spp.	Ear	15	83	18.07
Proteus mirabilis	Urine	1190	6906	17.23
Moraxella spp.	Eye	4	26	15.38
Staphylococcus schleiferi	Respiratory	6	43	13.95
Staphylococcus felis	Abscess	11	82	13.41
Staphylococcus felis	Urine	52	431	12.06
Moraxella spp.	Abscess	2	17	11.76
Staphylococcus felis	Ear	46	392	11.73
Staphylococcus schleiferi	Urinary tract tissue	1	9	11.11
Staphylococcus felis	Other clinical specimens	10	95	10.53
Enterococcus spp.	Other clinical specimens	33	333	9.91
Other bacterial taxa	Uterine	13	138	9.42
Enterococcus spp.	Uterine	2	22	9.09
Staphylococcus felis	Skin	21	231	9.09
Enterococcus spp.	Wound	13	159	8.18
Enterococcus spp.	Abscess	10	134	7.46
Enterococcus spp.	Urine	53	731	7.25
Leclercia adecarboxylata	Urine	2	28	7.14
Staphylococcus felis	Wound	6	88	6.82
Staphylococcus felis	Urinary tract tissue	1	15	6.67
Enterococcus spp.	Ear	9	138	6.52
Streptococcus spp.	Respiratory	3	48	6.25
Streptococcus spp.	Urinary tract tissue	2	35	5.71
Streptococcus spp.	Anal-rectal	1	18	5.56
Streptococcus spp.	Urine	4	76	5.26
Enterococcus spp.	Skin	5	113	4.42
Streptococcus spp.	Eye	16	388	4.12
Streptococcus spp.	Uterine	3	79	3.8
Enterococcus spp.	Anal-rectal	6	164	3.66
Streptococcus spp.	Other clinical specimens	11	425	2.59
Enterococcus spp.	Urinary tract tissue	1	39	2.56
Streptococcus spp.	Skin	6	293	2.05
Streptococcus spp.	Wound	4	202	1.98
Streptococcus spp.	Abscess	4	228	1.75
Enterococcus faecalis	Urinary tract tissue	2	123	1.63
Enterococcus faecalis	Urine	75	4656	1.61
Enterococcus faecalis	Wound	9	674	1.34

Enterococcus faecalis	Skin	19	1582	1.2
Enterococcus faecalis	Other clinical specimens	15	1258	1.19
Enterococcus faecalis	Anal-rectal	4	411	0.97
Enterococcus faecalis	Abscess	4	423	0.95
Streptococcus spp.	Ear	1	110	0.91
Enterococcus faecalis	Ear	8	1039	0.77
Enterococcus faecalis	Eye	0	50	0
Enterococcus faecalis	Respiratory	0	32	0
Enterococcus faecalis	Uterine	0	27	0
Enterococcus spp.	Eye	0	15	0
Enterococcus spp.	Respiratory	0	8	0

Table S6. Amoxicillin/Clavulanate resistance summarised by bacterial taxon group and clinical specimen group (combinations with fewer than 5 Total Isolates (n) are omitted for stability).

**Bacteria_Group**	**Sample_Group**	**Resistant (n)**	**Total Isolates (n)**	**Resistance (%)**
Enterobacter spp.	Abscess	277	277	100
Enterobacter spp.	Anal-rectal	14	14	100
Providencia spp.	Abscess	6	6	100
Providencia spp.	Ear	57	57	100
Providencia spp.	Other clinical specimens	30	30	100
Providencia spp.	Skin	7	7	100
Providencia spp.	Wound	18	18	100
Serratia spp.	Eye	17	17	100
Serratia spp.	Urinary tract tissue	13	13	100
Serratia spp.	Uterine	5	5	100
Staphylococcus sciuri	Urine	19	19	100
Staphylococcus sciuri	Abscess	68	68	100
Staphylococcus sciuri	Ear	11	11	100
Staphylococcus sciuri	Eye	14	14	100
Staphylococcus sciuri	Other clinical specimens	72	72	100
Staphylococcus sciuri	Respiratory	19	19	100
Staphylococcus sciuri	Skin	118	118	100
Enterobacter spp.	Wound	587	589	99.66
Enterobacter spp.	Skin	626	630	99.37
Serratia spp.	Wound	250	252	99.21
Serratia spp.	Abscess	119	121	98.35
Enterobacter spp.	Eye	49	50	98
Serratia spp.	Ear	41	42	97.62
Enterobacter spp.	Other clinical specimens	679	696	97.56
Staphylococcus sciuri	Wound	77	79	97.47
Serratia spp.	Other clinical specimens	260	267	97.38
Enterobacter spp.	Respiratory	96	99	96.97
Citrobacter spp.	Wound	107	111	96.4
Enterobacter spp.	Uterine	23	24	95.83
Enterobacter spp.	Ear	156	163	95.71
Serratia spp.	Skin	156	163	95.71
Enterobacter spp.	Urinary tract tissue	20	21	95.24
Staphylococcus xylosus	Wound	39	41	95.12
Citrobacter spp.	Uterine	18	19	94.74
Enterobacter spp.	Urine	822	873	94.16
Staphylococcus xylosus	Abscess	47	50	94
Citrobacter spp.	Skin	93	99	93.94
Staphylococcus xylosus	Skin	93	99	93.94
Staphylococcus xylosus	Other clinical specimens	77	82	93.9
Serratia spp.	Urine	28	30	93.33
Staphylococcus xylosus	Ear	14	15	93.33

Serratia spp.	Respiratory	37	40	92.5
Escherichia coli	Wound	920	1002	91.82
Citrobacter spp.	Abscess	53	58	91.38
Klebsiella spp.	Wound	346	379	91.29
Staphylococcus xylosus	Urine	10	11	90.91
Providencia spp.	Urine	50	56	89.29
Staphylococcus xylosus	Respiratory	33	37	89.19
Staphylococcus xylosus	Eye	16	18	88.89
Staphylococcus epidermidis	Urine	22	25	88
Proteus mirabilis	Wound	306	350	87.43
Escherichia coli	Skin	1494	1709	87.42
Klebsiella spp.	Uterine	48	55	87.27
Escherichia coli	Eye	61	70	87.14
Escherichia coli	Anal-rectal	618	725	85.24
Escherichia coli	Uterine	390	460	84.78
Klebsiella spp.	Skin	417	494	84.41
Klebsiella spp.	Anal-rectal	68	81	83.95
Pantoea spp.	Wound	140	167	83.83
Escherichia coli	Respiratory	269	321	83.8
Proteus mirabilis	Respiratory	36	43	83.72
Leclercia adecarboxylata	Skin	183	219	83.56
Leclercia adecarboxylata	Wound	76	91	83.52
Leclercia adecarboxylata	Ear	20	24	83.33
Escherichia coli	Abscess	714	869	82.16
Proteus mirabilis	Skin	934	1140	81.93
Pantoea spp.	Abscess	74	91	81.32
Pantoea spp.	Skin	741	922	80.37
Citrobacter spp.	Ear	69	86	80.23
Escherichia coli	Ear	1497	1870	80.05
Pantoea spp.	Uterine	4	5	80
Proteus mirabilis	Uterine	12	15	80
Proteus mirabilis	Anal-rectal	315	394	79.95
Proteus mirabilis	Abscess	211	267	79.03
Klebsiella spp.	Ear	214	273	78.39
Citrobacter spp.	Other clinical specimens	158	202	78.22
Staphylococcus epidermidis	Wound	79	101	78.22
Citrobacter spp.	Anal-rectal	14	18	77.78
Enterococcus faecium	Respiratory	14	18	77.78
Other bacterial taxa	Anal-rectal	17	22	77.27
Staphylococcus xylosus	Uterine	10	13	76.92
Klebsiella spp.	Abscess	185	241	76.76
Citrobacter spp.	Respiratory	23	30	76.67
Proteus mirabilis	Ear	2016	2684	75.11
Klebsiella spp.	Eye	32	43	74.42
Pantoea spp.	Ear	72	98	73.47
Escherichia coli	Urinary tract tissue	127	173	73.41
Klebsiella spp.	Respiratory	119	163	73.01
Proteus mirabilis	Eye	18	25	72
Escherichia coli	Other clinical specimens	2286	3202	71.39
Klebsiella spp.	Other clinical specimens	489	689	70.97
Pantoea spp.	Eye	19	27	70.37
Leclercia adecarboxylata	Respiratory	11	16	68.75
Pantoea spp.	Other clinical specimens	118	172	68.6
Enterococcus faecium	Wound	155	229	67.69
Enterococcus faecium	Anal-rectal	68	102	66.67
Leclercia adecarboxylata	Abscess	14	21	66.67

Leclercia adecarboxylata	Eye	4	6	66.67
Enterococcus faecium	Urine	806	1210	66.61
Staphylococcus epidermidis	Skin	79	120	65.83
Proteus mirabilis	Urinary tract tissue	30	46	65.22
Leclercia adecarboxylata	Other clinical specimens	41	63	65.08
Enterococcus faecium	Other clinical specimens	283	435	65.06
Proteus mirabilis	Other clinical specimens	479	740	64.73
Pantoea spp.	Respiratory	25	39	64.1
Staphylococcus spp.	Anal-rectal	5	8	62.5
Enterococcus faecium	Skin	135	217	62.21
Staphylococcus epidermidis	Ear	46	74	62.16
Klebsiella spp.	Urinary tract tissue	18	29	62.07
Enterococcus faecium	Abscess	97	160	60.62
Enterococcus faecium	Urinary tract tissue	15	25	60
Staphylococcus epidermidis	Respiratory	6	10	60
Other bacterial taxa	Skin	478	841	56.84
Staphylococcus epidermidis	Abscess	51	93	54.84
Other bacterial taxa	Wound	200	366	54.64
Staphylococcus epidermidis	Other clinical specimens	171	314	54.46
Staphylococcus epidermidis	Eye	15	29	51.72
Staphylococcus intermedius	Skin	82	163	50.31
Staphylococcus pseudintermedius	Skin	6370	12761	49.92
Other bacterial taxa	Eye	56	113	49.56
Staphylococcus spp.	Wound	73	149	48.99
Staphylococcus spp.	Uterine	18	37	48.65
Staphylococcus pseudintermedius	Other clinical specimens	1974	4113	47.99
Staphylococcus spp.	Skin	210	443	47.4
Staphylococcus spp.	Urine	44	93	47.31
Staphylococcus epidermidis	Urinary tract tissue	14	30	46.67
Enterococcus faecium	Ear	31	67	46.27
Staphylococcus spp.	Eye	27	59	45.76
Staphylococcus aureus	Other clinical specimens	406	898	45.21
Staphylococcus spp.	Abscess	67	152	44.08
Staphylococcus spp.	Other clinical specimens	141	323	43.65
Staphylococcus spp.	Respiratory	20	46	43.48
Staphylococcus pseudintermedius	Wound	1071	2470	43.36
Staphylococcus pseudintermedius	Eye	257	611	42.06
Staphylococcus pseudintermedius	Respiratory	201	497	40.44
Staphylococcus pseudintermedius	Anal-rectal	95	244	38.93
Staphylococcus schleiferi	Anal-rectal	5	13	38.46
Staphylococcus intermedius	Other clinical specimens	17	45	37.78
Staphylococcus schleiferi	Skin	839	2233	37.57
Staphylococcus intermedius	Wound	3	8	37.5
Staphylococcus schleiferi	Other clinical specimens	127	343	37.03
Citrobacter spp.	Urine	57	157	36.31
Staphylococcus pseudintermedius	Ear	2654	7476	35.5
Staphylococcus pseudintermedius	Abscess	659	1869	35.26
Other bacterial taxa	Other clinical specimens	349	1029	33.92
Other bacterial taxa	Abscess	139	412	33.74
Staphylococcus aureus	Urinary tract tissue	5	15	33.33
Staphylococcus pseudintermedius	Urine	1908	5996	31.82
Klebsiella spp.	Urine	719	2378	30.24
Staphylococcus intermedius	Ear	32	108	29.63
Other bacterial taxa	Ear	141	486	29.01
Staphylococcus schleiferi	Wound	37	128	28.91
Staphylococcus schleiferi	Eye	10	35	28.57

Staphylococcus aureus	Wound	119	436	27.29
Staphylococcus spp.	Ear	72	267	26.97
Other bacterial taxa	Urine	56	209	26.79
Staphylococcus intermedius	Abscess	5	19	26.32
Staphylococcus schleiferi	Abscess	39	166	23.49
Moraxella spp.	Other clinical specimens	12	53	22.64
Moraxella spp.	Skin	19	85	22.35
Acinetobacter spp.	Respiratory	2	9	22.22
Enterococcus faecium	Eye	4	18	22.22
Other bacterial taxa	Urinary tract tissue	4	18	22.22
Staphylococcus aureus	Skin	160	728	21.98
Staphylococcus pseudintermedius	Urinary tract tissue	135	615	21.95
Staphylococcus aureus	Ear	43	196	21.94
Staphylococcus aureus	Eye	7	33	21.21
Staphylococcus schleiferi	Urine	23	111	20.72
Other bacterial taxa	Respiratory	47	230	20.43
Staphylococcus intermedius	Eye	1	5	20
Staphylococcus aureus	Abscess	51	260	19.62
Escherichia coli	Urine	4459	22756	19.59
Staphylococcus schleiferi	Ear	579	2974	19.47
Staphylococcus aureus	Respiratory	31	174	17.82
Acinetobacter spp.	Urine	2	12	16.67
Staphylococcus aureus	Uterine	1	6	16.67
Staphylococcus intermedius	Urine	11	66	16.67
Moraxella spp.	Eye	4	26	15.38
Moraxella spp.	Ear	13	85	15.29
Acinetobacter spp.	Other clinical specimens	5	33	15.15
Staphylococcus spp.	Urinary tract tissue	5	33	15.15
Enterococcus spp.	Uterine	4	28	14.29
Moraxella spp.	Wound	4	28	14.29
Staphylococcus aureus	Urine	18	127	14.17
Staphylococcus schleiferi	Respiratory	6	45	13.33
Acinetobacter spp.	Ear	3	23	13.04
Moraxella spp.	Respiratory	2	17	11.76
Staphylococcus schleiferi	Urinary tract tissue	1	9	11.11
Moraxella spp.	Abscess	2	19	10.53
Acinetobacter spp.	Abscess	3	33	9.09
Staphylococcus pseudintermedius	Uterine	2	22	9.09
Acinetobacter spp.	Skin	12	136	8.82
Acinetobacter spp.	Wound	1	12	8.33
Staphylococcus intermedius	Urinary tract tissue	1	12	8.33
Other bacterial taxa	Uterine	11	138	7.97
Enterococcus spp.	Other clinical specimens	24	385	6.23
Staphylococcus schleiferi	Uterine	1	18	5.56
Enterococcus spp.	Urine	47	891	5.27
Enterococcus spp.	Ear	8	171	4.68
Enterococcus spp.	Wound	9	221	4.07
Proteus mirabilis	Urine	272	6887	3.95
Enterococcus spp.	Abscess	6	163	3.68
Staphylococcus felis	Abscess	3	86	3.49
Leclercia adecarboxylata	Urine	1	29	3.45
Pantoea spp.	Urine	5	145	3.45
Streptococcus spp.	Urinary tract tissue	1	38	2.63
Staphylococcus felis	Urine	10	449	2.23
Enterococcus spp.	Anal-rectal	4	198	2.02
Streptococcus spp.	Respiratory	1	50	2

Enterococcus spp.	Skin	3	154	1.95
Streptococcus spp.	Urine	1	76	1.32
Enterococcus faecalis	Skin	23	1842	1.25
Enterococcus faecalis	Urine	62	5064	1.22
Enterococcus faecalis	Anal-rectal	5	448	1.12
Enterococcus faecalis	Other clinical specimens	16	1454	1.1
Staphylococcus felis	Wound	1	91	1.1
Streptococcus spp.	Other clinical specimens	5	457	1.09
Staphylococcus felis	Other clinical specimens	1	99	1.01
Enterococcus faecalis	Abscess	5	504	0.99
Enterococcus faecalis	Wound	7	827	0.85
Staphylococcus felis	Skin	2	244	0.82
Enterococcus faecalis	Ear	10	1266	0.79
Enterococcus faecalis	Urinary tract tissue	1	136	0.74
Streptococcus spp.	Eye	3	409	0.73
Streptococcus spp.	Wound	1	219	0.46
Streptococcus spp.	Abscess	1	245	0.41
Staphylococcus felis	Ear	1	408	0.25
Acinetobacter spp.	Eye	0	7	0
Enterococcus faecalis	Eye	0	62	0
Enterococcus faecalis	Respiratory	0	38	0
Enterococcus faecalis	Uterine	0	31	0
Enterococcus spp.	Eye	0	22	0
Enterococcus spp.	Respiratory	0	13	0
Enterococcus spp.	Urinary tract tissue	0	48	0
Staphylococcus felis	Eye	0	40	0
Staphylococcus felis	Respiratory	0	51	0
Staphylococcus felis	Urinary tract tissue	0	16	0
Staphylococcus intermedius	Respiratory	0	5	0
Streptococcus spp.	Anal-rectal	0	20	0
Streptococcus spp.	Ear	0	117	0
Streptococcus spp.	Skin	0	332	0
Streptococcus spp.	Uterine	0	78	0

Table S7. Cefalexin resistance summarised by bacterial taxon group and clinical specimen group (combinations with fewer than 5 Total Isolates (n) are omitted for stability).

**Bacteria_Group**	**Sample_Group**	**Resistant (n)**	**Total Isolates (n)**	**Resistance (%)**
Citrobacter spp.	Anal-rectal	19	19	100
Enterobacter spp.	Abscess	274	274	100
Enterobacter spp.	Anal-rectal	14	14	100
Enterobacter spp.	Urinary tract tissue	21	21	100
Enterobacter spp.	Uterine	23	23	100
Providencia spp.	Urine	86	86	100
Providencia spp.	Abscess	18	18	100
Providencia spp.	Ear	136	136	100
Providencia spp.	Other clinical specimens	92	92	100
Providencia spp.	Respiratory	13	13	100
Providencia spp.	Skin	27	27	100
Providencia spp.	Wound	43	43	100
Serratia spp.	Urine	30	30	100
Serratia spp.	Abscess	128	128	100
Serratia spp.	Ear	47	47	100
Serratia spp.	Eye	17	17	100
Serratia spp.	Respiratory	42	42	100
Serratia spp.	Urinary tract tissue	13	13	100
Serratia spp.	Uterine	5	5	100

Staphylococcus sciuri	Urine	16	16	100
Staphylococcus sciuri	Abscess	62	62	100
Staphylococcus sciuri	Ear	11	11	100
Staphylococcus sciuri	Eye	15	15	100
Staphylococcus sciuri	Other clinical specimens	68	68	100
Staphylococcus sciuri	Respiratory	16	16	100
Staphylococcus sciuri	Skin	106	106	100
Enterobacter spp.	Wound	583	585	99.66
Serratia spp.	Wound	260	261	99.62
Enterobacter spp.	Skin	602	606	99.34
Enterobacter spp.	Ear	148	149	99.33
Citrobacter spp.	Wound	115	116	99.14
Serratia spp.	Skin	158	160	98.75
Serratia spp.	Other clinical specimens	268	272	98.53
Enterobacter spp.	Other clinical specimens	678	689	98.4
Enterobacter spp.	Respiratory	105	107	98.13
Citrobacter spp.	Skin	99	101	98.02
Enterobacter spp.	Eye	49	50	98
Staphylococcus sciuri	Wound	71	73	97.26
Citrobacter spp.	Respiratory	34	35	97.14
Staphylococcus xylosus	Wound	42	44	95.45
Citrobacter spp.	Abscess	59	62	95.16
Citrobacter spp.	Ear	77	81	95.06
Staphylococcus xylosus	Other clinical specimens	77	81	95.06
Enterobacter spp.	Urine	821	864	95.02
Staphylococcus xylosus	Abscess	53	56	94.64
Proteus mirabilis	Wound	311	329	94.53
Citrobacter spp.	Uterine	16	17	94.12
Proteus mirabilis	Skin	891	949	93.89
Proteus mirabilis	Abscess	225	240	93.75
Staphylococcus xylosus	Skin	89	95	93.68
Citrobacter spp.	Other clinical specimens	203	217	93.55
Proteus mirabilis	Respiratory	43	46	93.48
Staphylococcus xylosus	Ear	14	15	93.33
Staphylococcus xylosus	Eye	25	27	92.59
Klebsiella spp.	Wound	350	380	92.11
Escherichia coli	Wound	899	984	91.36
Acinetobacter spp.	Respiratory	10	11	90.91
Staphylococcus xylosus	Urine	10	11	90.91
Proteus mirabilis	Eye	19	21	90.48
Proteus mirabilis	Anal-rectal	312	345	90.43
Klebsiella spp.	Uterine	50	56	89.29
Proteus mirabilis	Ear	1981	2236	88.6
Escherichia coli	Eye	63	72	87.5
Staphylococcus xylosus	Respiratory	35	40	87.5
Pantoea spp.	Wound	135	155	87.1
Escherichia coli	Skin	1411	1621	87.05
Staphylococcus epidermidis	Urine	20	23	86.96
Leclercia adecarboxylata	Skin	178	207	85.99
Leclercia adecarboxylata	Wound	72	84	85.71
Proteus mirabilis	Uterine	12	14	85.71
Escherichia coli	Uterine	390	457	85.34
Escherichia coli	Anal-rectal	604	708	85.31
Pantoea spp.	Abscess	72	85	84.71
Pantoea spp.	Skin	714	844	84.6
Klebsiella spp.	Skin	404	479	84.34

Escherichia coli	Respiratory	269	325	82.77
Klebsiella spp.	Anal-rectal	66	80	82.5
Proteus mirabilis	Urinary tract tissue	33	40	82.5
Acinetobacter spp.	Urine	9	11	81.82
Leclercia adecarboxylata	Ear	18	22	81.82
Escherichia coli	Abscess	706	863	81.81
Acinetobacter spp.	Wound	13	16	81.25
Escherichia coli	Ear	1454	1804	80.6
Pantoea spp.	Uterine	4	5	80
Acinetobacter spp.	Ear	19	24	79.17
Klebsiella spp.	Ear	209	264	79.17
Pantoea spp.	Ear	74	94	78.72
Proteus mirabilis	Other clinical specimens	516	663	77.83
Staphylococcus epidermidis	Wound	76	98	77.55
Klebsiella spp.	Abscess	193	249	77.51
Acinetobacter spp.	Abscess	30	39	76.92
Staphylococcus xylosus	Uterine	10	13	76.92
Pantoea spp.	Other clinical specimens	124	165	75.15
Klebsiella spp.	Respiratory	126	169	74.56
Pantoea spp.	Eye	20	27	74.07
Escherichia coli	Urinary tract tissue	124	168	73.81
Klebsiella spp.	Eye	31	42	73.81
Other bacterial taxa	Anal-rectal	14	19	73.68
Klebsiella spp.	Other clinical specimens	499	694	71.9
Escherichia coli	Other clinical specimens	2280	3185	71.59
Other bacterial taxa	Wound	249	359	69.36
Pantoea spp.	Respiratory	28	42	66.67
Leclercia adecarboxylata	Other clinical specimens	41	62	66.13
Citrobacter spp.	Urine	115	176	65.34
Acinetobacter spp.	Skin	88	135	65.19
Leclercia adecarboxylata	Abscess	13	20	65
Leclercia adecarboxylata	Respiratory	11	17	64.71
Staphylococcus epidermidis	Skin	75	116	64.66
Other bacterial taxa	Skin	409	637	64.21
Staphylococcus epidermidis	Respiratory	7	11	63.64
Staphylococcus spp.	Anal-rectal	5	8	62.5
Staphylococcus epidermidis	Ear	44	73	60.27
Leclercia adecarboxylata	Eye	3	5	60
Other bacterial taxa	Eye	60	103	58.25
Klebsiella spp.	Urinary tract tissue	18	31	58.06
Acinetobacter spp.	Other clinical specimens	22	38	57.89
Staphylococcus epidermidis	Abscess	51	93	54.84
Staphylococcus epidermidis	Other clinical specimens	168	309	54.37
Staphylococcus epidermidis	Eye	14	27	51.85
Staphylococcus intermedius	Skin	80	161	49.69
Staphylococcus pseudintermedius	Skin	6108	12362	49.41
Staphylococcus spp.	Wound	73	151	48.34
Staphylococcus pseudintermedius	Other clinical specimens	1920	4031	47.63
Other bacterial taxa	Urinary tract tissue	9	19	47.37
Staphylococcus spp.	Uterine	17	36	47.22
Staphylococcus spp.	Skin	198	429	46.15
Staphylococcus epidermidis	Urinary tract tissue	14	31	45.16
Staphylococcus spp.	Respiratory	22	49	44.9
Staphylococcus spp.	Eye	26	59	44.07
Other bacterial taxa	Other clinical specimens	388	888	43.69
Staphylococcus spp.	Urine	38	87	43.68

Staphylococcus spp.	Abscess	64	147	43.54
Staphylococcus pseudintermedius	Wound	1032	2392	43.14
Staphylococcus aureus	Other clinical specimens	362	850	42.59
Other bacterial taxa	Urine	67	158	42.41
Staphylococcus spp.	Other clinical specimens	130	310	41.94
Staphylococcus pseudintermedius	Eye	243	583	41.68
Staphylococcus pseudintermedius	Respiratory	196	489	40.08
Other bacterial taxa	Abscess	155	396	39.14
Staphylococcus intermedius	Wound	3	8	37.5
Staphylococcus schleiferi	Skin	801	2162	37.05
Staphylococcus schleiferi	Other clinical specimens	120	329	36.47
Staphylococcus intermedius	Other clinical specimens	16	44	36.36
Staphylococcus pseudintermedius	Anal-rectal	82	228	35.96
Other bacterial taxa	Ear	154	433	35.57
Staphylococcus pseudintermedius	Abscess	641	1830	35.03
Staphylococcus pseudintermedius	Ear	2542	7294	34.85
Staphylococcus schleiferi	Anal-rectal	4	12	33.33
Staphylococcus intermedius	Abscess	6	19	31.58
Klebsiella spp.	Urine	753	2439	30.87
Staphylococcus pseudintermedius	Urine	1700	5712	29.76
Staphylococcus intermedius	Ear	32	108	29.63
Staphylococcus schleiferi	Wound	36	125	28.8
Moraxella spp.	Skin	21	73	28.77
Acinetobacter spp.	Eye	2	7	28.57
Staphylococcus aureus	Urinary tract tissue	4	14	28.57
Staphylococcus schleiferi	Eye	10	35	28.57
Staphylococcus spp.	Ear	71	263	27
Moraxella spp.	Wound	8	30	26.67
Staphylococcus aureus	Wound	114	431	26.45
Other bacterial taxa	Respiratory	60	234	25.64
Staphylococcus schleiferi	Abscess	38	158	24.05
Moraxella spp.	Other clinical specimens	12	50	24
Staphylococcus aureus	Ear	44	197	22.34
Staphylococcus pseudintermedius	Urinary tract tissue	133	603	22.06
Staphylococcus schleiferi	Urine	23	108	21.3
Staphylococcus aureus	Skin	148	705	20.99
Moraxella spp.	Respiratory	5	25	20
Staphylococcus intermedius	Eye	1	5	20
Escherichia coli	Urine	4579	23109	19.81
Staphylococcus aureus	Abscess	52	265	19.62
Staphylococcus schleiferi	Ear	554	2908	19.05
Staphylococcus aureus	Respiratory	32	174	18.39
Moraxella spp.	Eye	4	25	16
Moraxella spp.	Abscess	3	19	15.79
Staphylococcus aureus	Eye	6	38	15.79
Staphylococcus spp.	Urinary tract tissue	5	32	15.62
Staphylococcus intermedius	Urine	10	65	15.38
Moraxella spp.	Ear	12	83	14.46
Staphylococcus aureus	Uterine	1	7	14.29
Staphylococcus schleiferi	Urinary tract tissue	1	8	12.5
Staphylococcus aureus	Urine	14	120	11.67
Staphylococcus schleiferi	Respiratory	5	44	11.36
Pantoea spp.	Urine	16	144	11.11
Staphylococcus pseudintermedius	Uterine	2	22	9.09
Staphylococcus intermedius	Urinary tract tissue	1	12	8.33
Other bacterial taxa	Uterine	11	137	8.03

Leclercia adecarboxylata	Urine	2	27	7.41
Proteus mirabilis	Urine	418	5928	7.05
Staphylococcus schleiferi	Uterine	1	17	5.88
Streptococcus spp.	Anal-rectal	1	17	5.88
Streptococcus spp.	Respiratory	2	47	4.26
Streptococcus spp.	Wound	7	193	3.63
Staphylococcus felis	Abscess	3	84	3.57
Streptococcus spp.	Urinary tract tissue	1	31	3.23
Staphylococcus felis	Urine	12	446	2.69
Streptococcus spp.	Uterine	2	78	2.56
Streptococcus spp.	Eye	6	388	1.55
Streptococcus spp.	Other clinical specimens	6	423	1.42
Streptococcus spp.	Urine	1	73	1.37
Streptococcus spp.	Abscess	3	220	1.36
Staphylococcus felis	Wound	1	91	1.1
Staphylococcus felis	Other clinical specimens	1	97	1.03
Staphylococcus felis	Skin	2	240	0.83
Streptococcus spp.	Skin	1	277	0.36
Staphylococcus felis	Ear	1	404	0.25
Staphylococcus felis	Eye	0	40	0
Staphylococcus felis	Respiratory	0	51	0
Staphylococcus felis	Urinary tract tissue	0	16	0
Staphylococcus intermedius	Respiratory	0	5	0
Streptococcus spp.	Ear	0	98	0

Table S8. Cefovecin resistance summarised by bacterial taxon group and clinical specimen group (combinations with fewer than 5 Total Isolates (n) are omitted for stability).

**Bacteria_Group**	**Sample_Group**	**Resistant (n)**	**Total Isolates (n)**	**Resistance (%)**
Acinetobacter spp.	Anal-rectal	5	5	100
Enterococcus faecalis	Urine	7	7	100
Enterococcus faecalis	Ear	5	5	100
Enterococcus faecium	Urine	6	6	100
Staphylococcus sciuri	Urine	21	21	100
Staphylococcus sciuri	Abscess	62	62	100
Staphylococcus sciuri	Ear	12	12	100
Staphylococcus sciuri	Eye	15	15	100
Staphylococcus sciuri	Other clinical specimens	65	65	100
Staphylococcus sciuri	Respiratory	14	14	100
Staphylococcus sciuri	Skin	110	110	100
Staphylococcus xylosus	Urine	10	10	100
Staphylococcus xylosus	Ear	13	13	100
Staphylococcus sciuri	Wound	67	69	97.1
Staphylococcus xylosus	Wound	33	34	97.06
Staphylococcus xylosus	Skin	85	88	96.59
Staphylococcus xylosus	Abscess	28	29	96.55
Acinetobacter spp.	Urinary tract tissue	26	27	96.3
Staphylococcus xylosus	Respiratory	23	24	95.83
Staphylococcus xylosus	Other clinical specimens	55	58	94.83
Staphylococcus xylosus	Eye	16	17	94.12
Enterococcus faecalis	Other clinical specimens	10	11	90.91
Acinetobacter spp.	Wound	273	304	89.8
Staphylococcus epidermidis	Urine	22	25	88
Acinetobacter spp.	Ear	137	159	86.16
Staphylococcus aureus	Other clinical specimens	495	583	84.91
Acinetobacter spp.	Skin	905	1087	83.26
Acinetobacter spp.	Other clinical specimens	399	484	82.44

Acinetobacter spp.	Abscess	141	178	79.21
Acinetobacter spp.	Urine	91	115	79.13
Staphylococcus epidermidis	Wound	78	99	78.79
Acinetobacter spp.	Respiratory	92	122	75.41
Staphylococcus aureus	Wound	181	251	72.11
Staphylococcus epidermidis	Skin	83	122	68.03
Acinetobacter spp.	Eye	22	33	66.67
Enterobacter spp.	Anal-rectal	8	12	66.67
Staphylococcus aureus	Eye	10	15	66.67
Staphylococcus spp.	Anal-rectal	6	9	66.67
Staphylococcus spp.	Respiratory	24	36	66.67
Staphylococcus spp.	Skin	256	387	66.15
Staphylococcus epidermidis	Ear	46	70	65.71
Staphylococcus aureus	Abscess	90	137	65.69
Staphylococcus aureus	Skin	269	412	65.29
Enterobacter spp.	Uterine	11	18	61.11
Staphylococcus spp.	Wound	71	118	60.17
Enterobacter spp.	Urinary tract tissue	6	10	60
Staphylococcus aureus	Urinary tract tissue	6	10	60
Staphylococcus epidermidis	Respiratory	6	10	60
Staphylococcus spp.	Uterine	3	5	60
Staphylococcus spp.	Abscess	62	104	59.62
Staphylococcus aureus	Respiratory	50	85	58.82
Staphylococcus spp.	Eye	29	50	58
Staphylococcus epidermidis	Abscess	52	91	57.14
Staphylococcus epidermidis	Eye	16	28	57.14
Staphylococcus spp.	Other clinical specimens	141	247	57.09
Staphylococcus spp.	Urine	51	91	56.04
Staphylococcus aureus	Ear	66	120	55
Staphylococcus epidermidis	Other clinical specimens	166	303	54.79
Staphylococcus intermedius	Skin	89	167	53.29
Enterobacter spp.	Urine	348	665	52.33
Staphylococcus spp.	Ear	108	210	51.43
Staphylococcus pseudintermedius	Skin	6301	12669	49.74
Enterobacter spp.	Wound	183	368	49.73
Staphylococcus epidermidis	Urinary tract tissue	15	31	48.39
Enterobacter spp.	Abscess	82	170	48.24
Staphylococcus pseudintermedius	Other clinical specimens	1959	4075	48.07
Enterobacter spp.	Other clinical specimens	208	456	45.61
Staphylococcus pseudintermedius	Wound	1052	2450	42.94
Staphylococcus pseudintermedius	Eye	247	599	41.24
Citrobacter spp.	Uterine	7	17	41.18
Staphylococcus pseudintermedius	Respiratory	199	489	40.7
Other bacterial taxa	Urinary tract tissue	8	20	40
Escherichia coli	Skin	679	1731	39.23
Staphylococcus intermedius	Other clinical specimens	18	46	39.13
Other bacterial taxa	Ear	158	409	38.63
Staphylococcus schleiferi	Skin	843	2205	38.23
Staphylococcus schleiferi	Other clinical specimens	129	339	38.05
Staphylococcus pseudintermedius	Anal-rectal	88	234	37.61
Staphylococcus intermedius	Wound	3	8	37.5
Escherichia coli	Wound	378	1015	37.24
Staphylococcus intermedius	Abscess	7	19	36.84
Citrobacter spp.	Ear	28	78	35.9
Acinetobacter spp.	Uterine	21	59	35.59
Staphylococcus pseudintermedius	Ear	2596	7395	35.1

Staphylococcus pseudintermedius	Abscess	652	1860	35.05
Enterobacter spp.	Skin	148	429	34.5
Staphylococcus schleiferi	Anal-rectal	4	12	33.33
Enterobacter spp.	Ear	33	101	32.67
Staphylococcus pseudintermedius	Urine	1908	5968	31.97
Enterobacter spp.	Respiratory	21	66	31.82
Staphylococcus intermedius	Ear	34	109	31.19
Escherichia coli	Respiratory	98	324	30.25
Other bacterial taxa	Abscess	90	309	29.13
Klebsiella spp.	Uterine	16	56	28.57
Escherichia coli	Anal-rectal	201	724	27.76
Citrobacter spp.	Respiratory	7	26	26.92
Staphylococcus schleiferi	Wound	33	123	26.83
Escherichia coli	Other clinical specimens	863	3233	26.69
Escherichia coli	Abscess	230	879	26.17
Other bacterial taxa	Eye	27	104	25.96
Escherichia coli	Eye	18	71	25.35
Other bacterial taxa	Wound	114	452	25.22
Enterobacter spp.	Eye	8	32	25
Other bacterial taxa	Other clinical specimens	223	899	24.81
Staphylococcus aureus	Urine	27	110	24.55
Staphylococcus schleiferi	Eye	8	33	24.24
Other bacterial taxa	Skin	166	704	23.58
Escherichia coli	Ear	436	1885	23.13
Other bacterial taxa	Respiratory	34	147	23.13
Staphylococcus schleiferi	Abscess	37	163	22.7
Citrobacter spp.	Urine	33	146	22.6
Staphylococcus schleiferi	Urine	25	111	22.52
Other bacterial taxa	Anal-rectal	4	18	22.22
Staphylococcus pseudintermedius	Urinary tract tissue	133	610	21.8
Citrobacter spp.	Anal-rectal	3	14	21.43
Klebsiella spp.	Anal-rectal	17	82	20.73
Klebsiella spp.	Urinary tract tissue	6	29	20.69
Escherichia coli	Urinary tract tissue	35	174	20.11
Serratia spp.	Uterine	1	5	20
Staphylococcus intermedius	Eye	1	5	20
Staphylococcus spp.	Urinary tract tissue	5	25	20
Klebsiella spp.	Respiratory	33	167	19.76
Staphylococcus schleiferi	Ear	577	2930	19.69
Klebsiella spp.	Other clinical specimens	136	696	19.54
Other bacterial taxa	Urine	37	191	19.37
Escherichia coli	Urine	4475	23304	19.2
Proteus mirabilis	Respiratory	8	45	17.78
Citrobacter spp.	Other clinical specimens	35	198	17.68
Moraxella spp.	Respiratory	3	17	17.65
Klebsiella spp.	Urine	422	2426	17.39
Klebsiella spp.	Wound	66	383	17.23
Staphylococcus intermedius	Urine	11	65	16.92
Klebsiella spp.	Skin	84	502	16.73
Moraxella spp.	Skin	13	79	16.46
Other bacterial taxa	Uterine	4	25	16
Citrobacter spp.	Abscess	8	55	14.55
Klebsiella spp.	Abscess	35	243	14.4
Leclercia adecarboxylata	Abscess	3	21	14.29
Citrobacter spp.	Wound	14	99	14.14
Escherichia coli	Uterine	64	456	14.04

Klebsiella spp.	Ear	38	272	13.97
Moraxella spp.	Wound	4	30	13.33
Serratia spp.	Abscess	13	115	11.3
Staphylococcus schleiferi	Urinary tract tissue	1	9	11.11
Pantoea spp.	Respiratory	4	37	10.81
Providencia spp.	Urine	9	85	10.59
Moraxella spp.	Ear	9	87	10.34
Streptococcus spp.	Urine	7	69	10.14
Citrobacter spp.	Skin	8	79	10.13
Staphylococcus pseudintermedius	Uterine	2	20	10
Staphylococcus schleiferi	Respiratory	4	42	9.52
Streptococcus spp.	Respiratory	3	32	9.38
Staphylococcus intermedius	Urinary tract tissue	1	12	8.33
Pantoea spp.	Other clinical specimens	13	164	7.93
Serratia spp.	Ear	3	38	7.89
Serratia spp.	Respiratory	3	39	7.69
Proteus mirabilis	Uterine	1	15	6.67
Providencia spp.	Other clinical specimens	6	90	6.67
Proteus mirabilis	Wound	24	363	6.61
Proteus mirabilis	Other clinical specimens	50	767	6.52
Serratia spp.	Skin	10	162	6.17
Proteus mirabilis	Urinary tract tissue	3	49	6.12
Proteus mirabilis	Skin	72	1190	6.05
Streptococcus spp.	Wound	12	199	6.03
Pantoea spp.	Abscess	5	85	5.88
Streptococcus spp.	Other clinical specimens	22	382	5.76
Streptococcus spp.	Urinary tract tissue	2	36	5.56
Moraxella spp.	Other clinical specimens	3	56	5.36
Moraxella spp.	Abscess	1	19	5.26
Proteus mirabilis	Abscess	14	284	4.93
Pantoea spp.	Skin	42	855	4.91
Serratia spp.	Other clinical specimens	12	261	4.6
Providencia spp.	Wound	2	44	4.55
Pantoea spp.	Wound	7	158	4.43
Pantoea spp.	Ear	4	91	4.4
Leclercia adecarboxylata	Ear	1	24	4.17
Moraxella spp.	Eye	1	25	4
Serratia spp.	Wound	9	251	3.59
Leclercia adecarboxylata	Urine	1	28	3.57
Pantoea spp.	Urine	5	142	3.52
Providencia spp.	Skin	1	29	3.45
Providencia spp.	Ear	5	146	3.42
Serratia spp.	Urine	1	30	3.33
Streptococcus spp.	Skin	10	301	3.32
Leclercia adecarboxylata	Other clinical specimens	2	63	3.17
Proteus mirabilis	Ear	84	2772	3.03
Proteus mirabilis	Urine	205	6908	2.97
Staphylococcus felis	Urine	11	449	2.45
Staphylococcus felis	Abscess	2	85	2.35
Klebsiella spp.	Eye	1	43	2.33
Leclercia adecarboxylata	Wound	2	89	2.25
Streptococcus spp.	Eye	8	385	2.08
Streptococcus spp.	Ear	2	108	1.85
Leclercia adecarboxylata	Skin	3	216	1.39
Proteus mirabilis	Anal-rectal	5	401	1.25
Staphylococcus felis	Wound	1	91	1.1

Streptococcus spp.	Abscess	2	182	1.1
Staphylococcus felis	Other clinical specimens	1	99	1.01
Staphylococcus felis	Skin	2	241	0.83
Staphylococcus felis	Ear	2	407	0.49
Leclercia adecarboxylata	Eye	0	6	0
Leclercia adecarboxylata	Respiratory	0	16	0
Pantoea spp.	Eye	0	26	0
Pantoea spp.	Uterine	0	5	0
Proteus mirabilis	Eye	0	25	0
Providencia spp.	Abscess	0	15	0
Providencia spp.	Respiratory	0	12	0
Serratia spp.	Eye	0	18	0
Serratia spp.	Urinary tract tissue	0	12	0
Staphylococcus felis	Eye	0	40	0
Staphylococcus felis	Respiratory	0	51	0
Staphylococcus felis	Urinary tract tissue	0	16	0
Staphylococcus intermedius	Respiratory	0	5	0
Streptococcus spp.	Anal-rectal	0	17	0
Streptococcus spp.	Uterine	0	11	0

Table S9. Cefpodoxime resistance summarised by bacterial taxon group and clinical specimen group (combinations with fewer than 5 Total Isolates (n) are omitted for stability).

**Bacteria_Group**	**Sample_Group**	**Resistant (n)**	**Total Isolates (n)**	**Resistance (%)**
Staphylococcus sciuri	Urine	19	19	100
Staphylococcus sciuri	Abscess	66	66	100
Staphylococcus sciuri	Ear	11	11	100
Staphylococcus sciuri	Eye	15	15	100
Staphylococcus sciuri	Other clinical specimens	66	66	100
Staphylococcus sciuri	Respiratory	15	15	100
Staphylococcus sciuri	Skin	113	113	100
Staphylococcus sciuri	Wound	73	75	97.33
Staphylococcus xylosus	Wound	38	40	95
Staphylococcus xylosus	Other clinical specimens	75	79	94.94
Staphylococcus xylosus	Eye	16	17	94.12
Staphylococcus xylosus	Skin	88	94	93.62
Staphylococcus xylosus	Abscess	40	43	93.02
Acinetobacter spp.	Urinary tract tissue	24	26	92.31
Staphylococcus xylosus	Ear	12	13	92.31
Enterobacter spp.	Uterine	21	23	91.3
Staphylococcus xylosus	Urine	10	11	90.91
Staphylococcus xylosus	Respiratory	28	32	87.5
Staphylococcus epidermidis	Urine	20	23	86.96
Enterobacter spp.	Urine	699	846	82.62
Enterobacter spp.	Eye	37	45	82.22
Enterobacter spp.	Abscess	209	255	81.96
Staphylococcus xylosus	Uterine	9	11	81.82
Enterobacter spp.	Ear	120	147	81.63
Enterobacter spp.	Respiratory	79	98	80.61
Enterobacter spp.	Other clinical specimens	518	643	80.56
Enterobacter spp.	Urinary tract tissue	16	20	80
Enterobacter spp.	Wound	437	547	79.89
Staphylococcus epidermidis	Wound	77	99	77.78
Enterobacter spp.	Anal-rectal	10	13	76.92
Enterobacter spp.	Skin	418	575	72.7
Acinetobacter spp.	Wound	203	286	70.98
Staphylococcus epidermidis	Skin	80	120	66.67

Acinetobacter spp.	Other clinical specimens	297	449	66.15
Acinetobacter spp.	Abscess	103	164	62.8
Staphylococcus spp.	Anal-rectal	5	8	62.5
Staphylococcus epidermidis	Ear	46	74	62.16
Acinetobacter spp.	Ear	89	148	60.14
Staphylococcus epidermidis	Respiratory	6	10	60
Acinetobacter spp.	Urine	64	109	58.72
Acinetobacter spp.	Respiratory	64	109	58.72
Staphylococcus spp.	Urine	45	79	56.96
Staphylococcus epidermidis	Other clinical specimens	172	313	54.95
Acinetobacter spp.	Skin	548	1000	54.8
Staphylococcus epidermidis	Abscess	50	92	54.35
Staphylococcus epidermidis	Eye	15	28	53.57
Acinetobacter spp.	Eye	18	34	52.94
Staphylococcus intermedius	Skin	84	164	51.22
Staphylococcus pseudintermedius	Skin	6337	12714	49.84
Staphylococcus spp.	Skin	214	435	49.2
Staphylococcus spp.	Wound	70	143	48.95
Staphylococcus spp.	Respiratory	21	43	48.84
Staphylococcus spp.	Uterine	16	33	48.48
Staphylococcus pseudintermedius	Other clinical specimens	1953	4083	47.83
Staphylococcus spp.	Abscess	71	149	47.65
Staphylococcus aureus	Other clinical specimens	404	848	47.64
Staphylococcus epidermidis	Urinary tract tissue	14	30	46.67
Staphylococcus spp.	Eye	26	58	44.83
Staphylococcus spp.	Other clinical specimens	136	310	43.87
Staphylococcus pseudintermedius	Wound	1058	2448	43.22
Staphylococcus pseudintermedius	Eye	255	607	42.01
Staphylococcus pseudintermedius	Respiratory	199	489	40.7
Escherichia coli	Skin	688	1740	39.54
Klebsiella spp.	Uterine	22	56	39.29
Staphylococcus intermedius	Other clinical specimens	18	46	39.13
Staphylococcus schleiferi	Skin	841	2227	37.76
Staphylococcus intermedius	Wound	3	8	37.5
Escherichia coli	Wound	378	1018	37.13
Staphylococcus schleiferi	Other clinical specimens	125	339	36.87
Staphylococcus pseudintermedius	Anal-rectal	86	235	36.6
Staphylococcus pseudintermedius	Ear	2619	7402	35.38
Staphylococcus pseudintermedius	Abscess	655	1862	35.18
Staphylococcus schleiferi	Anal-rectal	4	12	33.33
Other bacterial taxa	Respiratory	108	326	33.13
Citrobacter spp.	Wound	37	116	31.9
Staphylococcus pseudintermedius	Urine	1879	5936	31.65
Citrobacter spp.	Uterine	6	19	31.58
Other bacterial taxa	Urinary tract tissue	6	19	31.58
Staphylococcus intermedius	Abscess	6	19	31.58
Escherichia coli	Respiratory	97	326	29.75
Staphylococcus intermedius	Ear	32	108	29.63
Staphylococcus spp.	Ear	72	245	29.39
Citrobacter spp.	Urine	52	179	29.05
Staphylococcus aureus	Wound	120	415	28.92
Other bacterial taxa	Anal-rectal	4	14	28.57
Staphylococcus aureus	Urinary tract tissue	4	14	28.57
Staphylococcus schleiferi	Wound	36	126	28.57
Other bacterial taxa	Ear	133	469	28.36
Citrobacter spp.	Other clinical specimens	63	223	28.25

Other bacterial taxa	Other clinical specimens	326	1206	27.03
Escherichia coli	Anal-rectal	196	730	26.85
Escherichia coli	Eye	19	71	26.76
Escherichia coli	Other clinical specimens	874	3276	26.68
Staphylococcus schleiferi	Eye	9	34	26.47
Escherichia coli	Abscess	232	889	26.1
Klebsiella spp.	Urinary tract tissue	8	31	25.81
Acinetobacter spp.	Uterine	13	51	25.49
Staphylococcus aureus	Skin	165	691	23.88
Citrobacter spp.	Respiratory	8	34	23.53
Staphylococcus aureus	Eye	7	30	23.33
Staphylococcus aureus	Ear	43	186	23.12
Staphylococcus schleiferi	Abscess	38	165	23.03
Other bacterial taxa	Wound	105	458	22.93
Other bacterial taxa	Eye	24	105	22.86
Citrobacter spp.	Abscess	14	62	22.58
Staphylococcus schleiferi	Urine	25	112	22.32
Citrobacter spp.	Anal-rectal	4	18	22.22
Escherichia coli	Ear	424	1915	22.14
Staphylococcus pseudintermedius	Urinary tract tissue	134	613	21.86
Staphylococcus aureus	Abscess	53	246	21.54
Other bacterial taxa	Skin	153	711	21.52
Klebsiella spp.	Other clinical specimens	150	710	21.13
Klebsiella spp.	Respiratory	35	168	20.83
Klebsiella spp.	Anal-rectal	17	82	20.73
Staphylococcus intermedius	Eye	1	5	20
Klebsiella spp.	Urine	489	2464	19.85
Staphylococcus schleiferi	Ear	580	2960	19.59
Klebsiella spp.	Skin	98	507	19.33
Staphylococcus aureus	Urine	17	89	19.1
Staphylococcus aureus	Respiratory	31	163	19.02
Escherichia coli	Urinary tract tissue	33	176	18.75
Klebsiella spp.	Wound	72	384	18.75
Escherichia coli	Urine	4344	23505	18.48
Citrobacter spp.	Skin	19	104	18.27
Other bacterial taxa	Abscess	79	434	18.2
Staphylococcus intermedius	Urine	12	66	18.18
Providencia spp.	Urine	15	86	17.44
Citrobacter spp.	Ear	16	92	17.39
Proteus mirabilis	Respiratory	8	46	17.39
Klebsiella spp.	Abscess	42	245	17.14
Staphylococcus aureus	Uterine	1	6	16.67
Other bacterial taxa	Urine	29	181	16.02
Escherichia coli	Uterine	64	460	13.91
Staphylococcus spp.	Urinary tract tissue	4	29	13.79
Staphylococcus pseudintermedius	Uterine	3	23	13.04
Serratia spp.	Ear	1	8	12.5
Klebsiella spp.	Ear	33	281	11.74
Staphylococcus schleiferi	Respiratory	5	44	11.36
Staphylococcus schleiferi	Urinary tract tissue	1	9	11.11
Providencia spp.	Other clinical specimens	10	92	10.87
Serratia spp.	Other clinical specimens	4	40	10
Leclercia adecarboxylata	Abscess	2	21	9.52
Streptococcus spp.	Urine	6	70	8.57
Staphylococcus intermedius	Urinary tract tissue	1	12	8.33
Pantoea spp.	Respiratory	3	39	7.69

Providencia spp.	Skin	2	29	6.9
Serratia spp.	Wound	2	29	6.9
Proteus mirabilis	Uterine	1	15	6.67
Moraxella spp.	Skin	5	76	6.58
Proteus mirabilis	Other clinical specimens	49	777	6.31
Proteus mirabilis	Urinary tract tissue	3	48	6.25
Providencia spp.	Abscess	1	16	6.25
Proteus mirabilis	Skin	73	1196	6.1
Staphylococcus schleiferi	Uterine	1	17	5.88
Streptococcus spp.	Urinary tract tissue	2	34	5.88
Proteus mirabilis	Wound	21	366	5.74
Streptococcus spp.	Anal-rectal	1	18	5.56
Streptococcus spp.	Wound	10	189	5.29
Serratia spp.	Abscess	1	19	5.26
Streptococcus spp.	Other clinical specimens	20	397	5.04
Other bacterial taxa	Uterine	7	141	4.96
Klebsiella spp.	Eye	2	43	4.65
Proteus mirabilis	Abscess	13	282	4.61
Providencia spp.	Wound	2	45	4.44
Streptococcus spp.	Uterine	3	77	3.9
Serratia spp.	Skin	1	27	3.7
Streptococcus spp.	Skin	11	303	3.63
Leclercia adecarboxylata	Urine	1	28	3.57
Moraxella spp.	Wound	1	28	3.57
Staphylococcus felis	Abscess	3	86	3.49
Providencia spp.	Ear	5	147	3.4
Proteus mirabilis	Urine	206	6941	2.97
Pantoea spp.	Other clinical specimens	5	174	2.87
Pantoea spp.	Urine	4	145	2.76
Streptococcus spp.	Respiratory	1	38	2.63
Proteus mirabilis	Ear	72	2784	2.59
Pantoea spp.	Skin	23	921	2.5
Staphylococcus felis	Eye	1	40	2.5
Staphylococcus felis	Urine	11	449	2.45
Moraxella spp.	Ear	2	85	2.35
Streptococcus spp.	Eye	8	365	2.19
Pantoea spp.	Abscess	2	93	2.15
Pantoea spp.	Ear	2	97	2.06
Streptococcus spp.	Ear	2	105	1.9
Moraxella spp.	Other clinical specimens	1	53	1.89
Streptococcus spp.	Abscess	3	201	1.49
Staphylococcus felis	Wound	1	91	1.1
Staphylococcus felis	Other clinical specimens	1	99	1.01
Proteus mirabilis	Anal-rectal	4	403	0.99
Staphylococcus felis	Skin	2	241	0.83
Pantoea spp.	Wound	1	168	0.6
Leclercia adecarboxylata	Skin	1	217	0.46
Staphylococcus felis	Ear	1	408	0.25
Leclercia adecarboxylata	Ear	0	23	0
Leclercia adecarboxylata	Eye	0	6	0
Leclercia adecarboxylata	Other clinical specimens	0	63	0
Leclercia adecarboxylata	Respiratory	0	16	0
Leclercia adecarboxylata	Wound	0	89	0
Moraxella spp.	Abscess	0	18	0
Moraxella spp.	Eye	0	25	0
Moraxella spp.	Respiratory	0	16	0

Pantoea spp.	Eye	0	27	0
Pantoea spp.	Uterine	0	5	0
Proteus mirabilis	Eye	0	25	0
Providencia spp.	Respiratory	0	12	0
Serratia spp.	Urine	0	8	0
Staphylococcus felis	Respiratory	0	50	0
Staphylococcus felis	Urinary tract tissue	0	16	0
Staphylococcus intermedius	Respiratory	0	5	0

Table S10. Ceftazidime resistance summarised by bacterial taxon group and clinical specimen group (combinations with fewer than 5 Total Isolates (n) are omitted for stability).

Bacteria_Group	Sample_Group	Resistant (n)	Total Isolates (n)	Resistance (%)
Staphylococcus pseudintermedius	Ear	3	5	60
Serratia spp.	Urinary tract tissue	7	14	50
Serratia spp.	Ear	18	46	39.13
Enterobacter spp.	Uterine	8	22	36.36
Other bacterial taxa	Urinary tract tissue	5	14	35.71
Klebsiella spp.	Uterine	19	55	34.55
Escherichia coli	Skin	531	1630	32.58
Serratia spp.	Respiratory	13	40	32.5
Serratia spp.	Urine	9	28	32.14
Escherichia coli	Wound	301	959	31.39
Enterobacter spp.	Anal-rectal	4	13	30.77
Serratia spp.	Skin	44	153	28.76
Citrobacter spp.	Uterine	5	18	27.78
Enterobacter spp.	Urine	234	845	27.69
Escherichia coli	Respiratory	83	316	26.27
Serratia spp.	Abscess	32	126	25.4
Klebsiella spp.	Urinary tract tissue	7	28	25
Serratia spp.	Wound	62	259	23.94
Serratia spp.	Other clinical specimens	64	273	23.44
Escherichia coli	Anal-rectal	161	689	23.37
Klebsiella spp.	Anal-rectal	18	80	22.5
Serratia spp.	Eye	4	18	22.22
Escherichia coli	Other clinical specimens	688	3125	22.02
Escherichia coli	Abscess	177	846	20.92
Klebsiella spp.	Other clinical specimens	145	698	20.77
Escherichia coli	Eye	14	69	20.29
Klebsiella spp.	Urine	473	2416	19.58
Klebsiella spp.	Skin	92	497	18.51
Enterobacter spp.	Other clinical specimens	127	689	18.43
Klebsiella spp.	Wound	70	385	18.18
Escherichia coli	Ear	323	1825	17.7
Proteus mirabilis	Respiratory	8	46	17.39
Klebsiella spp.	Respiratory	29	167	17.37
Klebsiella spp.	Abscess	40	246	16.26
Citrobacter spp.	Urine	28	175	16
Enterobacter spp.	Abscess	43	276	15.58
Enterobacter spp.	Wound	90	578	15.57
Escherichia coli	Urine	3485	22828	15.27
Providencia spp.	Urine	12	83	14.46
Enterobacter spp.	Urinary tract tissue	3	21	14.29
Other bacterial taxa	Eye	13	94	13.83
Escherichia coli	Urinary tract tissue	24	174	13.79
Citrobacter spp.	Other clinical specimens	30	220	13.64
Enterobacter spp.	Respiratory	14	103	13.59

Other bacterial taxa	Urine	20	150	13.33
Other bacterial taxa	Abscess	39	306	12.75
Other bacterial taxa	Other clinical specimens	117	982	11.91
Enterobacter spp.	Ear	19	160	11.88
Citrobacter spp.	Anal-rectal	2	17	11.76
Citrobacter spp.	Respiratory	4	34	11.76
Other bacterial taxa	Uterine	3	26	11.54
Enterobacter spp.	Skin	71	625	11.36
Klebsiella spp.	Ear	31	277	11.19
Citrobacter spp.	Abscess	7	63	11.11
Other bacterial taxa	Anal-rectal	2	18	11.11
Providencia spp.	Other clinical specimens	10	91	10.99
Escherichia coli	Uterine	48	444	10.81
Leclercia adecarboxylata	Abscess	2	19	10.53
Citrobacter spp.	Skin	11	105	10.48
Providencia spp.	Skin	3	29	10.34
Other bacterial taxa	Wound	42	452	9.29
Other bacterial taxa	Skin	56	614	9.12
Acinetobacter spp.	Urine	9	112	8.04
Citrobacter spp.	Wound	9	119	7.56
Other bacterial taxa	Respiratory	16	212	7.55
Acinetobacter spp.	Urinary tract tissue	2	28	7.14
Acinetobacter spp.	Eye	2	32	6.25
Other bacterial taxa	Ear	15	260	5.77
Pseudomonas spp.	Urinary tract tissue	6	104	5.77
Proteus mirabilis	Skin	63	1159	5.44
Citrobacter spp.	Ear	5	93	5.38
Klebsiella spp.	Eye	2	40	5
Moraxella spp.	Skin	3	65	4.62
Proteus mirabilis	Other clinical specimens	35	765	4.58
Providencia spp.	Wound	2	44	4.55
Leclercia adecarboxylata	Ear	1	23	4.35
Proteus mirabilis	Urinary tract tissue	2	48	4.17
Enterobacter spp.	Eye	2	50	4
Acinetobacter spp.	Other clinical specimens	17	464	3.66
Acinetobacter spp.	Uterine	2	55	3.64
Pseudomonas spp.	Anal-rectal	3	84	3.57
Moraxella spp.	Wound	1	29	3.45
Pseudomonas spp.	Respiratory	27	812	3.33
Proteus mirabilis	Wound	12	364	3.3
Proteus mirabilis	Abscess	9	278	3.24
Acinetobacter spp.	Wound	9	284	3.17
Pantoea spp.	Ear	3	96	3.12
Pantoea spp.	Wound	5	164	3.05
Pseudomonas spp.	Wound	35	1146	3.05
Pseudomonas spp.	Ear	222	7414	2.99
Pantoea spp.	Other clinical specimens	5	172	2.91
Pseudomonas spp.	Eye	8	289	2.77
Pantoea spp.	Urine	4	148	2.7
Proteus mirabilis	Ear	73	2710	2.69
Pseudomonas spp.	Urine	29	1092	2.66
Proteus mirabilis	Urine	179	6819	2.63
Acinetobacter spp.	Abscess	4	155	2.58
Acinetobacter spp.	Respiratory	3	117	2.56
Pseudomonas spp.	Other clinical specimens	55	2302	2.39
Pantoea spp.	Respiratory	1	42	2.38

Pseudomonas spp.	Abscess	14	638	2.19
Pantoea spp.	Abscess	2	92	2.17
Pseudomonas spp.	Skin	64	3029	2.11
Pantoea spp.	Skin	14	911	1.54
Providencia spp.	Ear	2	143	1.4
Acinetobacter spp.	Ear	2	150	1.33
Moraxella spp.	Ear	1	78	1.28
Proteus mirabilis	Anal-rectal	5	393	1.27
Acinetobacter spp.	Skin	12	1009	1.19
Leclercia adecarboxylata	Wound	1	91	1.1
Leclercia adecarboxylata	Skin	1	219	0.46
Acinetobacter spp.	Anal-rectal	0	5	0
Leclercia adecarboxylata	Urine	0	25	0
Leclercia adecarboxylata	Eye	0	6	0
Leclercia adecarboxylata	Other clinical specimens	0	61	0
Leclercia adecarboxylata	Respiratory	0	17	0
Moraxella spp.	Abscess	0	20	0
Moraxella spp.	Eye	0	25	0
Moraxella spp.	Other clinical specimens	0	52	0
Moraxella spp.	Respiratory	0	24	0
Pantoea spp.	Eye	0	27	0
Pantoea spp.	Uterine	0	5	0
Proteus mirabilis	Eye	0	25	0
Proteus mirabilis	Uterine	0	14	0
Providencia spp.	Abscess	0	17	0
Providencia spp.	Respiratory	0	13	0
Pseudomonas spp.	Uterine	0	58	0
Serratia spp.	Uterine	0	5	0

Table S11. Imipenem resistance summarised by bacterial taxon group and clinical specimen group (combinations with fewer than 5 Total Isolates (n) are omitted for stability).

**Bacteria_Group**	**Sample_Group**	**Resistant (n)**	**Total Isolates (n)**	**Resistance (%)**
Staphylococcus sciuri	Urine	16	16	100
Staphylococcus sciuri	Abscess	57	57	100
Staphylococcus sciuri	Ear	11	11	100
Staphylococcus sciuri	Eye	12	12	100
Staphylococcus sciuri	Other clinical specimens	57	57	100
Staphylococcus sciuri	Respiratory	17	17	100
Staphylococcus sciuri	Skin	80	80	100
Staphylococcus xylosus	Urine	10	10	100
Staphylococcus sciuri	Wound	51	52	98.08
Staphylococcus xylosus	Abscess	36	37	97.3
Staphylococcus xylosus	Wound	33	35	94.29
Staphylococcus xylosus	Skin	73	78	93.59
Staphylococcus xylosus	Ear	13	14	92.86
Staphylococcus xylosus	Other clinical specimens	64	69	92.75
Staphylococcus xylosus	Eye	11	12	91.67
Staphylococcus epidermidis	Urine	20	22	90.91
Staphylococcus xylosus	Respiratory	30	33	90.91
Staphylococcus epidermidis	Wound	57	74	77.03
Staphylococcus xylosus	Uterine	6	8	75
Staphylococcus spp.	Anal-rectal	4	6	66.67
Staphylococcus epidermidis	Skin	61	93	65.59
Staphylococcus epidermidis	Ear	43	69	62.32
Staphylococcus epidermidis	Abscess	42	75	56
Staphylococcus epidermidis	Other clinical specimens	149	268	55.6

Staphylococcus epidermidis	Respiratory	5	9	55.56
Staphylococcus intermedius	Skin	77	143	53.85
Staphylococcus epidermidis	Eye	10	19	52.63
Staphylococcus pseudintermedius	Skin	5925	11763	50.37
Staphylococcus epidermidis	Urinary tract tissue	15	30	50
Staphylococcus intermedius	Wound	3	6	50
Staphylococcus spp.	Eye	23	46	50
Staphylococcus spp.	Skin	164	333	49.25
Staphylococcus pseudintermedius	Other clinical specimens	1855	3833	48.4
Staphylococcus spp.	Uterine	15	31	48.39
Staphylococcus spp.	Respiratory	21	45	46.67
Staphylococcus spp.	Wound	51	111	45.95
Staphylococcus spp.	Urine	38	83	45.78
Staphylococcus pseudintermedius	Eye	239	524	45.61
Staphylococcus pseudintermedius	Wound	978	2233	43.8
Staphylococcus aureus	Other clinical specimens	340	780	43.59
Staphylococcus spp.	Abscess	54	124	43.55
Staphylococcus spp.	Other clinical specimens	109	252	43.25
Proteus mirabilis	Urine	3	7	42.86
Staphylococcus schleiferi	Eye	8	19	42.11
Staphylococcus pseudintermedius	Respiratory	197	471	41.83
Staphylococcus intermedius	Other clinical specimens	16	40	40
Staphylococcus schleiferi	Skin	733	1834	39.97
Staphylococcus pseudintermedius	Anal-rectal	72	183	39.34
Staphylococcus schleiferi	Other clinical specimens	112	286	39.16
Staphylococcus intermedius	Abscess	7	18	38.89
Staphylococcus pseudintermedius	Ear	2482	6817	36.41
Staphylococcus pseudintermedius	Abscess	616	1717	35.88
Staphylococcus pseudintermedius	Urine	1697	5295	32.05
Staphylococcus spp.	Ear	64	203	31.53
Staphylococcus intermedius	Ear	31	100	31
Staphylococcus aureus	Urinary tract tissue	4	13	30.77
Staphylococcus aureus	Wound	109	358	30.45
Staphylococcus schleiferi	Wound	31	103	30.1
Staphylococcus schleiferi	Anal-rectal	3	10	30
Staphylococcus schleiferi	Abscess	37	137	27.01
Serratia spp.	Wound	5	20	25
Staphylococcus schleiferi	Urine	21	88	23.86
Staphylococcus aureus	Skin	139	591	23.52
Staphylococcus aureus	Ear	36	162	22.22
Staphylococcus pseudintermedius	Urinary tract tissue	118	545	21.65
Staphylococcus schleiferi	Ear	503	2488	20.22
Other bacterial taxa	Anal-rectal	2	10	20
Staphylococcus intermedius	Eye	1	5	20
Staphylococcus aureus	Eye	5	26	19.23
Staphylococcus aureus	Abscess	42	219	19.18
Staphylococcus aureus	Respiratory	28	151	18.54
Staphylococcus intermedius	Urine	9	53	16.98
Staphylococcus aureus	Uterine	1	6	16.67
Staphylococcus aureus	Urine	16	116	13.79
Staphylococcus schleiferi	Respiratory	5	38	13.16
Other bacterial taxa	Wound	40	337	11.87
Other bacterial taxa	Abscess	27	229	11.79
Other bacterial taxa	Urinary tract tissue	2	17	11.76
Staphylococcus schleiferi	Urinary tract tissue	1	9	11.11
Serratia spp.	Respiratory	1	10	10

Serratia spp.	Other clinical specimens	2	22	9.09
Staphylococcus pseudintermedius	Uterine	2	22	9.09
Other bacterial taxa	Respiratory	16	180	8.89
Other bacterial taxa	Ear	36	426	8.45
Staphylococcus intermedius	Urinary tract tissue	1	12	8.33
Other bacterial taxa	Urine	13	160	8.12
Other bacterial taxa	Other clinical specimens	67	831	8.06
Serratia spp.	Abscess	1	13	7.69
Staphylococcus schleiferi	Uterine	1	13	7.69
Staphylococcus spp.	Urinary tract tissue	2	27	7.41
Other bacterial taxa	Uterine	2	28	7.14
Pseudomonas spp.	Ear	518	7529	6.88
Other bacterial taxa	Eye	4	59	6.78
Other bacterial taxa	Skin	41	621	6.6
Pseudomonas spp.	Respiratory	49	769	6.37
Pseudomonas spp.	Urine	66	1108	5.96
Serratia spp.	Skin	1	23	4.35
Enterobacter spp.	Eye	2	50	4
Enterobacter spp.	Urine	32	837	3.82
Pseudomonas spp.	Urinary tract tissue	4	106	3.77
Pseudomonas spp.	Uterine	2	54	3.7
Pseudomonas spp.	Anal-rectal	3	85	3.53
Pseudomonas spp.	Wound	39	1114	3.5
Klebsiella spp.	Urinary tract tissue	1	29	3.45
Citrobacter spp.	Abscess	2	65	3.08
Pseudomonas spp.	Other clinical specimens	70	2289	3.06
Pseudomonas spp.	Abscess	17	621	2.74
Staphylococcus felis	Abscess	2	74	2.7
Pseudomonas spp.	Skin	74	2982	2.48
Staphylococcus felis	Urine	10	429	2.33
Citrobacter spp.	Urine	4	182	2.2
Enterobacter spp.	Abscess	6	274	2.19
Citrobacter spp.	Ear	2	94	2.13
Klebsiella spp.	Uterine	1	48	2.08
Enterobacter spp.	Other clinical specimens	14	682	2.05
Enterobacter spp.	Respiratory	2	101	1.98
Pseudomonas spp.	Eye	5	287	1.74
Acinetobacter spp.	Urine	2	120	1.67
Acinetobacter spp.	Abscess	3	181	1.66
Acinetobacter spp.	Respiratory	2	121	1.65
Enterobacter spp.	Wound	9	586	1.54
Citrobacter spp.	Other clinical specimens	3	222	1.35
Enterobacter spp.	Skin	8	621	1.29
Enterobacter spp.	Ear	2	156	1.28
Klebsiella spp.	Anal-rectal	1	79	1.27
Staphylococcus felis	Other clinical specimens	1	82	1.22
Escherichia coli	Urinary tract tissue	2	177	1.13
Citrobacter spp.	Wound	1	122	0.82
Klebsiella spp.	Urine	16	2277	0.7
Escherichia coli	Wound	7	1019	0.69
Escherichia coli	Uterine	3	459	0.65
Escherichia coli	Respiratory	2	326	0.61
Acinetobacter spp.	Other clinical specimens	3	498	0.6
Klebsiella spp.	Other clinical specimens	4	672	0.6
Staphylococcus felis	Ear	2	354	0.56
Klebsiella spp.	Wound	2	369	0.54

Staphylococcus felis	Skin	1	190	0.53
Escherichia coli	Other clinical specimens	14	3265	0.43
Klebsiella spp.	Abscess	1	230	0.43
Klebsiella spp.	Skin	2	474	0.42
Escherichia coli	Anal-rectal	3	729	0.41
Escherichia coli	Skin	7	1743	0.4
Acinetobacter spp.	Wound	1	313	0.32
Escherichia coli	Urine	71	23490	0.3
Acinetobacter spp.	Skin	3	1073	0.28
Escherichia coli	Ear	3	1919	0.16
Escherichia coli	Abscess	0	888	0
Escherichia coli	Eye	0	71	0
Acinetobacter spp.	Anal-rectal	0	5	0
Acinetobacter spp.	Ear	0	158	0
Acinetobacter spp.	Eye	0	36	0
Acinetobacter spp.	Urinary tract tissue	0	28	0
Acinetobacter spp.	Uterine	0	60	0
Citrobacter spp.	Anal-rectal	0	19	0
Citrobacter spp.	Respiratory	0	33	0
Citrobacter spp.	Skin	0	107	0
Citrobacter spp.	Uterine	0	19	0
Enterobacter spp.	Anal-rectal	0	14	0
Enterobacter spp.	Urinary tract tissue	0	20	0
Enterobacter spp.	Uterine	0	22	0
Klebsiella spp.	Ear	0	270	0
Klebsiella spp.	Eye	0	40	0
Klebsiella spp.	Respiratory	0	161	0
Leclercia adecarboxylata	Urine	0	29	0
Leclercia adecarboxylata	Abscess	0	21	0
Leclercia adecarboxylata	Ear	0	24	0
Leclercia adecarboxylata	Eye	0	6	0
Leclercia adecarboxylata	Other clinical specimens	0	63	0
Leclercia adecarboxylata	Respiratory	0	16	0
Leclercia adecarboxylata	Skin	0	218	0
Leclercia adecarboxylata	Wound	0	90	0
Moraxella spp.	Abscess	0	19	0
Moraxella spp.	Ear	0	81	0
Moraxella spp.	Eye	0	24	0
Moraxella spp.	Other clinical specimens	0	53	0
Moraxella spp.	Respiratory	0	17	0
Moraxella spp.	Skin	0	67	0
Moraxella spp.	Wound	0	30	0
Pantoea spp.	Urine	0	150	0
Pantoea spp.	Abscess	0	95	0
Pantoea spp.	Ear	0	98	0
Pantoea spp.	Eye	0	27	0
Pantoea spp.	Other clinical specimens	0	174	0
Pantoea spp.	Respiratory	0	42	0
Pantoea spp.	Skin	0	925	0
Pantoea spp.	Uterine	0	5	0
Pantoea spp.	Wound	0	170	0
Staphylococcus felis	Eye	0	37	0
Staphylococcus felis	Respiratory	0	47	0
Staphylococcus felis	Urinary tract tissue	0	15	0
Staphylococcus felis	Wound	0	75	0
Staphylococcus intermedius	Respiratory	0	5	0

Table S12. Univariable logistic regression of beta-lactam resistance on Year, Region, Animal Category, Bacteria Group, and Sample Group, fitted separately for each retained antibiotic. Odds ratios and 95% confidence intervals were derived using the Wald (asymptotic normal) approximation.

**Antibiotic**	**Predictor**	**Odds Ratio**	**95% CI Lower**	**95% CI Upper**	**P-value**
Amoxicillin	Year: 2019	1.014	0.966	1.065	0.578
Amoxicillin	Year: 2020	1.031	0.982	1.083	0.219
Amoxicillin	Year: 2021	0.993	0.946	1.042	0.778
Amoxicillin	Year: 2022	1.012	0.964	1.062	0.641
Amoxicillin	Year: 2023	1.022	0.973	1.072	0.389
Amoxicillin	Year: 2024	1.018	0.967	1.071	0.493
Amoxicillin	Region: OK	0.962	0.925	1	0.0489
Amoxicillin	Animal Category: Avian	1.142	0.989	1.318	0.0704
Amoxicillin	Animal Category: Equine	0.643	0.588	0.704	<0.001
Amoxicillin	Animal Category: Food Animals	1.465	1.082	1.984	0.0135
Amoxicillin	Animal Category: Non-human Primates	0.912	0.794	1.048	0.194
Amoxicillin	Animal Category: Reptiles & Amphibians	2.144	1.745	2.634	<0.001
Amoxicillin	Animal Category: Zoo/Wildlife/Aquatic	1.114	0.881	1.408	0.367
Amoxicillin	Bacteria Group: *Acinetobacter spp.*	0.91	0.696	1.19	0.492
Amoxicillin	Bacteria Group: *Citrobacter spp.*	26.454	3.589	194.963	0.00131
Amoxicillin	Bacteria Group: *Enterobacter spp.*	90.553	12.615	650.017	<0.001
Amoxicillin	Bacteria Group: *Enterococcus faecalis*	0.014	0.012	0.016	0
Amoxicillin	Bacteria Group: *Enterococcus faecium*	1.942	1.774	2.126	<0.001
Amoxicillin	Bacteria Group: *Enterococcus spp.*	0.078	0.065	0.093	<0.001
Amoxicillin	Bacteria Group: *Klebsiella spp.*	85.447	65.282	111.84	<0.001
Amoxicillin	Bacteria Group: *Leclercia adecarboxylata*	3.096	2.508	3.823	<0.001
Amoxicillin	Bacteria Group: *Moraxella spp.*	0.292	0.223	0.383	<0.001
Amoxicillin	Bacteria Group: Other bacterial taxa	1.081	1.009	1.158	0.0265
Amoxicillin	Bacteria Group: *Pantoea spp.*	16.686	6.763	41.169	<0.001
Amoxicillin	Bacteria Group: *Proteus mirabilis*	0.854	0.82	0.89	<0.001
Amoxicillin	Bacteria Group: *Providencia spp.*	376.456	52.882	2679.891	<0.001
Amoxicillin	Bacteria Group: *Pseudomonas spp.*	0.763	0.171	3.41	0.723
Amoxicillin	Bacteria Group: *Serratia spp.*	27.471	3.733	202.185	0.00114
Amoxicillin	Bacteria Group: *Staphylococcus aureus*	2.376	2.183	2.585	<0.001
Amoxicillin	Bacteria Group: *Staphylococcus epidermidis*	11.145	8.637	14.38	<0.001
Amoxicillin	Bacteria Group: *Staphylococcus felis*	0.137	0.117	0.161	<0.001
Amoxicillin	Bacteria Group: *Staphylococcus intermedius*	4.717	3.612	6.16	<0.001
Amoxicillin	Bacteria Group: *Staphylococcus pseudintermedius*	5.208	5.027	5.397	0
Amoxicillin	Bacteria Group: *Staphylococcus schleiferi*	0.475	0.447	0.504	<0.001
Amoxicillin	Bacteria Group: *Staphylococcus sciuri*	2155140.11	0	80	0.741
Amoxicillin	Bacteria Group: *Staphylococcus spp.*	2.188	1.959	2.445	<0.001
Amoxicillin	Bacteria Group: *Staphylococcus xylosus*	15.354	9.968	23.651	<0.001
Amoxicillin	Bacteria Group: *Streptococcus spp.*	0.03	0.023	0.04	<0.001
Amoxicillin	Sample Group: Abscess	3.05	2.875	3.234	<0.001
Amoxicillin	Sample Group: Anal-rectal	2.753	2.519	3.009	<0.001
Amoxicillin	Sample Group: Ear	2.783	2.685	2.884	0
Amoxicillin	Sample Group: Eye	2.168	1.958	2.401	<0.001
Amoxicillin	Sample Group: Other clinical specimens	3.33	3.201	3.465	0
Amoxicillin	Sample Group: Respiratory	3.81	3.435	4.225	<0.001
Amoxicillin	Sample Group: Skin	4.411	4.261	4.566	0
Amoxicillin	Sample Group: Urinary tract tissue	2.941	2.61	3.315	<0.001

Amoxicillin	Sample Group: Uterine	2.514	2.198	2.877	<0.001
Amoxicillin	Sample Group: Wound	3.859	3.653	4.077	0
Amoxicillin/Clavulanate	Year: 2019	0.987	0.942	1.036	0.604
Amoxicillin/Clavulanate	Year: 2020	1.008	0.961	1.057	0.759
Amoxicillin/Clavulanate	Year: 2021	0.963	0.918	1.009	0.116
Amoxicillin/Clavulanate	Year: 2022	0.999	0.953	1.047	0.961
Amoxicillin/Clavulanate	Year: 2023	1.005	0.959	1.054	0.832
Amoxicillin/Clavulanate	Year: 2024	1.018	0.968	1.069	0.493
Amoxicillin/Clavulanate	Region: OK	0.941	0.905	0.977	0.0018
Amoxicillin/Clavulanate	Animal Category: Avian	1.497	1.318	1.699	<0.001
Amoxicillin/Clavulanate	Animal Category: Equine	1.199	1.1	1.308	<0.001
Amoxicillin/Clavulanate	Animal Category: Food Animals	2.412	1.844	3.155	<0.001
Amoxicillin/Clavulanate	Animal Category: Non-human Primates	1.614	1.416	1.839	<0.001
Amoxicillin/Clavulanate	Animal Category: Reptiles & Amphibians	2.407	2.051	2.826	<0.001
Amoxicillin/Clavulanate	Animal Category: Zoo/Wildlife/Aquatic	1.337	1.075	1.663	0.00918
Amoxicillin/Clavulanate	Bacteria Group: *Acinetobacter spp.*	0.183	0.124	0.271	<0.001
Amoxicillin/Clavulanate	Bacteria Group: *Citrobacter spp.*	5.001	4.24	5.899	<0.001
Amoxicillin/Clavulanate	Bacteria Group: *Enterobacter spp.*	60.949	49.208	75.492	0
Amoxicillin/Clavulanate	Bacteria Group: *Enterococcus faecalis*	0.018	0.015	0.021	0
Amoxicillin/Clavulanate	Bacteria Group: *Enterococcus faecium*	2.912	2.674	3.171	<0.001
Amoxicillin/Clavulanate	Bacteria Group: *Enterococcus spp.*	0.076	0.062	0.092	<0.001
Amoxicillin/Clavulanate	Bacteria Group: *Klebsiella spp.*	1.937	1.823	2.059	<0.001
Amoxicillin/Clavulanate	Bacteria Group: *Leclercia adecarboxylata*	4.683	3.8	5.772	<0.001
Amoxicillin/Clavulanate	Bacteria Group: *Moraxella spp.*	0.352	0.264	0.468	<0.001
Amoxicillin/Clavulanate	Bacteria Group: Other bacterial taxa	1.002	0.936	1.073	0.944
Amoxicillin/Clavulanate	Bacteria Group: *Pantoea spp.*	4.048	3.63	4.514	<0.001
Amoxicillin/Clavulanate	Bacteria Group: *Proteus mirabilis*	0.921	0.882	0.96	<0.001
Amoxicillin/Clavulanate	Bacteria Group: *Providencia spp.*	45.653	20.224	103.056	<0.001
Amoxicillin/Clavulanate	Bacteria Group: *Pseudomonas spp.*	0.528	0.107	2.615	0.434
Amoxicillin/Clavulanate	Bacteria Group: *Serratia spp.*	61.288	40.844	91.964	<0.001
Amoxicillin/Clavulanate	Bacteria Group: *Staphylococcus aureus*	0.656	0.604	0.713	<0.001
Amoxicillin/Clavulanate	Bacteria Group: *Staphylococcus epidermidis*	2.435	2.11	2.81	<0.001
Amoxicillin/Clavulanate	Bacteria Group: *Staphylococcus felis*	0.019	0.012	0.031	<0.001
Amoxicillin/Clavulanate	Bacteria Group: *Staphylococcus intermedius*	0.874	0.717	1.065	0.182
Amoxicillin/Clavulanate	Bacteria Group: *Staphylococcus pseudintermedius*	1.137	1.103	1.172	<0.001
Amoxicillin/Clavulanate	Bacteria Group: *Staphylococcus schleiferi*	0.599	0.564	0.636	<0.001
Amoxicillin/Clavulanate	Bacteria Group: *Staphylococcus sciuri*	320.623	79.993	1285.104	<0.001
Amoxicillin/Clavulanate	Bacteria Group: *Staphylococcus spp.*	1.164	1.052	1.288	0.00337
Amoxicillin/Clavulanate	Bacteria Group: *Staphylococcus xylosus*	19.997	13.506	29.608	<0.001
Amoxicillin/Clavulanate	Bacteria Group: *Streptococcus spp.*	0.01	0.006	0.018	<0.001
Amoxicillin/Clavulanate	Sample Group: Abscess	3.491	3.306	3.687	0
Amoxicillin/Clavulanate	Sample Group: Anal-rectal	4.707	4.324	5.124	<0.001
Amoxicillin/Clavulanate	Sample Group: Ear	2.806	2.705	2.91	0
Amoxicillin/Clavulanate	Sample Group: Eye	2.214	2.002	2.449	<0.001
Amoxicillin/Clavulanate	Sample Group: Other clinical specimens	4.167	4.011	4.329	0
Amoxicillin/Clavulanate	Sample Group: Respiratory	4	3.655	4.377	<0.001
Amoxicillin/Clavulanate	Sample Group: Skin	4.251	4.112	4.395	0
Amoxicillin/Clavulanate	Sample Group: Urinary tract tissue	1.797	1.593	2.027	<0.001
Amoxicillin/Clavulanate	Sample Group: Uterine	5.427	4.77	6.175	<0.001
Amoxicillin/Clavulanate	Sample Group: Wound	5.004	4.767	5.254	0
Cefalexin	Year: 2019	0.978	0.93	1.029	0.393

Cefalexin	Year: 2020	1.003	0.953	1.055	0.916
Cefalexin	Year: 2021	0.964	0.917	1.014	0.153
Cefalexin	Year: 2022	0.988	0.939	1.039	0.634
Cefalexin	Year: 2023	1.005	0.956	1.056	0.853
Cefalexin	Year: 2024	1.005	0.954	1.06	0.842
Cefalexin	Region: OK	0.926	0.889	0.965	<0.001
Cefalexin	Animal Category: Avian	1.891	1.65	2.169	<0.001
Cefalexin	Animal Category: Equine	1.155	1.057	1.261	0.00143
Cefalexin	Animal Category: Food Animals	2.468	1.851	3.291	<0.001
Cefalexin	Animal Category: Non-human Primates	3.03	2.54	3.613	<0.001
Cefalexin	Animal Category: Reptiles & Amphibians	5.2	4.252	6.359	<0.001
Cefalexin	Animal Category: Zoo/Wildlife/Aquatic	2.185	1.727	2.764	<0.001
Cefalexin	Bacteria Group: *Acinetobacter spp.*	3.514	2.736	4.514	<0.001
Cefalexin	Bacteria Group: *Citrobacter spp.*	13.537	10.842	16.904	<0.001
Cefalexin	Bacteria Group: *Enterobacter spp.*	83.236	64.933	106.699	<0.001
Cefalexin	Bacteria Group: *Enterococcus faecalis*	0	0	Inf	0.987
Cefalexin	Bacteria Group: *Klebsiella spp.*	1.984	1.867	2.108	<0.001
Cefalexin	Bacteria Group: *Leclercia adecarboxylata*	5.15	4.136	6.412	<0.001
Cefalexin	Bacteria Group:	0.441	0.336	0.578	<0.001
Cefalexin	Bacteria Group: Other bacterial taxa	1.4	1.304	1.503	<0.001
Cefalexin	Bacteria Group: *Pantoea spp.*	5.086	4.519	5.724	<0.001
Cefalexin	Bacteria Group: *Proteus mirabilis*	1.263	1.209	1.32	<0.001
Cefalexin	Bacteria Group: *Providencia spp.*	3400790.249	0	156	0.727
Cefalexin	Bacteria Group: *Pseudomonas spp.*	3.211	0.588	17.534	0.178
Cefalexin	Bacteria Group: *Serratia spp.*	222.709	105.856	468.557	<0.001
Cefalexin	Bacteria Group: *Staphylococcus aureus*	0.617	0.567	0.672	<0.001
Cefalexin	Bacteria Group: *Staphylococcus epidermidis*	2.406	2.082	2.779	<0.001
Cefalexin	Bacteria Group: *Staphylococcus felis*	0.022	0.014	0.034	<0.001
Cefalexin	Bacteria Group: *Staphylococcus intermedius*	0.872	0.714	1.064	0.178
Cefalexin	Bacteria Group: *Staphylococcus pseudintermedius*	1.119	1.085	1.154	<0.001
Cefalexin	Bacteria Group: *Staphylococcus schleiferi*	0.593	0.558	0.631	<0.001
Cefalexin	Bacteria Group: *Staphylococcus sciuri*	297.825	74.195	1195.495	<0.001
Cefalexin	Bacteria Group: *Staphylococcus spp.*	1.13	1.02	1.253	0.0197
Cefalexin	Bacteria Group: *Staphylococcus xylosus*	21.229	14.347	31.411	<0.001
Cefalexin	Bacteria Group: *Streptococcus spp.*	0.027	0.018	0.038	<0.001
Cefalexin	Sample Group: Abscess	4.064	3.831	4.31	0
Cefalexin	Sample Group: Anal-rectal	11.939	10.546	13.515	0
Cefalexin	Sample Group: Ear	2.967	2.855	3.084	0
Cefalexin	Sample Group: Eye	2.247	2.026	2.492	<0.001
Cefalexin	Sample Group: Other clinical specimens	5.074	4.867	5.289	0
Cefalexin	Sample Group: Respiratory	4.024	3.673	4.408	<0.001
Cefalexin	Sample Group: Skin	4.51	4.353	4.673	0
Cefalexin	Sample Group: Urinary tract tissue	2.052	1.806	2.331	<0.001
Cefalexin	Sample Group: Uterine	5.643	4.924	6.467	<0.001
Cefalexin	Sample Group: Wound	6.468	6.125	6.832	0
Cefovecin	Year: 2019	0.977	0.926	1.032	0.412
Cefovecin	Year: 2020	1.014	0.96	1.071	0.614
Cefovecin	Year: 2021	0.979	0.928	1.033	0.442
Cefovecin	Year: 2022	0.985	0.933	1.04	0.586
Cefovecin	Year: 2023	0.97	0.919	1.024	0.274
Cefovecin	Year: 2024	1.004	0.948	1.062	0.9

Cefovecin	Region: OK	0.838	0.801	0.877	<0.001
Cefovecin	Animal Category: Avian	0.907	0.781	1.052	0.197
Cefovecin	Animal Category: Equine	1.04	0.914	1.183	0.549
Cefovecin	Animal Category: Food Animals	1.476	1.085	2.008	0.0133
Cefovecin	Animal Category: Non-human Primates	4.891	4.122	5.805	<0.001
Cefovecin	Animal Category: Reptiles & Amphibians	0.641	0.533	0.772	<0.001
Cefovecin	Animal Category: Zoo/Wildlife/Aquatic	0.691	0.535	0.894	0.00481
Cefovecin	Bacteria Group: *Acinetobacter spp.*	16.126	14.534	17.893	0
Cefovecin	Bacteria Group: *Citrobacter spp.*	0.883	0.734	1.062	0.186
Cefovecin	Bacteria Group: *Enterobacter spp.*	2.925	2.685	3.186	<0.001
Cefovecin	Bacteria Group: *Enterococcus faecalis*	98.56	13.408	724.503	<0.001
Cefovecin	Bacteria Group: *Enterococcus faecium*	136555.611	0	3.72×10^{43}	0.793
Cefovecin	Bacteria Group: *Enterococcus spp.*	136555.611	0	7.03×10^{63}	0.864
Cefovecin	Bacteria Group: *Klebsiella spp.*	0.743	0.687	0.804	<0.001
Cefovecin	Bacteria Group: *Leclercia adecarboxylata*	0.093	0.053	0.166	<0.001
Cefovecin	Bacteria Group: *Moraxella spp.*	0.418	0.293	0.598	<0.001
Cefovecin	Bacteria Group: Other bacterial taxa	1.262	1.163	1.369	<0.001
Cefovecin	Bacteria Group: *Pantoea spp.*	0.19	0.151	0.238	<0.001
Cefovecin	Bacteria Group: *Proteus mirabilis*	0.133	0.121	0.146	0
Cefovecin	Bacteria Group: *Providencia spp.*	0.2	0.132	0.305	<0.001
Cefovecin	Bacteria Group: *Pseudomonas spp.*	5.28	1.49	18.715	0.00996
Cefovecin	Bacteria Group: *Serratia spp.*	0.208	0.157	0.275	<0.001
Cefovecin	Bacteria Group: *Staphylococcus aureus*	7.965	7.167	8.851	0
Cefovecin	Bacteria Group: *Staphylococcus epidermidis*	5.779	4.991	6.693	<0.001
Cefovecin	Bacteria Group: *Staphylococcus felis*	0.046	0.029	0.072	<0.001
Cefovecin	Bacteria Group: *Staphylococcus intermedius*	2.148	1.768	2.61	<0.001
Cefovecin	Bacteria Group: *Staphylococcus pseudintermedius*	2.51	2.428	2.594	0
Cefovecin	Bacteria Group: *Staphylococcus schleiferi*	1.357	1.276	1.445	<0.001
Cefovecin	Bacteria Group: *Staphylococcus sciuri*	651.199	162.217	2614.15	<0.001
Cefovecin	Bacteria Group: *Staphylococcus spp.*	5.059	4.513	5.671	<0.001
Cefovecin	Bacteria Group: *Staphylococcus xylosus*	93.28	49.586	175.477	<0.001
Cefovecin	Bacteria Group: *Streptococcus spp.*	0.145	0.113	0.185	<0.001
Cefovecin	Sample Group: Abscess	1.991	1.867	2.122	<0.001
Cefovecin	Sample Group: Anal-rectal	1.259	1.115	1.422	<0.001
Cefovecin	Sample Group: Ear	1.449	1.389	1.511	<0.001
Cefovecin	Sample Group: Eye	1.6	1.428	1.793	<0.001
Cefovecin	Sample Group: Other clinical specimens	2.517	2.411	2.627	0
Cefovecin	Sample Group: Respiratory	2.284	2.067	2.524	<0.001
Cefovecin	Sample Group: Skin	3.223	3.11	3.34	0
Cefovecin	Sample Group: Urinary tract tissue	1.308	1.134	1.51	<0.001
Cefovecin	Sample Group: Uterine	0.999	0.825	1.209	0.991
Cefovecin	Sample Group: Wound	2.551	2.416	2.694	<0.001
Cefpodoxime	Year: 2019	1.002	0.95	1.058	0.93
Cefpodoxime	Year: 2020	1.022	0.968	1.079	0.425
Cefpodoxime	Year: 2021	0.996	0.944	1.051	0.878
Cefpodoxime	Year: 2022	0.992	0.94	1.046	0.761
Cefpodoxime	Year: 2023	0.993	0.941	1.047	0.785
Cefpodoxime	Year: 2024	0.985	0.931	1.042	0.591
Cefpodoxime	Region: OK	0.817	0.781	0.854	<0.001
Cefpodoxime	Animal Category: Avian	0.923	0.798	1.068	0.281
Cefpodoxime	Animal Category: Equine	0.757	0.684	0.837	<0.001

Cefpodoxime	Animal Category: Food Animals	1.172	0.872	1.574	0.294
Cefpodoxime	Animal Category: Non-human Primates	3.547	3.025	4.158	<0.001
Cefpodoxime	Animal Category: Reptiles & Amphibians	0.888	0.748	1.055	0.178
Cefpodoxime	Animal Category: Zoo/Wildlife/Aquatic	0.57	0.433	0.75	<0.001
Cefpodoxime	Bacteria Group: *Acinetobacter spp.*	5.452	5.003	5.941	0
Cefpodoxime	Bacteria Group: *Citrobacter spp.*	1.265	1.083	1.478	0.00302
Cefpodoxime	Bacteria Group: *Enterobacter spp.*	14.406	13.167	15.762	0
Cefpodoxime	Bacteria Group: *Enterococcus faecalis*	0.001	0	179	0.869
Cefpodoxime	Bacteria Group: *Klebsiella spp.*	0.88	0.817	0.949	<0.001
Cefpodoxime	Bacteria Group: *Leclercia adecarboxylata*	0.032	0.012	0.085	<0.001
Cefpodoxime	Bacteria Group: *Moraxella spp.*	0.123	0.065	0.23	<0.001
Cefpodoxime	Bacteria Group: Other bacterial taxa	1.148	1.063	1.239	<0.001
Cefpodoxime	Bacteria Group: *Pantoea spp.*	0.089	0.065	0.122	<0.001
Cefpodoxime	Bacteria Group: *Proteus mirabilis*	0.132	0.12	0.145	0
Cefpodoxime	Bacteria Group: *Providencia spp.*	0.33	0.234	0.465	<0.001
Cefpodoxime	Bacteria Group: *Pseudomonas spp.*	7.282	2.192	24.189	0.00119
Cefpodoxime	Bacteria Group: *Serratia spp.*	0.294	0.154	0.56	<0.001
Cefpodoxime	Bacteria Group: *Staphylococcus aureus*	1.671	1.535	1.82	<0.001
Cefpodoxime	Bacteria Group: *Staphylococcus epidermidis*	5.636	4.877	6.514	<0.001
Cefpodoxime	Bacteria Group: *Staphylococcus felis*	0.05	0.032	0.077	<0.001
Cefpodoxime	Bacteria Group: *Staphylococcus intermedius*	2.097	1.723	2.554	<0.001
Cefpodoxime	Bacteria Group: *Staphylococcus pseudintermedius*	2.603	2.518	2.69	0
Cefpodoxime	Bacteria Group: *Staphylococcus schleiferi*	1.384	1.301	1.473	<0.001
Cefpodoxime	Bacteria Group: *Staphylococcus sciuri*	700.873	174.774	2810.62	<0.001
Cefpodoxime	Bacteria Group: *Staphylococcus spp.*	2.906	2.619	3.224	<0.001
Cefpodoxime	Bacteria Group: *Staphylococcus xylosus*	48.242	31.834	73.107	<0.001
Cefpodoxime	Bacteria Group: *Streptococcus spp.*	0.141	0.11	0.18	<0.001
Cefpodoxime	Sample Group: Abscess	1.889	1.774	2.011	<0.001
Cefpodoxime	Sample Group: Anal-rectal	1.188	1.051	1.343	0.00575
Cefpodoxime	Sample Group: Ear	1.354	1.298	1.412	<0.001
Cefpodoxime	Sample Group: Eye	1.685	1.507	1.884	<0.001
Cefpodoxime	Sample Group: Other clinical specimens	2.455	2.354	2.56	0
Cefpodoxime	Sample Group: Respiratory	2.173	1.978	2.387	<0.001
Cefpodoxime	Sample Group: Skin	2.941	2.838	3.047	0
Cefpodoxime	Sample Group: Urinary tract tissue	1.281	1.111	1.478	<0.001
Cefpodoxime	Sample Group: Uterine	0.931	0.788	1.101	0.404
Cefpodoxime	Sample Group: Wound	2.607	2.471	2.75	<0.001
Ceftazidime	Year: 2019	0.978	0.891	1.073	0.637
Ceftazidime	Year: 2020	1.024	0.933	1.123	0.623
Ceftazidime	Year: 2021	0.979	0.893	1.074	0.657
Ceftazidime	Year: 2022	0.969	0.884	1.063	0.506
Ceftazidime	Year: 2023	0.944	0.86	1.036	0.222
Ceftazidime	Year: 2024	0.925	0.839	1.02	0.119
Ceftazidime	Region: OK	0.887	0.821	0.958	0.00227
Ceftazidime	Animal Category: Avian	0.985	0.801	1.211	0.886
Ceftazidime	Animal Category: Equine	1.542	1.321	1.799	<0.001
Ceftazidime	Animal Category: Food Animals	1.171	0.741	1.853	0.499
Ceftazidime	Animal Category: Non-human Primates	1.218	0.834	1.779	0.307
Ceftazidime	Animal Category: Reptiles & Amphibians	0.68	0.532	0.871	0.00221
Ceftazidime	Animal Category: Zoo/Wildlife/Aquatic	0.741	0.506	1.085	0.124

Ceftazidime	Bacteria Group: *Acinetobacter spp.*	0.122	0.095	0.158	<0.001
Ceftazidime	Bacteria Group: *Citrobacter spp.*	0.626	0.507	0.772	<0.001
Ceftazidime	Bacteria Group: *Enterobacter spp*.	1.031	0.941	1.13	0.513
Ceftazidime	Bacteria Group: *Enterococcus spp*.	489238.491	0	2.23×10^{173}	0.947
Ceftazidime	Bacteria Group: *Klebsiella spp.*	1.084	1.004	1.171	0.0398
Ceftazidime	Bacteria Group: *Leclercia adecarboxylata*	0.051	0.021	0.122	<0.001
Ceftazidime	Bacteria Group: *Moraxella spp.*	0.095	0.042	0.213	<0.001
Ceftazidime	Bacteria Group: Other bacterial taxa	0.543	0.483	0.611	<0.001
Ceftazidime	Bacteria Group: *Pantoea spp.*	0.097	0.069	0.136	<0.001
Ceftazidime	Bacteria Group: *Proteus mirabilis*	0.146	0.132	0.163	<0.001
Ceftazidime	Bacteria Group: *Providencia spp.*	0.339	0.232	0.495	<0.001
Ceftazidime	Bacteria Group: *Pseudomonas spp.*	0.13	0.118	0.143	0
Ceftazidime	Bacteria Group: *Serratia spp.*	1.657	1.432	1.918	<0.001
Ceftazidime	Bacteria Group: *Staphylococcus felis*	0	0	2.01×10^{163}	0.959
Ceftazidime	Bacteria Group: *Staphylococcus pseudintermedius*	4.639	1.496	14.389	0.00788
Ceftazidime	Bacteria Group: *Staphylococcus schleiferi*	0	0	2.76×10^{92}	0.93
Ceftazidime	Bacteria Group: *Staphylococcus sciuri*	489238.491	0	2.23×10^{173}	0.947
Ceftazidime	Bacteria Group: *Staphylococcus spp.*	0	0	1.57×10^{114}	0.943
Ceftazidime	Sample Group: Abscess	0.92	0.821	1.03	0.146
Ceftazidime	Sample Group: Anal-rectal	1.19	1.019	1.389	0.0282
Ceftazidime	Sample Group: Ear	0.385	0.355	0.418	<0.001
Ceftazidime	Sample Group: Eye	0.479	0.353	0.649	<0.001
Ceftazidime	Sample Group: Other clinical specimens	1.014	0.949	1.084	0.671
Ceftazidime	Sample Group: Respiratory	0.765	0.658	0.889	<0.001
Ceftazidime	Sample Group: Skin	0.716	0.666	0.771	<0.001
Ceftazidime	Sample Group: Urinary tract tissue	1.006	0.76	1.331	0.966
Ceftazidime	Sample Group: Uterine	0.921	0.733	1.158	0.483
Ceftazidime	Sample Group: Wound	1.017	0.931	1.112	0.704
Imipenem	Year: 2019	1.005	0.94	1.074	0.888
Imipenem	Year: 2020	1.041	0.974	1.112	0.24
Imipenem	Year: 2021	1.006	0.942	1.074	0.863
Imipenem	Year: 2022	1.004	0.94	1.073	0.902
Imipenem	Year: 2023	1.021	0.956	1.091	0.528
Imipenem	Year: 2024	0.997	0.931	1.069	0.942
Imipenem	Region: OK	0.854	0.808	0.902	<0.001
Imipenem	Animal Category: Avian	0.251	0.186	0.337	<0.001
Imipenem	Animal Category: Equine	0.613	0.523	0.719	<0.001
Imipenem	Animal Category: Food Animals	0.805	0.55	1.178	0.265
Imipenem	Animal Category: Non-human Primates	4.172	3.538	4.918	<0.001
Imipenem	Animal Category: Reptiles & Amphibians	0.375	0.282	0.499	<0.001
Imipenem	Animal Category: Zoo/Wildlife/Aquatic	0.379	0.252	0.57	<0.001
Imipenem	Bacteria Group: *Acinetobacter spp.*	1.647	0.943	2.874	0.0793
Imipenem	Bacteria Group: *Citrobacter spp.*	4.247	2.333	7.733	<0.001
Imipenem	Bacteria Group: *Enterobacter spp.*	6.919	5.154	9.29	<0.001
Imipenem	Bacteria Group: *Enterococcus faecalis*	0	0	Inf	0.996
Imipenem	Bacteria Group: *Enterococcus faecium*	4747672654.713	0	Inf	0.987
Imipenem	Bacteria Group: *Enterococcus spp.*	4747672606.383	0	Inf	0.993
Imipenem	Bacteria Group: *Klebsiella spp.*	1.838	1.213	2.784	0.00407
Imipenem	Bacteria Group: *Leclercia adecarboxylata*	0	0	3.99×10^{89}	0.922
Imipenem	Bacteria Group: *Moraxella spp.*	0	0	4.04×10^{113}	0.938
Imipenem	Bacteria Group: Other bacterial taxa	28.639	22.838	35.913	<0.001
Imipenem	Bacteria Group: *Pantoea spp.*	0	0	1.00×10^{45}	0.853

Imipenem	Bacteria Group: *Proteus mirabilis*	235.931	86.361	644.539	<0.001
Imipenem	Bacteria Group: *Providencia spp.*	4747672597.449	0	Inf	0.99
Imipenem	Bacteria Group: *Pseudomonas spp.*	15.961	13.095	19.455	<0.001
Imipenem	Bacteria Group: *Serratia spp.*	48.813	26.948	88.42	<0.001
Imipenem	Bacteria Group: *Staphylococcus aureus*	128.528	104.714	157.756	0
Imipenem	Bacteria Group: *Staphylococcus epidermidis*	472.509	370.864	602.013	0
Imipenem	Bacteria Group: *Staphylococcus felis*	3.765	2.223	6.376	<0.001
Imipenem	Bacteria Group: *Staphylococcus intermedius*	188.147	142.62	248.208	<0.001
Imipenem	Bacteria Group: *Staphylococcus pseudintermedius*	223.788	185.663	269.743	0
Imipenem	Bacteria Group: *Staphylococcus schleiferi*	123.63	101.7	150.288	0
Imipenem	Bacteria Group: *Staphylococcus sciuri*	93125.161	12962.31	669039.338	<0.001
Imipenem	Bacteria Group: *Staphylococcus spp.*	230.894	185.966	286.675	0
Imipenem	Bacteria Group: *Staphylococcus xylosus*	4186.082	2563.712	6835.123	<0.001
Imipenem	Sample Group: Abscess	3.633	3.342	3.949	<0.001
Imipenem	Sample Group: Anal-rectal	1.419	1.145	1.759	0.00138
Imipenem	Sample Group: Ear	3.445	3.255	3.647	0
Imipenem	Sample Group: Eye	5.166	4.52	5.905	<0.001
Imipenem	Sample Group: Other clinical specimens	3.947	3.715	4.193	0
Imipenem	Sample Group: Respiratory	2.679	2.38	3.015	<0.001
Imipenem	Sample Group: Skin	7.062	6.701	7.442	0
Imipenem	Sample Group: Urinary tract tissue	2.738	2.293	3.269	<0.001
Imipenem	Sample Group: Uterine	0.782	0.561	1.091	0.148
Imipenem	Sample Group: Wound	3.876	3.603	4.171	<0.001

Table S13. Multivariable logistic regression of beta-lactam resistance, fitted separately for each retained antibiotic, adjusting simultaneously for Year, Region, Animal Category, Bacteria Group, and Sample Group. Reference categories: Year = 2018, Region = TX, Animal Category = Companion Animals, Bacteria Group = Escherichia coli, Sample Group = Urine. Benjamini–Hochberg false discovery rate (FDR) correction was applied within each antibiotic-specific model.

Antibiotic	Term	Adjusted OR	95% CI Lower	95% CI Upper	Raw P-value	FDR-adjusted P-value	Significant after FDR (q<0.05)
Amoxicillin	Year: 2019	1.041	0.978	1.107	0.206	0.247	No
Amoxicillin	Year: 2020	1.055	0.991	1.122	0.0923	0.12	No
Amoxicillin	Year: 2021	0.999	0.939	1.062	0.968	0.968	No
Amoxicillin	Year: 2022	1.018	0.957	1.082	0.578	0.603	No
Amoxicillin	Year: 2023	1.027	0.966	1.092	0.388	0.421	No
Amoxicillin	Year: 2024	1.044	0.979	1.113	0.191	0.236	No
Amoxicillin	Region: OK	0.861	0.82	0.905	<0.001	<0.001	Yes
Amoxicillin	Animal Category: Avian	0.559	0.47	0.665	<0.001	<0.001	Yes
Amoxicillin	Animal Category: Equine	0.318	0.279	0.362	<0.001	<0.001	Yes
Amoxicillin	Animal Category: Food Animals	0.772	0.537	1.108	0.16	0.202	No
Amoxicillin	Animal Category: Non-human Primates	1.095	0.891	1.346	0.386	0.421	No
Amoxicillin	Animal Category: Reptiles & Amphibians	1.267	0.979	1.64	0.072	0.096	No
Amoxicillin	Animal Category: Zoo/Wildlife/Aquatic	0.85	0.631	1.146	0.287	0.328	No
Amoxicillin	Bacteria Group: *Acinetobacter spp.*	0.313	0.238	0.412	<0.001	<0.001	Yes
Amoxicillin	Bacteria Group: *Citrobacter spp.*	13.441	1.777	101.638	0.0118	0.0167	Yes
Amoxicillin	Bacteria Group: *Enterobacter spp.*	50.8	7.016	367.844	<0.001	<0.001	Yes
Amoxicillin	Bacteria Group: *Enterococcus faecalis*	0.007	0.006	0.008	0	0	Yes
Amoxicillin	Bacteria Group: *Enterococcus faecium*	1.565	1.42	1.726	<0.001	<0.001	Yes
Amoxicillin	Bacteria Group: *Enterococcus spp.*	0.035	0.03	0.043	<0.001	<0.001	Yes
Amoxicillin	Bacteria Group: *Klebsiella spp.*	83.622	63.832	109.548	<0.001	<0.001	Yes
Amoxicillin	Bacteria Group: *Leclercia adecarboxylata*	1.1	0.883	1.369	0.395	0.421	No
Amoxicillin	Bacteria Group: *Moraxella spp.*	0.094	0.072	0.123	<0.001	<0.001	Yes
Amoxicillin	Bacteria Group: Other bacterial taxa	0.452	0.418	0.49	<0.001	<0.001	Yes
Amoxicillin	Bacteria Group: *Pantoea spp.*	5.307	2.142	13.15	<0.001	<0.001	Yes
Amoxicillin	Bacteria Group: *Proteus mirabilis*	0.665	0.636	0.696	<0.001	<0.001	Yes
Amoxicillin	Bacteria Group: *Providencia spp.*	241.614	33.874	1723.357	<0.001	<0.001	Yes

Amoxicillin	Bacteria Group: *Pseudomonas spp.*	0.397	0.084	1.875	0.243	0.285	No
Amoxicillin	Bacteria Group: *Serratia spp.*	9.223	1.244	68.355	0.0297	0.0408	Yes
Amoxicillin	Bacteria Group: *Staphylococcus aureus*	0.829	0.755	0.911	<0.001	<0.001	Yes
Amoxicillin	Bacteria Group: *Staphylococcus epidermidis*	4.008	3.092	5.195	<0.001	<0.001	Yes
Amoxicillin	Bacteria Group: *Staphylococcus felis*	0.062	0.052	0.073	<0.001	<0.001	Yes
Amoxicillin	Bacteria Group: *Staphylococcus intermedius*	2.287	1.73	3.023	<0.001	<0.001	Yes
Amoxicillin	Bacteria Group: *Staphylococcus pseudintermedius*	2.441	2.343	2.544	0	0	Yes
Amoxicillin	Bacteria Group: *Staphylococcus schleiferi*	0.164	0.153	0.175	0	0	Yes
Amoxicillin	Bacteria Group: *Staphylococcus sciuri*	849955.02	0	2.33×10^{42}	0.75	0.766	No
Amoxicillin	Bacteria Group: *Staphylococcus spp.*	0.85	0.757	0.956	0.00668	0.00972	Yes
Amoxicillin	Bacteria Group: *Staphylococcus xylosus*	7.304	4.698	11.354	<0.001	<0.001	Yes
Amoxicillin	Bacteria Group: *Streptococcus spp.*	0.01	0.008	0.013	<0.001	<0.001	Yes
Amoxicillin	Sample Group: Abscess	4.75	4.39	5.14	0	0	Yes
Amoxicillin	Sample Group: Anal-rectal	8.078	7.102	9.188	<0.001	<0.001	Yes
Amoxicillin	Sample Group: Ear	4.044	3.857	4.24	0	0	Yes
Amoxicillin	Sample Group: Eye	5.507	4.683	6.477	<0.001	<0.001	Yes
Amoxicillin	Sample Group: Other clinical specimens	5.228	4.95	5.521	0	0	Yes
Amoxicillin	Sample Group: Respiratory	5.054	4.441	5.751	<0.001	<0.001	Yes
Amoxicillin	Sample Group: Skin	5.737	5.466	6.022	0	0	Yes
Amoxicillin	Sample Group: Urinary tract tissue	3.314	2.838	3.869	<0.001	<0.001	Yes
Amoxicillin	Sample Group: Uterine	9.219	7.611	11.167	<0.001	<0.001	Yes
Amoxicillin	Sample Group: Wound	6.088	5.643	6.568	0	0	Yes
Amoxicillin/Clavulanate	Year: 2019	0.996	0.942	1.054	0.894	0.894	No
Amoxicillin/Clavulanate	Year: 2020	1.009	0.953	1.068	0.758	0.791	No
Amoxicillin/Clavulanate	Year: 2021	0.955	0.903	1.01	0.107	0.122	No
Amoxicillin/Clavulanate	Year: 2022	1.018	0.962	1.076	0.535	0.57	No

Amoxicillin/Clavulanate	Year: 2023	1.007	0.952	1.065	0.807	0.824	No
Amoxicillin/Clavulanate	Year: 2024	1.028	0.97	1.091	0.351	0.392	No
Amoxicillin/Clavulanate	Region: OK	0.828	0.791	0.867	<0.001	<0.001	Yes
Amoxicillin/Clavulanate	Animal Category: Avian	0.414	0.355	0.484	<0.001	<0.001	Yes
Amoxicillin/Clavulanate	Animal Category: Equine	0.496	0.438	0.562	<0.001	<0.001	Yes
Amoxicillin/Clavulanate	Animal Category: Food Animals	1.167	0.831	1.638	0.373	0.407	No
Amoxicillin/Clavulanate	Animal Category: Non-human Primates	4.105	3.406	4.946	<0.001	<0.001	Yes
Amoxicillin/Clavulanate	Animal Category: Reptiles & Amphibians	0.655	0.535	0.802	<0.001	<0.001	Yes
Amoxicillin/Clavulanate	Animal Category: Zoo/Wildlife/Aquatic	0.622	0.474	0.816	<0.001	<0.001	Yes
Amoxicillin/Clavulanate	Bacteria Group: *Acinetobacter spp.*	0.044	0.029	0.065	<0.001	<0.001	Yes
Amoxicillin/Clavulanate	Bacteria Group: *Citrobacter spp.*	2.356	1.962	2.829	<0.001	<0.001	Yes
Amoxicillin/Clavulanate	Bacteria Group: *Enterobacter spp.*	41.901	33.706	52.088	<0.001	<0.001	Yes
Amoxicillin/Clavulanate	Bacteria Group: *Enterococcus faecalis*	0.006	0.005	0.007	0	0	Yes
Amoxicillin/Clavulanate	Bacteria Group: *Enterococcus faecium*	2.351	2.135	2.589	<0.001	<0.001	Yes
Amoxicillin/Clavulanate	Bacteria Group: *Enterococcus spp.*	0.027	0.022	0.033	<0.001	<0.001	Yes
Amoxicillin/Clavulanate	Bacteria Group: *Klebsiella spp.*	1.482	1.382	1.588	<0.001	<0.001	Yes
Amoxicillin/Clavulanate	Bacteria Group: *Leclercia adecarboxylata*	1.276	1.023	1.592	0.0307	0.0378	Yes
Amoxicillin/Clavulanate	Bacteria Group: *Moraxella spp.*	0.085	0.064	0.113	<0.001	<0.001	Yes
Amoxicillin/Clavulanate	Bacteria Group: Other bacterial taxa	0.299	0.277	0.323	<0.001	<0.001	Yes
Amoxicillin/Clavulanate	Bacteria Group: *Pantoea spp.*	1.125	0.999	1.266	0.0524	0.0613	No
Amoxicillin/Clavulanate	Bacteria Group: *Proteus mirabilis*	0.642	0.611	0.674	<0.001	<0.001	Yes
Amoxicillin/Clavulanate	Bacteria Group: *Providencia spp.*	36.708	16.047	83.974	<0.001	<0.001	Yes
Amoxicillin/Clavulanate	Bacteria Group: *Pseudomonas spp.*	0.195	0.038	1.008	0.0511	0.0613	No
Amoxicillin/Clavulanate	Bacteria Group: *Serratia spp.*	18.407	12.194	27.787	<0.001	<0.001	Yes
Amoxicillin/Clavulanate	Bacteria Group: *Staphylococcus aureus*	0.129	0.117	0.143	0	0	Yes

Amoxicillin/Clavulanate	Bacteria Group: *Staphylococcus epidermidis*	0.628	0.541	0.729	<0.001	<0.001	Yes
Amoxicillin/Clavulanate	Bacteria Group: *Staphylococcus felis*	0.007	0.004	0.01	<0.001	<0.001	Yes
Amoxicillin/Clavulanate	Bacteria Group: *Staphylococcus intermedius*	0.265	0.215	0.326	<0.001	<0.001	Yes
Amoxicillin/Clavulanate	Bacteria Group: *Staphylococcus pseudintermedius*	0.344	0.33	0.358	0	0	Yes
Amoxicillin/Clavulanate	Bacteria Group: *Staphylococcus schleiferi*	0.15	0.14	0.161	0	0	Yes
Amoxicillin/Clavulanate	Bacteria Group: *Staphylococcus sciuri*	111.994	27.815	450.93	<0.001	<0.001	Yes
Amoxicillin/Clavulanate	Bacteria Group: *Staphylococcus spp.*	0.315	0.282	0.35	<0.001	<0.001	Yes
Amoxicillin/Clavulanate	Bacteria Group: *Staphylococcus xylosus*	6.679	4.464	9.993	<0.001	<0.001	Yes
Amoxicillin/Clavulanate	Bacteria Group: *Streptococcus spp.*	0.003	0.001	0.004	<0.001	<0.001	Yes
Amoxicillin/Clavulanate	Sample Group: Abscess	7.307	6.823	7.826	0	0	Yes
Amoxicillin/Clavulanate	Sample Group: Anal-rectal	12.05	10.761	13.492	0	0	Yes
Amoxicillin/Clavulanate	Sample Group: Ear	6.774	6.465	7.098	0	0	Yes
Amoxicillin/Clavulanate	Sample Group: Eye	7.372	6.492	8.371	<0.001	<0.001	Yes
Amoxicillin/Clavulanate	Sample Group: Other clinical specimens	8.361	7.954	8.789	0	0	Yes
Amoxicillin/Clavulanate	Sample Group: Respiratory	7.363	6.6	8.214	<0.001	<0.001	Yes
Amoxicillin/Clavulanate	Sample Group: Skin	10.572	10.098	11.068	0	0	Yes
Amoxicillin/Clavulanate	Sample Group: Urinary tract tissue	3.705	3.231	4.248	<0.001	<0.001	Yes
Amoxicillin/Clavulanate	Sample Group: Uterine	13.88	11.602	16.605	<0.001	<0.001	Yes
Amoxicillin/Clavulanate	Sample Group: Wound	10.557	9.907	11.249	0	0	Yes
Cefalexin	Year: 2019	0.989	0.932	1.049	0.711	0.778	No
Cefalexin	Year: 2020	1.013	0.955	1.075	0.669	0.778	No
Cefalexin	Year: 2021	0.962	0.907	1.02	0.195	0.256	No
Cefalexin	Year: 2022	1.004	0.947	1.065	0.882	0.922	No
Cefalexin	Year: 2023	1.003	0.946	1.063	0.928	0.949	No
Cefalexin	Year: 2024	1.013	0.953	1.077	0.679	0.778	No
Cefalexin	Region: OK	0.819	0.78	0.859	<0.001	<0.001	Yes
Cefalexin	Animal Category: Avian	0.432	0.368	0.507	<0.001	<0.001	Yes
Cefalexin	Animal Category: Equine	0.445	0.392	0.505	<0.001	<0.001	Yes
Cefalexin	Animal Category: Food Animals	0.958	0.675	1.361	0.812	0.869	No

Cefalexin	Animal Category: Non-human Primates	4.235	3.427	5.234	<0.001	<0.001	Yes
Cefalexin	Animal Category: Reptiles & Amphibians	0.888	0.702	1.122	0.32	0.408	No
Cefalexin	Animal Category: Zoo/Wildlife/Aquati c	0.906	0.681	1.206	0.5	0.622	No
Cefalexin	Bacteria Group: *Acinetobacter spp.*	0.782	0.602	1.015	0.065	0.0906	No
Cefalexin	Bacteria Group: *Citrobacter spp.*	7.315	5.755	9.299	<0.001	<0.001	Yes
Cefalexin	Bacteria Group: *Enterobacter spp.*	57.691	44.836	74.233	<0.001	<0.001	Yes
Cefalexin	Bacteria Group: *Enterococcus faecalis*	0	0	Inf	0.986	0.986	No
Cefalexin	Bacteria Group: *Klebsiella spp.*	1.482	1.381	1.591	<0.001	<0.001	Yes
Cefalexin	Bacteria Group: *Leclercia adecarboxylata*	1.211	0.958	1.53	0.109	0.148	No
Cefalexin	Bacteria Group: *Moraxella spp.*	0.091	0.069	0.12	<0.001	<0.001	Yes
Cefalexin	Bacteria Group: Other bacterial taxa	0.366	0.338	0.398	<0.001	<0.001	Yes
Cefalexin	Bacteria Group: *Pantoea spp.*	1.293	1.135	1.474	<0.001	<0.001	Yes
Cefalexin	Bacteria Group: *Proteus mirabilis*	0.913	0.867	0.962	<0.001	<0.001	Yes
Cefalexin	Bacteria Group: *Providencia spp.*	1715242.376	0	1.02×10^{39}	0.709	0.778	No
Cefalexin	Bacteria Group: *Pseudomonas spp.*	1.606	0.26	9.935	0.611	0.739	No
Cefalexin	Bacteria Group: *Serratia spp.*	59.212	28	125.218	<0.001	<0.001	Yes
Cefalexin	Bacteria Group: *Staphylococcus aureus*	0.104	0.094	0.115	0	0	Yes
Cefalexin	Bacteria Group: *Staphylococcus epidermidis*	0.524	0.45	0.609	<0.001	<0.001	Yes
Cefalexin	Bacteria Group: *Staphylococcus felis*	0.006	0.004	0.01	<0.001	<0.001	Yes
Cefalexin	Bacteria Group: *Staphylococcus intermedius*	0.227	0.184	0.28	<0.001	<0.001	Yes
Cefalexin	Bacteria Group: *Staphylococcus pseudintermedius*	0.289	0.276	0.302	0	0	Yes
Cefalexin	Bacteria Group: *Staphylococcus schleiferi*	0.127	0.119	0.137	0	0	Yes
Cefalexin	Bacteria Group: *Staphylococcus sciuri*	91.403	22.612	369.48	<0.001	<0.001	Yes
Cefalexin	Bacteria Group: *Staphylococcus spp.*	0.26	0.233	0.29	<0.001	<0.001	Yes

Cefalexin	Bacteria Group: *Staphylococcus xylosus*	6.202	4.139	9.293	<0.001	<0.001	Yes
Cefalexin	Bacteria Group: *Streptococcus spp.*	0.006	0.004	0.008	<0.001	<0.001	Yes
Cefalexin	Sample Group: Abscess	9.221	8.578	9.913	0	0	Yes
Cefalexin	Sample Group: Anal-rectal	16.368	14.342	18.68	0	0	Yes
Cefalexin	Sample Group: Ear	8.322	7.917	8.747	0	0	Yes
Cefalexin	Sample Group: Eye	9.421	8.265	10.74	<0.001	<0.001	Yes
Cefalexin	Sample Group: Other clinical specimens	10.517	9.971	11.093	0	0	Yes
Cefalexin	Sample Group: Respiratory	8.996	8.049	10.055	0	0	Yes
Cefalexin	Sample Group: Skin	13.145	12.512	13.811	0	0	Yes
Cefalexin	Sample Group: Urinary tract tissue	4.726	4.102	5.445	<0.001	<0.001	Yes
Cefalexin	Sample Group: Uterine	16.077	13.389	19.304	<0.001	<0.001	Yes
Cefalexin	Sample Group: Wound	13.76	12.854	14.73	0	0	Yes
Cefovecin	Year: 2019	0.998	0.94	1.059	0.936	0.956	No
Cefovecin	Year: 2020	1.022	0.963	1.085	0.477	0.587	No
Cefovecin	Year: 2021	0.985	0.928	1.046	0.626	0.698	No
Cefovecin	Year: 2022	1	0.942	1.061	0.994	0.994	No
Cefovecin	Year: 2023	0.977	0.921	1.037	0.446	0.564	No
Cefovecin	Year: 2024	1.02	0.958	1.086	0.535	0.628	No
Cefovecin	Region: OK	0.816	0.777	0.858	<0.001	<0.001	Yes
Cefovecin	Animal Category: Avian	0.681	0.574	0.809	<0.001	<0.001	Yes
Cefovecin	Animal Category: Equine	1.038	0.879	1.227	0.659	0.719	No
Cefovecin	Animal Category: Food Animals	0.887	0.608	1.296	0.537	0.628	No
Cefovecin	Animal Category: Non-human Primates	1.961	1.595	2.411	<0.001	<0.001	Yes
Cefovecin	Animal Category: Reptiles & Amphibians	0.532	0.423	0.669	<0.001	<0.001	Yes
Cefovecin	Animal Category: Zoo/Wildlife/Aquatic	0.44	0.324	0.6	<0.001	<0.001	Yes
Cefovecin	Bacteria Group: *Acinetobacter spp.*	11.866	10.655	13.216	0	0	Yes
Cefovecin	Bacteria Group: *Citrobacter spp.*	0.746	0.618	0.9	0.00223	0.00305	Yes
Cefovecin	Bacteria Group: *Enterobacter spp.*	2.361	2.162	2.578	<0.001	<0.001	Yes
Cefovecin	Bacteria Group: *Enterococcus faecalis*	81.075	10.947	600.475	<0.001	<0.001	Yes
Cefovecin	Bacteria Group: *Enterococcus faecium*	147767.088	0	2.41×10^{43}	0.791	0.844	No
Cefovecin	Bacteria Group: *Enterococcus spp.*	104171.817	0	1.06×10^{63}	0.865	0.903	No

Cefovecin	Bacteria Group: *Klebsiella spp.*	0.675	0.624	0.731	<0.001	<0.001	Yes
Cefovecin	Bacteria Group: *Leclercia adecarboxylata*	0.062	0.035	0.111	<0.001	<0.001	Yes
Cefovecin	Bacteria Group: *Moraxella spp.*	0.309	0.216	0.442	<0.001	<0.001	Yes
Cefovecin	Bacteria Group: Other bacterial taxa	0.978	0.897	1.066	0.608	0.695	No
Cefovecin	Bacteria Group: *Pantoea spp.*	0.126	0.1	0.158	<0.001	<0.001	Yes
Cefovecin	Bacteria Group: *Proteus mirabilis*	0.127	0.115	0.14	0	0	Yes
Cefovecin	Bacteria Group: *Providencia spp.*	0.177	0.116	0.27	<0.001	<0.001	Yes
Cefovecin	Bacteria Group: *Pseudomonas spp.*	4.219	1.176	15.128	0.0272	0.0362	Yes
Cefovecin	Bacteria Group: *Serratia spp.*	0.15	0.113	0.199	<0.001	<0.001	Yes
Cefovecin	Bacteria Group: *Staphylococcus aureus*	5.1	4.556	5.709	<0.001	<0.001	Yes
Cefovecin	Bacteria Group: *Staphylococcus epidermidis*	4.226	3.636	4.911	<0.001	<0.001	Yes
Cefovecin	Bacteria Group: *Staphylococcus felis*	0.039	0.025	0.062	<0.001	<0.001	Yes
Cefovecin	Bacteria Group: *Staphylococcus intermedius*	1.692	1.388	2.063	<0.001	<0.001	Yes
Cefovecin	Bacteria Group: *Staphylococcus pseudintermedius*	1.936	1.861	2.013	<0.001	<0.001	Yes
Cefovecin	Bacteria Group: *Staphylococcus schleiferi*	1.046	0.976	1.12	0.203	0.263	No
Cefovecin	Bacteria Group: *Staphylococcus sciuri*	507.081	126.217	2037.213	<0.001	<0.001	Yes
Cefovecin	Bacteria Group: *Staphylococcus spp.*	3.838	3.412	4.317	<0.001	<0.001	Yes
Cefovecin	Bacteria Group: *Staphylococcus xylosus*	70.724	37.526	133.292	<0.001	<0.001	Yes
Cefovecin	Bacteria Group: *Streptococcus spp.*	0.108	0.084	0.138	<0.001	<0.001	Yes
Cefovecin	Sample Group: Abscess	1.295	1.205	1.393	<0.001	<0.001	Yes
Cefovecin	Sample Group: Anal-rectal	1.412	1.241	1.606	<0.001	<0.001	Yes
Cefovecin	Sample Group: Ear	1.14	1.085	1.198	<0.001	<0.001	Yes
Cefovecin	Sample Group: Eye	1.265	1.109	1.443	<0.001	<0.001	Yes
Cefovecin	Sample Group: Other clinical specimens	1.751	1.665	1.841	<0.001	<0.001	Yes
Cefovecin	Sample Group: Respiratory	1.467	1.308	1.644	<0.001	<0.001	Yes
Cefovecin	Sample Group: Skin	2.021	1.934	2.112	<0.001	<0.001	Yes

Cefovecin	Sample Group: Urinary tract tissue	0.733	0.63	0.853	<0.001	<0.001	Yes
Cefovecin	Sample Group: Uterine	0.683	0.543	0.86	0.0012	0.00169	Yes
Cefovecin	Sample Group: Wound	1.742	1.636	1.855	<0.001	<0.001	Yes
Cefpodoxime	Year: 2019	1.02	0.962	1.082	0.508	0.6	No
Cefpodoxime	Year: 2020	1.032	0.973	1.095	0.298	0.37	No
Cefpodoxime	Year: 2021	1	0.943	1.06	0.998	0.998	No
Cefpodoxime	Year: 2022	1.011	0.953	1.072	0.72	0.808	No
Cefpodoxime	Year: 2023	0.999	0.942	1.06	0.978	0.998	No
Cefpodoxime	Year: 2024	0.997	0.937	1.061	0.924	0.966	No
Cefpodoxime	Region: OK	0.795	0.756	0.835	<0.001	<0.001	Yes
Cefpodoxime	Animal Category: Avian	0.629	0.531	0.744	<0.001	<0.001	Yes
Cefpodoxime	Animal Category: Equine	0.648	0.567	0.74	<0.001	<0.001	Yes
Cefpodoxime	Animal Category: Food Animals	0.65	0.454	0.93	0.0184	0.0242	Yes
Cefpodoxime	Animal Category: Non-human Primates	3.447	2.885	4.119	<0.001	<0.001	Yes
Cefpodoxime	Animal Category: Reptiles & Amphibians	0.718	0.586	0.88	0.00142	0.00205	Yes
Cefpodoxime	Animal Category: Zoo/Wildlife/Aquatic	0.395	0.288	0.541	<0.001	<0.001	Yes
Cefpodoxime	Bacteria Group: *Acinetobacter spp.*	3.958	3.617	4.332	<0.001	<0.001	Yes
Cefpodoxime	Bacteria Group: *Citrobacter spp.*	1.045	0.89	1.226	0.59	0.678	No
Cefpodoxime	Bacteria Group: *Enterobacter spp.*	12.049	10.992	13.208	0	0	Yes
Cefpodoxime	Bacteria Group: *Enterococcus faecalis*	0.001	0	1.41×10^{34}	0.864	0.947	No
Cefpodoxime	Bacteria Group: *Klebsiella spp.*	0.803	0.744	0.866	<0.001	<0.001	Yes
Cefpodoxime	Bacteria Group: *Leclercia adecarboxylata*	0.021	0.008	0.057	<0.001	<0.001	Yes
Cefpodoxime	Bacteria Group: *Moraxella spp.*	0.09	0.048	0.169	<0.001	<0.001	Yes
Cefpodoxime	Bacteria Group: Other bacterial taxa	0.931	0.857	1.011	0.0891	0.114	No
Cefpodoxime	Bacteria Group: *Pantoea spp.*	0.06	0.044	0.082	<0.001	<0.001	Yes
Cefpodoxime	Bacteria Group: *Proteus mirabilis*	0.127	0.115	0.14	0	0	Yes
Cefpodoxime	Bacteria Group: *Providencia spp.*	0.281	0.198	0.397	<0.001	<0.001	Yes
Cefpodoxime	Bacteria Group: *Pseudomonas spp.*	5.94	1.775	19.881	0.00385	0.00536	Yes
Cefpodoxime	Bacteria Group: *Serratia spp.*	0.206	0.108	0.393	<0.001	<0.001	Yes
Cefpodoxime	Bacteria Group: *Staphylococcus aureus*	0.951	0.864	1.047	0.306	0.371	No

Cefpodoxime	Bacteria Group: *Staphylococcus epidermidis*	4.062	3.501	4.713	<0.001	<0.001	Yes
Cefpodoxime	Bacteria Group: *Staphylococcus felis*	0.043	0.027	0.066	<0.001	<0.001	Yes
Cefpodoxime	Bacteria Group: *Staphylococcus intermedius*	1.68	1.375	2.052	<0.001	<0.001	Yes
Cefpodoxime	Bacteria Group: *Staphylococcus pseudintermedius*	2.022	1.945	2.103	<0.001	<0.001	Yes
Cefpodoxime	Bacteria Group: *Staphylococcus schleiferi*	1.089	1.017	1.167	0.0147	0.0198	Yes
Cefpodoxime	Bacteria Group: *Staphylococcus sciuri*	562.18	140.083	2256.136	<0.001	<0.001	Yes
Cefpodoxime	Bacteria Group: *Staphylococcus spp.*	2.232	2.003	2.487	<0.001	<0.001	Yes
Cefpodoxime	Bacteria Group: *Staphylococcus xylosus*	41.124	27.02	62.589	<0.001	<0.001	Yes
Cefpodoxime	Bacteria Group: *Streptococcus spp.*	0.106	0.083	0.136	<0.001	<0.001	Yes
Cefpodoxime	Sample Group: Abscess	1.294	1.205	1.39	<0.001	<0.001	Yes
Cefpodoxime	Sample Group: Anal-rectal	1.35	1.185	1.538	<0.001	<0.001	Yes
Cefpodoxime	Sample Group: Ear	1.11	1.056	1.167	<0.001	<0.001	Yes
Cefpodoxime	Sample Group: Eye	1.384	1.217	1.575	<0.001	<0.001	Yes
Cefpodoxime	Sample Group: Other clinical specimens	1.828	1.74	1.92	<0.001	<0.001	Yes
Cefpodoxime	Sample Group: Respiratory	1.68	1.51	1.868	<0.001	<0.001	Yes
Cefpodoxime	Sample Group: Skin	1.982	1.897	2.071	<0.001	<0.001	Yes
Cefpodoxime	Sample Group: Urinary tract tissue	0.756	0.651	0.878	<0.001	<0.001	Yes
Cefpodoxime	Sample Group: Uterine	1.01	0.83	1.23	0.919	0.966	No
Cefpodoxime	Sample Group: Wound	1.752	1.647	1.865	<0.001	<0.001	Yes
Ceftazidime	Year: 2019	0.966	0.878	1.064	0.486	0.642	No
Ceftazidime	Year: 2020	0.997	0.905	1.097	0.946	0.959	No
Ceftazidime	Year: 2021	0.962	0.874	1.058	0.426	0.583	No
Ceftazidime	Year: 2022	0.956	0.869	1.052	0.357	0.505	No
Ceftazidime	Year: 2023	0.926	0.841	1.019	0.117	0.172	No
Ceftazidime	Year: 2024	0.91	0.823	1.007	0.0682	0.104	No
Ceftazidime	Region: OK	0.879	0.812	0.953	0.00166	0.00341	Yes
Ceftazidime	Animal Category: Avian	0.706	0.568	0.877	0.00167	0.00341	Yes
Ceftazidime	Animal Category: Equine	1.552	1.295	1.862	<0.001	<0.001	Yes
Ceftazidime	Animal Category: Food Animals	1.095	0.677	1.77	0.712	0.884	No
Ceftazidime	Animal Category: Non-human Primates	0.877	0.593	1.297	0.51	0.653	No

Ceftazidime	Animal Category: Reptiles & Amphibians	0.643	0.496	0.835	<0.001	0.00203	Yes
Ceftazidime	Animal Category: Zoo/Wildlife/Aquatic	0.621	0.417	0.924	0.0187	0.0306	Yes
Ceftazidime	Bacteria Group: *Acinetobacter spp.*	0.094	0.073	0.121	<0.001	<0.001	Yes
Ceftazidime	Bacteria Group: *Citrobacter spp.*	0.549	0.443	0.679	<0.001	<0.001	Yes
Ceftazidime	Bacteria Group: *Enterobacter spp.*	0.861	0.782	0.948	0.0023	0.00449	Yes
Ceftazidime	Bacteria Group: *Enterococcus spp.*	714142.864	0	3.26×10^{173}	0.945	0.959	No
Ceftazidime	Bacteria Group: *Klebsiella spp.*	1.006	0.93	1.088	0.881	0.959	No
Ceftazidime	Bacteria Group: *Leclercia adecarboxylata*	0.038	0.016	0.092	<0.001	<0.001	Yes
Ceftazidime	Bacteria Group: *Moraxella spp.*	0.077	0.034	0.174	<0.001	<0.001	Yes
Ceftazidime	Bacteria Group: Other bacterial taxa	0.441	0.39	0.5	<0.001	<0.001	Yes
Ceftazidime	Bacteria Group: *Pantoea spp.*	0.074	0.052	0.104	<0.001	<0.001	Yes
Ceftazidime	Bacteria Group: *Proteus mirabilis*	0.142	0.128	0.158	<0.001	<0.001	Yes
Ceftazidime	Bacteria Group: *Providencia spp.*	0.31	0.212	0.454	<0.001	<0.001	Yes
Ceftazidime	Bacteria Group: *Pseudomonas spp.*	0.112	0.101	0.125	0	0	Yes
Ceftazidime	Bacteria Group: *Serratia spp.*	1.315	1.13	1.53	<0.001	<0.001	Yes
Ceftazidime	Bacteria Group: *Staphylococcus felis*	0	0	1.97×10^{163}	0.959	0.959	No
Ceftazidime	Bacteria Group: *Staphylococcus pseudintermedius*	4.151	1.329	12.964	0.0143	0.0247	Yes
Ceftazidime	Bacteria Group: *Staphylococcus schleiferi*	0	0	2.75×10^{92}	0.93	0.959	No
Ceftazidime	Bacteria Group: *Staphylococcus sciuri*	625796.366	0	2.86×10^{173}	0.946	0.959	No
Ceftazidime	Bacteria Group: *Staphylococcus spp.*	0	0	1.74×10^{114}	0.943	0.959	No
Ceftazidime	Sample Group: Abscess	1.281	1.136	1.444	<0.001	<0.001	Yes
Ceftazidime	Sample Group: Anal-rectal	1.474	1.256	1.731	<0.001	<0.001	Yes
Ceftazidime	Sample Group: Ear	1.119	1.023	1.224	0.0145	0.0247	Yes
Ceftazidime	Sample Group: Eye	1.018	0.742	1.397	0.912	0.959	No
Ceftazidime	Sample Group: Other clinical specimens	1.473	1.367	1.587	<0.001	<0.001	Yes
Ceftazidime	Sample Group: Respiratory	1.446	1.23	1.701	<0.001	<0.001	Yes
Ceftazidime	Sample Group: Skin	1.581	1.456	1.716	<0.001	<0.001	Yes

Ceftazidime	Sample Group: Urinary tract tissue	1.323	0.987	1.773	0.0608	0.0959	No
Ceftazidime	Sample Group: Uterine	0.715	0.552	0.925	0.0106	0.0197	Yes
Ceftazidime	Sample Group: Wound	1.547	1.403	1.706	<0.001	<0.001	Yes
Imipenem	Year: 2019	1.031	0.953	1.115	0.452	0.574	No
Imipenem	Year: 2020	1.062	0.982	1.15	0.133	0.195	No
Imipenem	Year: 2021	1.037	0.959	1.121	0.36	0.47	No
Imipenem	Year: 2022	1.047	0.968	1.133	0.247	0.342	No
Imipenem	Year: 2023	1.048	0.969	1.133	0.243	0.342	No
Imipenem	Year: 2024	1.043	0.961	1.133	0.315	0.423	No
Imipenem	Region: OK	0.813	0.762	0.867	<0.001	<0.001	Yes
Imipenem	Animal Category: Avian	0.627	0.433	0.908	0.0135	0.0245	Yes
Imipenem	Animal Category: Equine	0.808	0.627	1.042	0.0999	0.151	No
Imipenem	Animal Category: Food Animals	0.607	0.352	1.047	0.0725	0.114	No
Imipenem	Animal Category: Non-human Primates	5.136	4.141	6.37	<0.001	<0.001	Yes
Imipenem	Animal Category: Reptiles & Amphibians	0.908	0.619	1.333	0.622	0.75	No
Imipenem	Animal Category: Zoo/Wildlife/Aquatic	0.539	0.307	0.946	0.0312	0.0505	No
Imipenem	Bacteria Group: *Acinetobacter spp.*	1.191	0.681	2.081	0.54	0.668	No
Imipenem	Bacteria Group: *Citrobacter spp.*	3.35	1.833	6.122	<0.001	<0.001	Yes
Imipenem	Bacteria Group: *Enterobacter spp.*	5.541	4.12	7.453	<0.001	<0.001	Yes
Imipenem	Bacteria Group: *Enterococcus faecalis*	0	0	Inf	0.996	0.996	No
Imipenem	Bacteria Group: *Enterococcus faecium*	4542380265.335	0	Inf	0.987	0.996	No
Imipenem	Bacteria Group: *Enterococcus spp.*	5502085851.46	0	Inf	0.993	0.996	No
Imipenem	Bacteria Group: *Klebsiella spp.*	1.673	1.104	2.536	0.0152	0.0256	Yes
Imipenem	Bacteria Group: *Leclercia adecarboxylata*	0	0	1.03×10^{89}	0.919	0.996	No
Imipenem	Bacteria Group: *Moraxella spp.*	0	0	9.87×10^{112}	0.936	0.996	No
Imipenem	Bacteria Group: Other bacterial taxa	22.114	17.558	27.852	<0.001	<0.001	Yes
Imipenem	Bacteria Group: *Pantoea spp.*	0	0	3.92×10^{44}	0.847	0.971	No
Imipenem	Bacteria Group: *Proteus mirabilis*	233.959	85.275	641.886	<0.001	<0.001	Yes
Imipenem	Bacteria Group: *Providencia spp.*	4364728437.079	0	Inf	0.99	0.996	No
Imipenem	Bacteria Group: *Pseudomonas spp.*	13.116	10.728	16.035	<0.001	<0.001	Yes

Imipenem	Bacteria Group: *Serratia spp.*	38.696	21.276	70.381	<0.001	<0.001	Yes
Imipenem	Bacteria Group: *Staphylococcus aureus*	73.779	59.668	91.229	0	0	Yes
Imipenem	Bacteria Group: *Staphylococcus epidermidis*	366.996	286.831	469.565	0	0	Yes
Imipenem	Bacteria Group: *Staphylococcus felis*	3.355	1.979	5.685	<0.001	<0.001	Yes
Imipenem	Bacteria Group: *Staphylococcus intermedius*	154.69	116.869	204.752	<0.001	<0.001	Yes
Imipenem	Bacteria Group: *Staphylococcus pseudintermedius*	178.846	148.059	216.035	0	0	Yes
Imipenem	Bacteria Group: *Staphylococcus schleiferi*	97.547	79.939	119.033	0	0	Yes
Imipenem	Bacteria Group: *Staphylococcus sciuri*	78237.089	10879.877	562602.147	<0.001	<0.001	Yes
Imipenem	Bacteria Group: *Staphylococcus spp.*	186.336	149.534	232.195	0	0	Yes
Imipenem	Bacteria Group: *Staphylococcus xylosus*	3575.004	2172.362	5883.299	<0.001	<0.001	Yes
Imipenem	Sample Group: Abscess	1.178	1.067	1.3	0.00114	0.00215	Yes
Imipenem	Sample Group: Anal-rectal	1.381	1.064	1.791	0.0152	0.0256	Yes
Imipenem	Sample Group: Ear	1.198	1.12	1.282	<0.001	<0.001	Yes
Imipenem	Sample Group: Eye	1.517	1.297	1.775	<0.001	<0.001	Yes
Imipenem	Sample Group: Other clinical specimens	1.713	1.589	1.846	<0.001	<0.001	Yes
Imipenem	Sample Group: Respiratory	1.447	1.253	1.67	<0.001	<0.001	Yes
Imipenem	Sample Group: Skin	1.935	1.818	2.059	<0.001	<0.001	Yes
Imipenem	Sample Group: Urinary tract tissue	0.588	0.486	0.711	<0.001	<0.001	Yes
Imipenem	Sample Group: Uterine	1.077	0.678	1.712	0.753	0.885	No
Imipenem	Sample Group: Wound	1.586	1.452	1.732	<0.001	<0.001	Yes

Table S14. Sample-size-standardized bacterial diversity by Animal Category × Antimicrobial. Each group was rarefied to n = 164 interpretable binary susceptibility observations using 1,000 random subsamples without replacement. Diversity was calculated from the original bacterial taxa represented in the binary susceptibility dataset.

Animal Category	Antimicrobial	Original n	Observed taxa	Standardized n	Rarefied richness mean (95% CI)	Rarefied Shannon mean (95% CI)	Rarefied Simpson mean	Rarefied Pielou mean
Avian	Amoxicillin	779	48	164	26.45 (22.00–31.00)	2.311 (2.105–2.499)	0.814	0.707
Avian	Amoxicillin/Clavulanate	963	52	164	28.49 (24.00–34.00)	2.541 (2.327–2.734)	0.861	0.760
Avian	Cefalexin	850	46	164	26.46 (21.00–31.00)	2.400 (2.197–2.589)	0.840	0.734
Avian	Cefovecin	889	49	164	27.33 (23.00–32.00)	2.445 (2.264–2.631)	0.844	0.740
Avian	Cefpodoxime	926	50	164	26.93 (22.00–32.00)	2.445 (2.240–2.637)	0.849	0.744
Avian	Ceftazidime	928	43	164	22.15 (17.00–27.00)	2.314 (2.134–2.480)	0.847	0.749
Avian	Imipenem	923	46	164	24.41 (20.00–30.00)	2.351 (2.156–2.542)	0.843	0.737
Companion Animals	Amoxicillin	115889	108	164	18.08 (14.00–23.00)	2.015 (1.814–2.199)	0.801	0.698
Companion Animals	Amoxicillin/Clavulanate	126571	116	164	20.60 (16.98–25.00)	2.171 (1.962–2.372)	0.823	0.719
Companion Animals	Cefalexin	107188	110	164	18.53 (14.97–23.00)	1.943 (1.729–2.147)	0.777	0.667
Companion Animals	Cefovecin	110790	111	164	19.29 (15.00–24.00)	1.958 (1.755–2.176)	0.780	0.663
Companion Animals	Cefpodoxime	112528	108	164	19.17 (15.00–24.00)	1.983 (1.763–2.192)	0.786	0.673
Companion Animals	Ceftazidime	77232	95	164	14.74 (11.00–19.00)	1.755 (1.581–1.938)	0.748	0.655
Companion Animals	Imipenem	108788	104	164	16.98 (13.00–22.00)	1.899 (1.706–2.086)	0.780	0.673
Equine	Amoxicillin	1949	51	164	25.17 (21.00–30.00)	2.494 (2.301–2.670)	0.871	0.774
Equine	Amoxicillin/Clavulanate	2139	52	164	27.44 (23.00–32.00)	2.655 (2.471–2.819)	0.890	0.802

Equine	Cefalexin	2016	48	164	24.68 (20.00–29.00)	2.521 (2.361–2.698)	0.876	0.788
Equine	Cefovecin	1118	50	164	26.52 (22.00–31.00)	2.359 (2.157–2.566)	0.827	0.721
Equine	Cefpodoxime	2119	51	164	26.30 (22.00–31.00)	2.622 (2.451–2.794)	0.888	0.803
Equine	Ceftazidime	1210	44	164	22.42 (18.00–27.00)	2.301 (2.125–2.484)	0.842	0.741
Equine	Imipenem	1512	51	164	26.20 (22.00–31.00)	2.585 (2.410–2.765)	0.882	0.793
Food Animals	Amoxicillin	185	27	164	25.84 (24.00–27.00)	2.620 (2.556–2.674)	0.897	0.806
Food Animals	Amoxicillin/Clavulanate	221	29	164	26.40 (24.00–29.00)	2.756 (2.668–2.831)	0.915	0.842
Food Animals	Cefalexin	203	25	164	23.55 (21.00–25.00)	2.625 (2.552–2.691)	0.904	0.831
Food Animals	Cefovecin	173	25	164	24.68 (23.00–25.00)	2.725 (2.685–2.759)	0.909	0.850
Food Animals	Cefpodoxime	202	25	164	23.77 (22.00–25.00)	2.678 (2.609–2.739)	0.908	0.846
Food Animals	Ceftazidime	164	20	164	20.00 (20.00–20.00)	2.321 (2.321–2.321)	0.861	0.775
Food Animals	Imipenem	215	25	164	22.95 (20.00–25.00)	2.586 (2.504–2.663)	0.900	0.826
Non-human Primates	Amoxicillin	812	27	164	14.69 (11.00–18.00)	1.595 (1.409–1.769)	0.696	0.595
Non-human Primates	Amoxicillin/Clavulanate	906	31	164	17.09 (13.00–21.00)	1.725 (1.521–1.922)	0.717	0.609
Non-human Primates	Cefalexin	582	28	164	16.10 (13.00–20.00)	1.531 (1.340–1.725)	0.606	0.552
Non-human Primates	Cefovecin	593	32	164	19.81 (16.00–24.00)	1.731 (1.506–1.925)	0.651	0.581
Non-human Primates	Cefpodoxime	631	30	164	17.28 (14.00–21.00)	1.573 (1.377–1.777)	0.609	0.553
Non-human Primates	Ceftazidime	234	23	164	19.79 (17.00–23.00)	2.174 (2.060–2.286)	0.820	0.729
Non-human Primates	Imipenem	575	26	164	15.04 (12.00–19.00)	1.571 (1.376–1.763)	0.638	0.581

Reptiles & Amphibians	Amoxicillin	467	51	164	33.65 (29.00–39.00)	2.840 (2.679–2.993)	0.912	0.808
Reptiles & Amphibians	Amoxicillin/Clavulanate	623	53	164	32.16 (27.00–37.00)	2.871 (2.710–3.009)	0.919	0.828
Reptiles & Amphibians	Cefalexin	580	53	164	30.23 (25.00–36.00)	2.706 (2.545–2.865)	0.904	0.795
Reptiles & Amphibians	Cefovecin	680	54	164	30.08 (25.00–35.00)	2.826 (2.672–2.978)	0.920	0.831
Reptiles & Amphibians	Cefpodoxime	669	56	164	31.55 (26.00–37.02)	2.807 (2.640–2.974)	0.915	0.814
Reptiles & Amphibians	Ceftazidime	878	56	164	27.29 (22.00–33.00)	2.730 (2.583–2.875)	0.910	0.827
Reptiles & Amphibians	Imipenem	701	50	164	28.59 (24.00–34.00)	2.592 (2.430–2.763)	0.885	0.774
Zoo/Wildlife/Aquatic	Amoxicillin	290	46	164	37.60 (33.00–42.00)	2.993 (2.851–3.122)	0.911	0.825
Zoo/Wildlife/Aquatic	Amoxicillin/Clavulanate	327	52	164	40.64 (36.00–46.00)	3.102 (2.946–3.253)	0.922	0.838
Zoo/Wildlife/Aquatic	Cefalexin	295	48	164	38.64 (34.00–43.00)	2.995 (2.845–3.131)	0.909	0.820
Zoo/Wildlife/Aquatic	Cefovecin	341	49	164	37.98 (33.00–43.00)	3.001 (2.852–3.143)	0.917	0.825
Zoo/Wildlife/Aquatic	Cefpodoxime	336	48	164	36.91 (32.00–42.00)	2.979 (2.836–3.130)	0.916	0.826
Zoo/Wildlife/Aquatic	Ceftazidime	341	42	164	32.07 (28.00–36.02)	2.816 (2.668–2.958)	0.908	0.813
Zoo/Wildlife/Aquatic	Imipenem	340	46	164	35.34 (31.00–40.00)	2.861 (2.700–3.014)	0.904	0.803

Table S15. Top 20 Bacteria × Specimen × Antibiotic combinations ranked by Resistance Burden Score ([resistance prevalence % / 100] × number of resistant observations), among combinations with at least 5 Total Isolates (n).

Rank	Bacteria_Group	Sample_Group	Antibiotic	Resistant (n)	Total Isolates (n)	Resistance (%)	Resistance Burden Score
1	*Staphylococcus pseudintermedius*	Skin	Amoxicillin	10674	12432	85.86	9164.6
2	*Staphylococcus pseudintermedius*	Ear	Amoxicillin	5746	7239	79.38	4560.92
3	*Staphylococcus pseudintermedius*	Urine	Amoxicillin	4665	5608	83.18	3880.57
4	*Staphylococcus pseudintermedius*	Skin	Amoxicillin/Clavulanate	6370	12761	49.92	3179.76
5	*Staphylococcus pseudintermedius*	Skin	Cefpodoxime	6337	12714	49.84	3158.53
6	*Staphylococcus pseudintermedius*	Skin	Cefovecin	6301	12669	49.74	3133.84
7	*Staphylococcus pseudintermedius*	Skin	Cefalexin	6108	12362	49.41	3017.93
8	*Staphylococcus pseudintermedius*	Other clinical specimens	Amoxicillin	3477	4027	86.34	3002.12
9	*Staphylococcus pseudintermedius*	Skin	Imipenem	5925	11763	50.37	2984.41
10	*Escherichia coli*	Urine	Amoxicillin	7417	22387	33.13	2457.31
11	*Klebsiella spp.*	Urine	Amoxicillin	2212	2255	98.09	2169.82
12	*Escherichia coli*	Other clinical specimens	Amoxicillin	2497	3216	77.64	1938.75
13	*Proteus mirabilis*	Ear	Cefalexin	1981	2236	88.6	1755.08
14	*Staphylococcus pseudintermedius*	Wound	Amoxicillin	2005	2409	83.23	1668.75
15	*Escherichia coli*	Other clinical specimens	Cefalexin	2280	3185	71.59	1632.15
16	*Escherichia coli*	Other clinical specimens	Amoxicillin/Clavulanate	2286	3202	71.39	1632.04
17	*Proteus mirabilis*	Ear	Amoxicillin	2078	2692	77.19	1604.04
18	*Proteus mirabilis*	Ear	Amoxicillin/Clavulanate	2016	2684	75.11	1514.25
19	*Escherichia coli*	Skin	Amoxicillin	1553	1727	89.92	1396.53
20	*Escherichia coli*	Ear	Amoxicillin	1580	1872	84.4	1333.55

Supplementary Figures

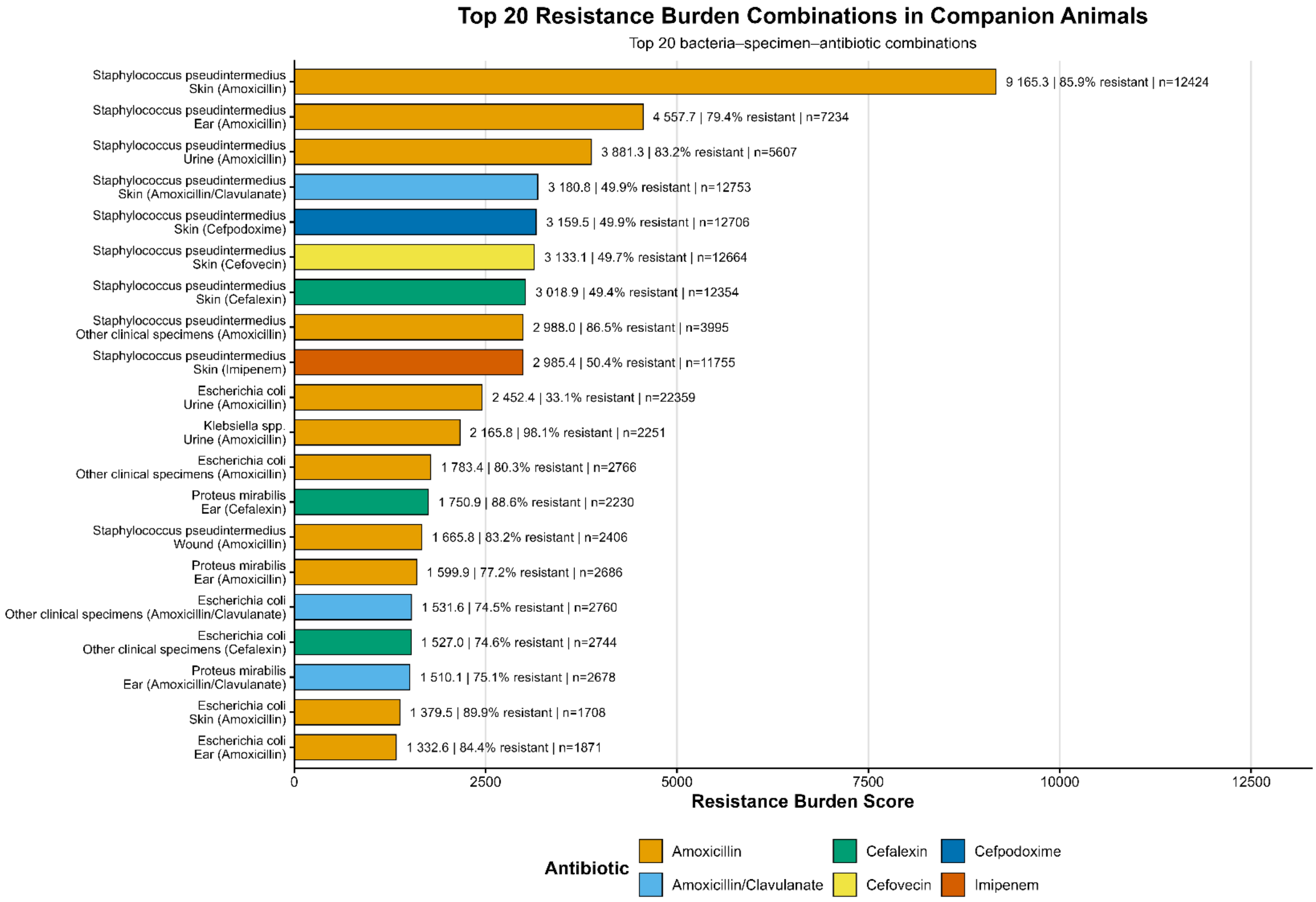

Figure S1. Top 20 bacterial group–clinical specimen group–antimicrobial combinations ranked by Resistance Burden Score among companion animals.

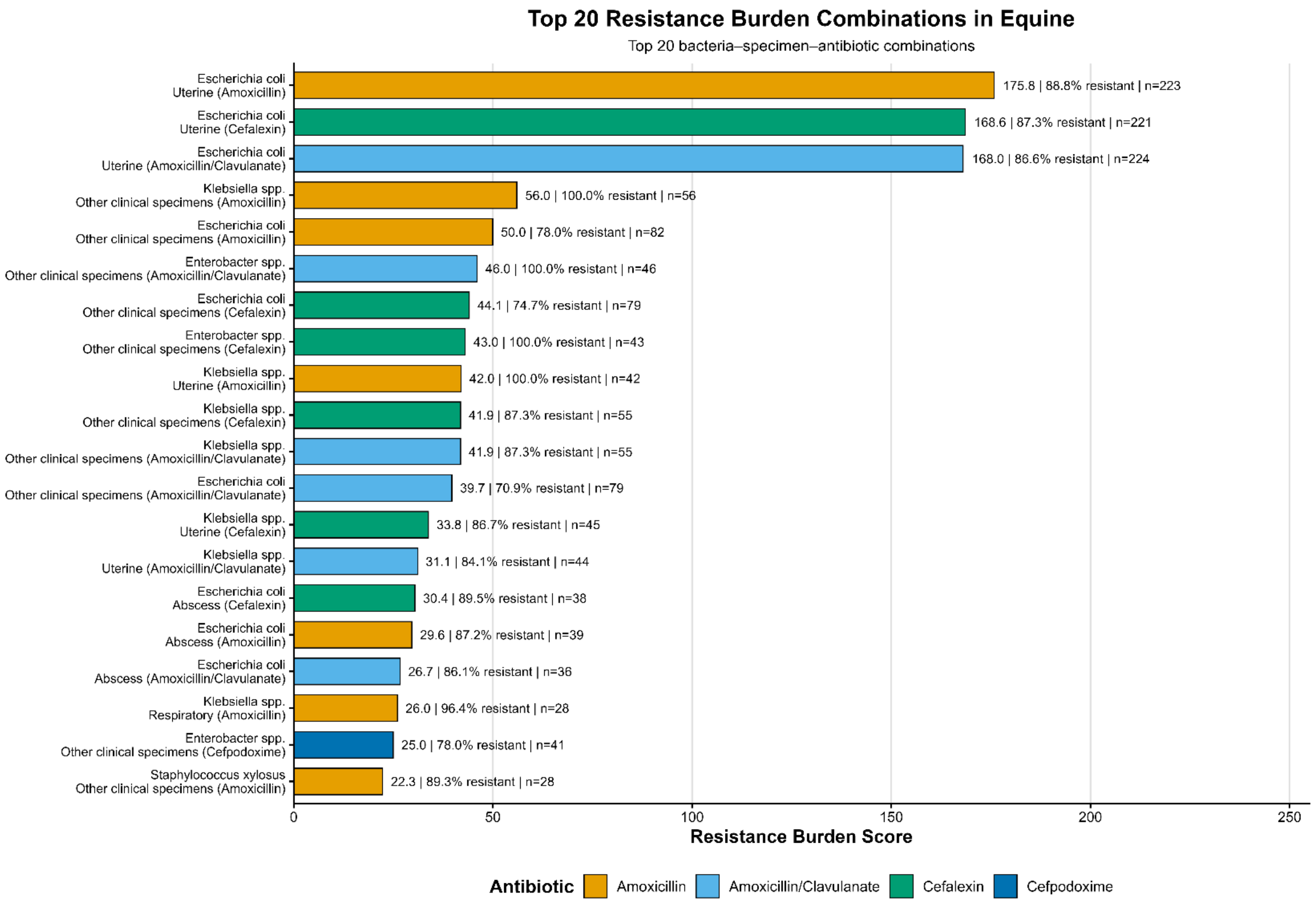

Figure S2. Top 20 bacterial group–clinical specimen group–antimicrobial combinations ranked by Resistance Burden Score among equine isolates.

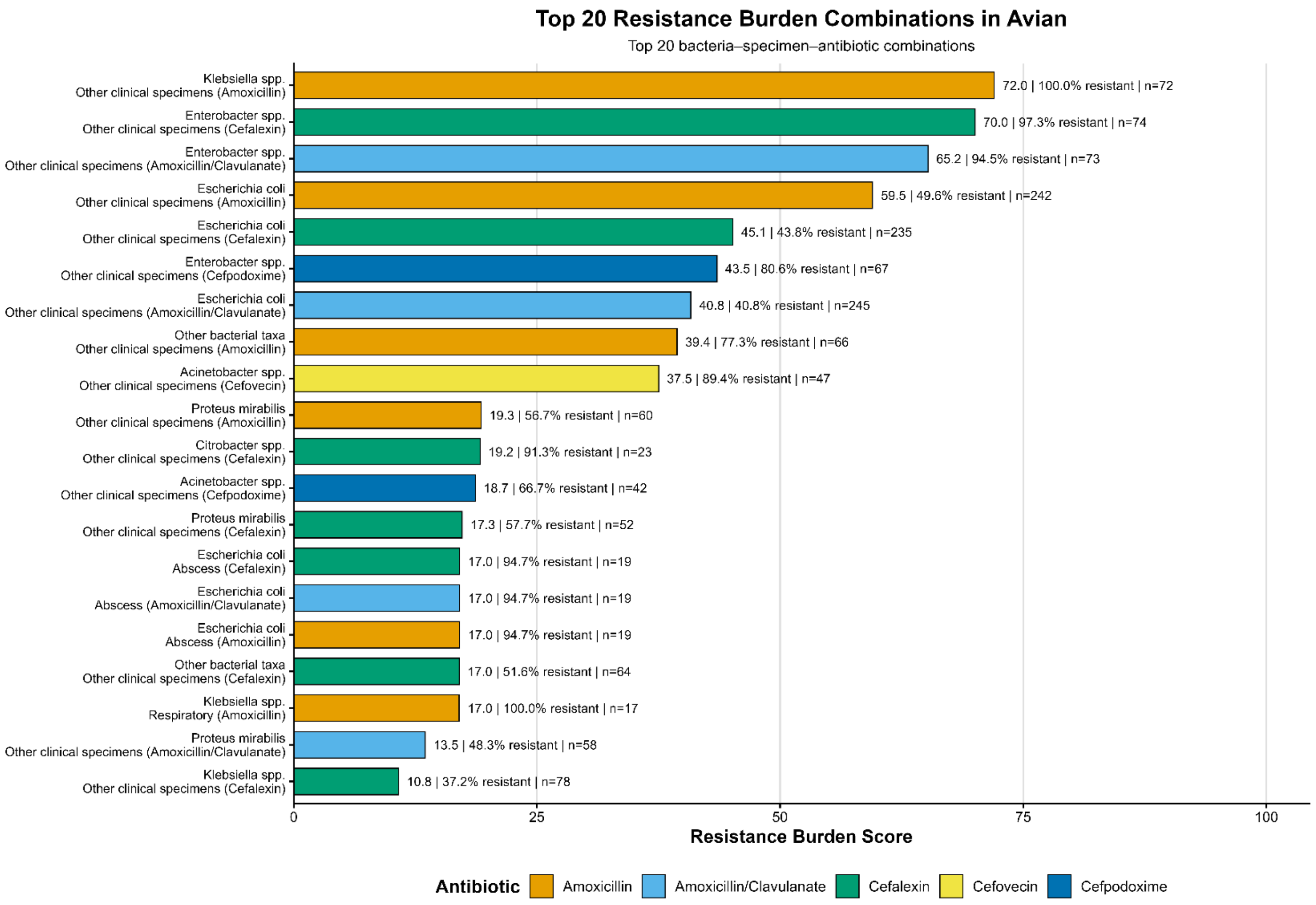

Figure S3. Top 20 bacterial group–clinical specimen group–antimicrobial combinations ranked by Resistance Burden Score among avian isolates.

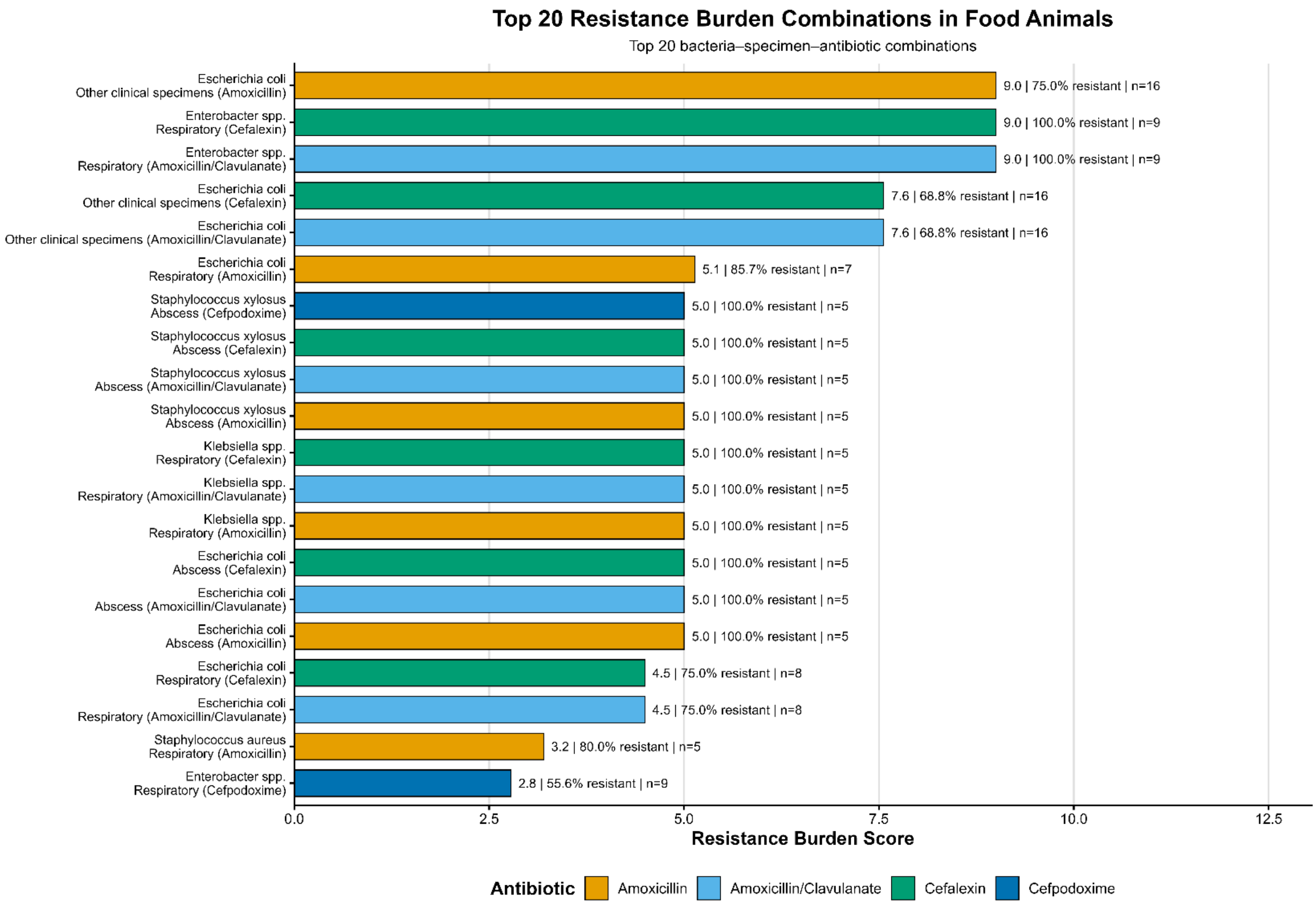

Figure S4. Top 20 bacterial group–clinical specimen group–antimicrobial combinations ranked by Resistance Burden Score among food-animal isolates.

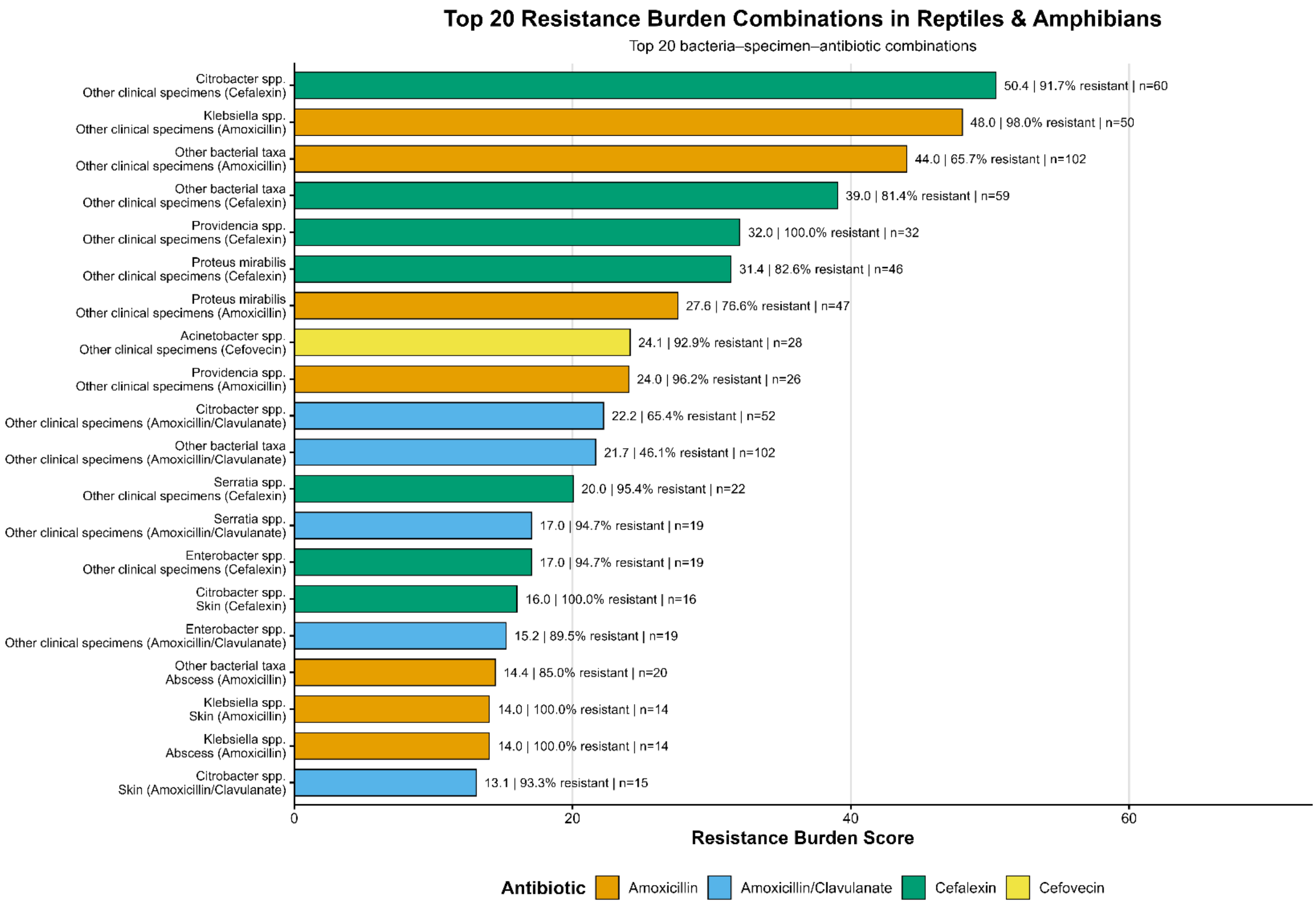

Figure S5. Top 20 bacterial group–clinical specimen group–antimicrobial combinations ranked by Resistance Burden Score among reptiles and amphibians.

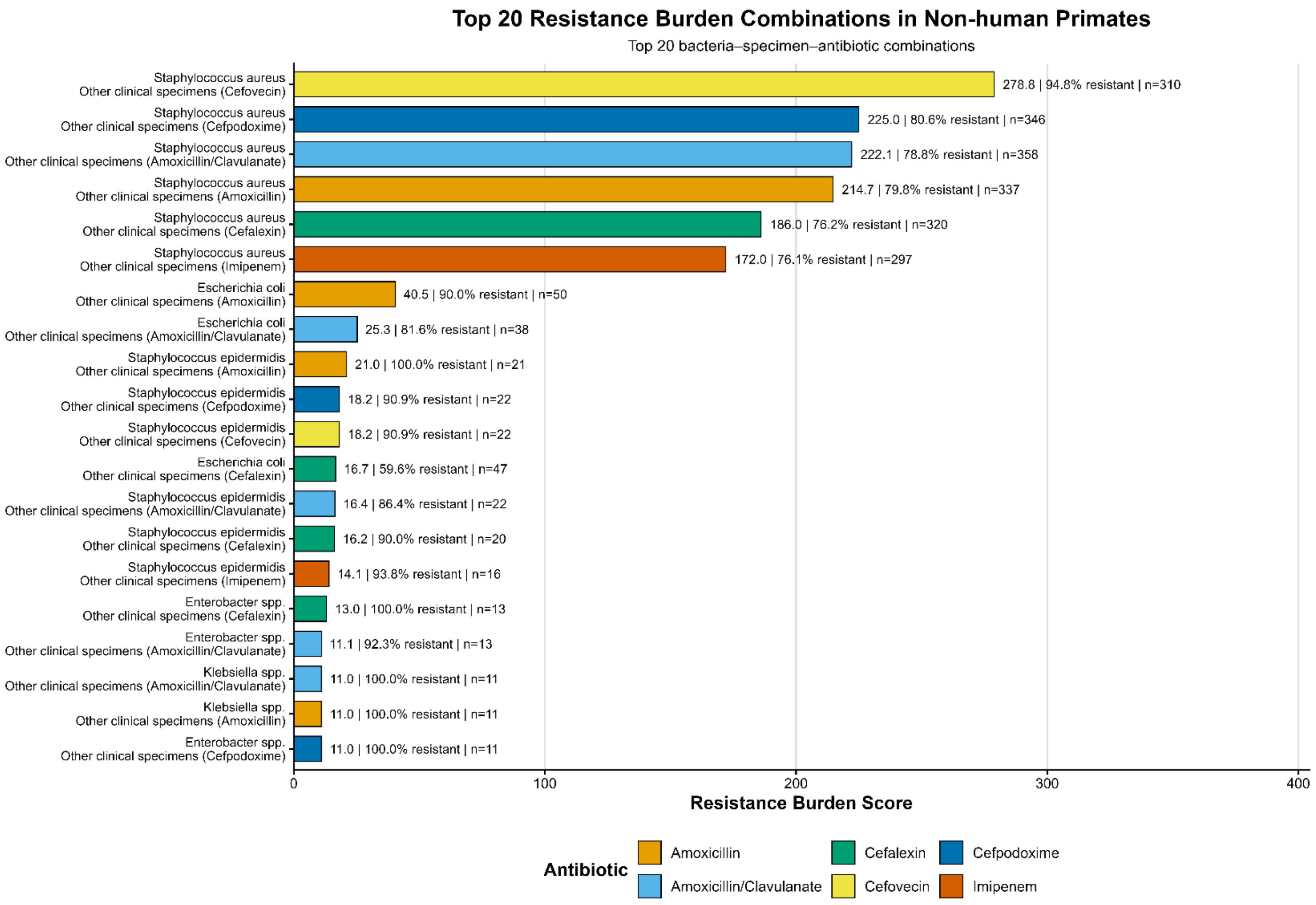

Figure S6. Top 20 bacterial group–clinical specimen group–antimicrobial combinations ranked by Resistance Burden Score among non-human primates.

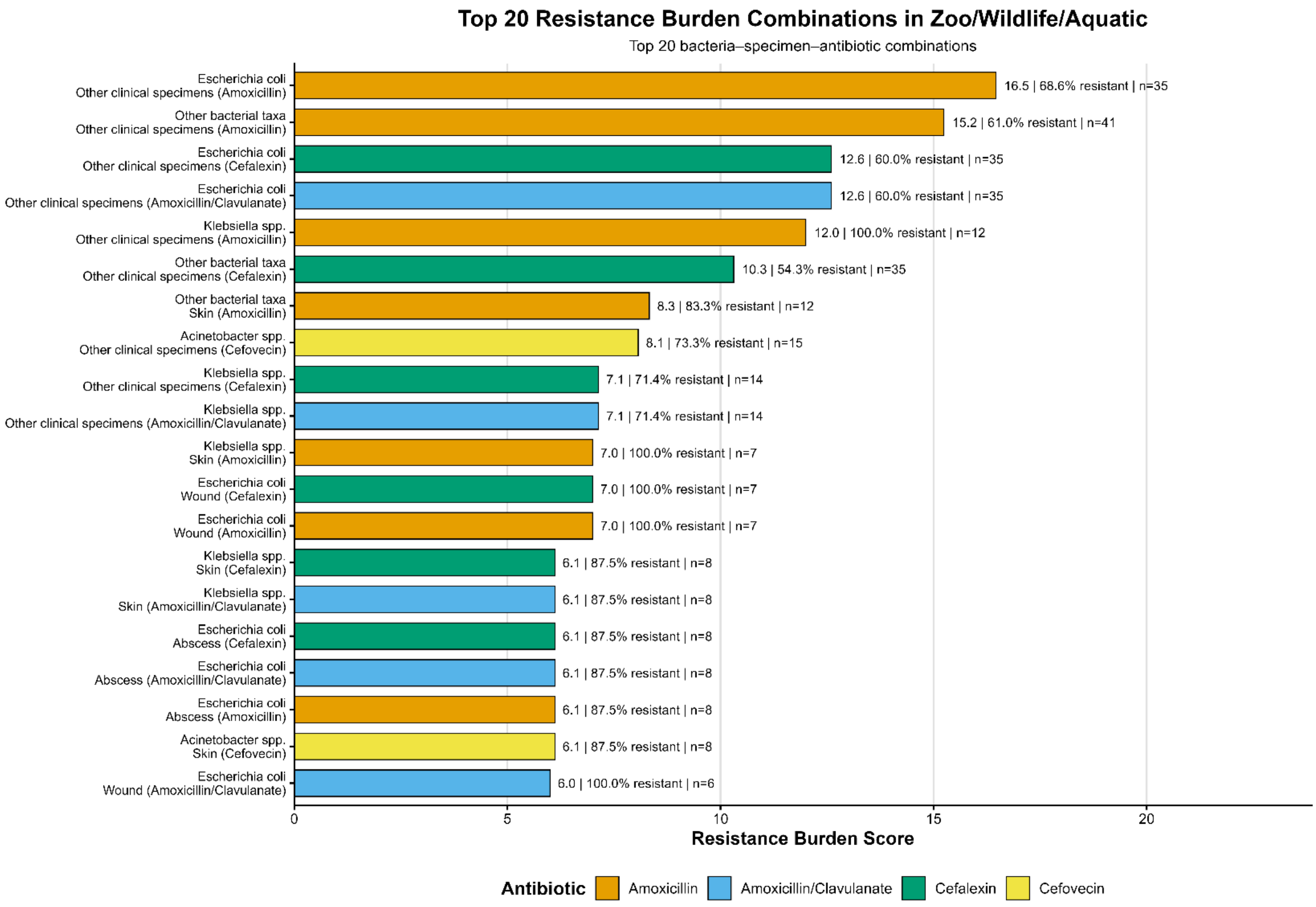

Figure S7. Top 20 bacterial group–clinical specimen group–antimicrobial combinations ranked by Resistance Burden Score among zoo, wildlife, and aquatic species.
